# Supplementary material for: Structural analysis identifies an escape route from the adverse lipogenic effects of liver X receptor ligands
Source: Commun Biol. 2019 Nov 22;2:431. doi: 10.1038/s42003-019-0675-0 (PMC6874530; doi:10.1038/s42003-019-0675-0)
Supplement: Supplementary file 1 — Supplementary Information [file 42003_2019_675_MOESM1_ESM.pdf]

Supplementary Table 1: Differences in deuteration levels (% of maximal deuteration) of LXRα and LXRβ peptides upon ligand binding.

Related to Figure 2

Deuterium uptake values for all peptides were corrected to back exchange using the fully deuterated controls. Missing peptides are shown in dark grey. HDX-MS measurements were performed in triplicates and the average values are shown. Color scheme is identical to Figure 2.

		T0-091317		WAY-254011		F1		AZ876		AZ1		AZ2		AZ3		AZ4	
		ΔHDX (apo - ligand), %															
Peptide	Secondary Structure	30 sec	600 sec	30 sec	600 sec	30 sec	600 sec	30 sec	600 sec	30 sec	600 sec	30 sec	600 sec	30 sec	600 sec	30 sec	600 sec
**LXRα**																	
200-210	H1	3,7	-1,4	-1,7	-1,9	2,3	-3,5	2,3	-2,1	4,9	0,2	5,3	-3,3	7,5	0,7	4,8	2,3
200-213	H1	0,0	0,9	-3,2	-2,0	-4,7	-5,4	-0,2	-0,7	-1,6	-3,1	4,1	1,4	6,3	2,8	-1,4	-1,7
204-213	H1	-1,6	2,0	-2,9	0,9	-0,5	-2,1	-3,5	1,4	1,0	1,3	2,7	2,0	4,9	4,5	-2,4	-0,1
207-213	H1	3,8	9,9	-11,0	-3,3	-1,3	0,8	-5,3	0,3	-11,3	-12,4	-11,7	-11,0	4,7	3,0	-4,3	-2,1
208-213	H1	1,1	6,8	-5,7	-2,2	-7,7	-8,8	-4,7	-0,5	-6,3	-6,4	-7,5	-8,4	1,5	2,7	-5,2	-1,9
214-219	H1	-10,4	-27,8	-1,6	-12,0	-14,5	-30,9	-15,4	-36,7	-12,6	-29,7	-12,0	-27,0	-8,2	-18,2	-8,6	-24,7
214-233	H1	-8,8	-5,7	-3,6	-4,4	-9,7	-8,5	-12,2	-13,2	-6,2	-11,5	-9,8	-7,9	-7,3	-5,7	-12,6	-11,2
220-233	H1	-2,5	-0,3	-1,4	1,1	-2,0	1,9	-1,4	1,1	0,4	1,4	-2,0	0,3	-3,9	-2,7	-7,0	-4,7
221-233	H1	-0,5	1,8	-0,3	0,8	0,1	2,2	0,0	1,1	1,9	2,0	-0,2	1,4	-6,0	0,0	-5,6	-0,2
226-233	H1	2,8	4,3	-2,2	-1,0	1,6	2,9	0,9	-0,2	-1,0	-3,2	-5,0	-2,8	-4,3	-3,1	-6,0	-3,2
234-240	loop H1-H3	-2,2	-0,2	-8,2	-4,8	-5,5	-4,1	-4,9	-1,6	-3,9	-3,2	-2,1	-2,9	-3,0	-2,4	-2,4	-2,9
234-248	loop H1-H3	4,5	2,9	-2,9	-3,4	-3,1	1,8	-9,9	0,1	-1,1	0,9	-7,4	-3,0	-9,2	-1,8	-11,7	-4,7
234-254	loop H1-H3, H3	3,4	1,7	-3,2	-1,0	-5,4	1,0	-8,0	-0,1	-0,1	0,9	-4,7	-0,6	-5,2	1,1	-6,3	-2,6
234-257	loop H1-H3, H3	-6,8	2,7	-7,6	-2,8	-6,1	0,7	-15,0	-0,6	-2,8	-0,5	-9,7	-0,5	-8,7	-2,8	-12,8	-4,8
239-257	loop H1-H3, H3	-6,9	5,5	-9,5	-2,2	-4,2	5,9	-14,0	-0,9	-6,3	-2,0	-16,2	-4,1	-11,1	-5,0	-13,4	-5,5
241-257	loop H1-H3, H3	0,5	3,5	-3,0	-3,8	0,8	2,0	-0,4	-2,6	-0,9	-5,0	-2,8	-2,2	-3,7	-3,8	-6,7	-5,7
241-259	loop H1-H3, H3	-0,1	2,9	-8,8	-6,7			-0,8	-5,1	-3,1	-4,4	-1,5	-4,8	-1,3	-2,2	-3,8	-3,8
249-257	H3	-15,6	4,9	-15,6	-6,1	-9,6	0,1	-25,4	-0,2	-7,9	3,1	-16,4	-2,2	-13,0	-3,0	-19,2	-6,6
260-265	H3	-34,5	-42,6	-28,8	-44,3	-41,7	-45,6	-31,3	-46,0	-27,6	-24,3	-24,8	-23,3	-32,1	-27,7	-34,3	-29,9
261-267	H3	-31,5	-40,2	-26,7	-40,3	-35,0	-38,3	-31,6	-42,4	-22,7	-35,3	-21,2	-20,2	-32,9	-28,8	-32,6	-31,9
264-269	H3	-36,0	-32,2	-31,0	-37,5	-34,2	-34,6	-35,1	-38,1	-25,2	-31,1	-24,0	-15,6	-35,5	-24,9	-35,3	-28,6
270-275	H3	-32,2	-26,0	-11,8	-11,9	-27,2	-17,2	-38,1	-27,4	-28,7	-20,7	-25,6	-8,3	-29,4	-18,0	-30,1	-22,1
271-278	H3	-0,5	-5,4	3,5	4,1	-2,2	-8,5	-0,9	-6,7	-1,2	-7,5	-1,9	-4,9	-2,0	-5,0	-2,2	-4,5
272-278	H3-H4	-0,3	-5,0	3,7	5,0	-1,8	-8,0	-0,2	-5,1	-1,6	-5,5	-1,7	-3,5	-0,8	-4,7	-1,8	-4,4
273-278	H3-H4	0,7	-5,8	5,1	6,1	-3,3	-12,8	-0,2	-11,0	1,1	-12,2	-1,6	-7,8	-2,7	-9,5	-2,0	-10,8
271-281	H3-H4	0,7	-3,0	6,4	7,0	-2,1	-4,6	-2,4	-4,3	-0,2	-5,1	0,3	-2,6	-2,0	-5,6	-1,5	-4,1
272-281	H3-H4	-0,3	-2,7	5,9	6,1	-2,6	-6,1	-2,2	-5,4	-1,8	-5,5	-0,8	-3,0	-0,1	-3,8	-0,3	-3,4
279-286	H4-H5	-9,1	-10,4	1,3	2,4	-1,5	-0,6	-0,4	-3,2	0,2	-3,1	-0,9	-2,5	-2,8	-3,2	-3,4	-3,2
280-290	H4-H5	-0,9	-2,1	3,6	4,1	-0,2	-3,7	-0,4	-5,1	0,4	-5,9	0,5	-3,1	-3,4	-4,4	-4,2	-4,3
282-289	H4-H5	-1,4	-3,4	3,1	7,6	-0,1	-5,0	-0,9	-3,5	0,0	-3,4	0,0	-2,7	-2,5	-3,2	-2,5	-2,9
282-290	H4-H5	-0,9	-3,4	3,6	7,7	0,5	-6,0	0,0	-3,7	0,4	-4,1	1,0	-2,1	-3,2	-4,6	-3,0	-3,2
285-290	H5	-3,1	-5,7	2,2	6,5	-0,1	-1,3	0,7	-3,0	-0,3	-3,0	-0,3	-2,8	-0,8	-3,2	-2,8	-4,6
290-296	H5	-14,6	-34,7	-5,8	-25,6	-17,6	-22,0	-13,9	-34,4	-12,4	-31,8	-9,2	-21,9	-24,0	-30,7	-24,6	-31,7
299-311	H5-βsheets	-9,5	-17,4	-1,3	-12,0	-14,5	-24,6	-10,7	-14,8	-10,4	-18,4	-10,1	-10,7	-14,9	-18,8	-17,1	-26,3
299-312	H5-βsheets	-12,1	-15,6	-3,2	-9,6	-18,6	-25,1	-12,6	-12,9	-10,6	-16,2	-9,8	-7,5	-16,5	-17,2	-18,7	-22,3
300-311	H5-βsheets	-10,5	-18,8	-2,2	-13,7	-15,4	-25,1	-11,3	-15,8	-11,2	-19,5	-11,2	-11,9	-14,9	-17,9	-16,6	-25,4
300-312	H5-βsheets	-12,8	-17,6	-2,8	-13,3	-17,1	-24,3	-14,7	-16,2	-12,5	-18,8	-11,8	-9,8	-18,1	-19,0	-19,4	-25,5
299-314	H5-βsheets	-17,8	-17,0	-7,5	-14,4	-20,3	-24,9	-16,7	-15,8	-15,5	-19,2	-15,7	-8,7	-22,0	-18,0	-23,5	-24,0
299-315	H5-βsheets	-21,2	-16,6	-8,9	-15,9	-19,8	-21,4	-18,7	-15,3	-17,0	-21,4	-17,1	-7,7	-24,2	-18,2	-25,2	-24,5
300-315	H5-βsheets	-21,8	-17,0	-9,3	-16,6	-23,5	-21,6	-19,3	-15,7	-17,7	-22,5	-16,4	-9,0	-26,9	-18,8	-27,6	-25,4
312-316	βsheets	-24,9	-0,7	-25,9	-18,2	-22,2	-7,0	-28,2	-1,7	-36,6	-15,6	-33,1	-3,2	-38,9	-9,7	-42,5	-15,4
316-326	βsheets-H6	-20,9	-12,4	-12,0	-10,9	-16,5	-12,4	-14,9	-12,8	-12,7	-10,3	-11,7	-1,5	-20,7	-11,9	-25,8	-14,1
319-324	βsheets-H6	-27,8	-14,9	-21,2	-16,1	-15,0	-4,7	-18,7	-9,1	-18,4	-14,8	-25,0	-3,9	-17,1	-16,0	-22,9	-19,1
319-326	βsheets-H6	-30,7	-22,1	-14,4	-16,7	-23,9	-21,7	-25,9	-19,4	-18,7	-17,0	-17,8	-5,5	-27,4	-16,6	-29,7	-19,1
320-326	βsheets-H6	-30,5	-23,6	-15,2	-19,1	-24,5	-18,1	-29,4	-19,8	-21,7	-18,8	-21,6	-6,2	-27,2	-16,8	-29,6	-17,2
321-326	βsheets-H6	-29,5	-22,2	-21,0	-26,4	-21,7	-24,7	-30,1	-27,9	-20,8	-18,7	-22,0	-11,7	-29,7	-22,3	-35,9	-25,1
319-331	βsheets-H6	-39,8	-29,1	-10,8	-15,0	-30,2	-19,9	-35,3	-26,0	-26,2	-22,0	-25,4	-7,7	-35,2	-18,5	-36,4	-21,0
320-331	βsheets-H6	-41,0	-28,5	-16,5	-20,8	-32,7	-19,2	-43,4	-34,7	-33,3	-29,3	-32,6	-14,0	-37,4	-22,0	-38,5	-23,1
321-331	βsheets-H6	-45,4	-29,4	-8,4	-22,0			-68,0	-41,8	-43,2	-18,2	-36,9	-12,3	-32,1	-17,4	-34,0	-16,2
326-331	H6	-44,1	-28,9	-11,3	-12,8	-37,3	-18,1	-49,4	-30,4	-36,1	-22,6	-30,6	-6,6	-44,7	-19,9	-45,2	-21,9
327-331	H6	-43,8	-31,0	-14,2	-13,5	-37,6	-19,6	-51,6	-29,6	-38,0	-23,5	-31,8	-9,5	-43,9	-21,5	-43,5	-23,5
333-341	H7	-23,1	-50,0	-17,6	-33,5	-36,2	-50,7	-24,1	-59,3	-24,2	-56,7	-29,1	-36,9	-29,9	-44,7	-29,4	-44,5
334-340	H7	-20,2	-51,9	-11,3	-40,8	-21,1	-49,1	-21,5	-53,5	-19,7	-54,8	-17,0	-43,9	-21,0	-45,6	-14,5	-32,2
334-341	H7	-19,5	-48,5	-7,1	-31,8	-25,9	-47,7	-21,0	-42,9	-16,4	-42,3	-17,4	-32,8	-25,3	-44,0	-27,2	-44,4
335-340	H7	-23,0	-45,8	-6,8	-29,6	-22,2	-47,0	-22,9	-41,1	-20,4	-38,9	-19,2	-31,4	-27,3	-44,4	-25,9	-42,6
335-341	H7	-20,3	-48,7	-3,5	-30,4	-19,9	-50,6	-19,1	-43,2	-16,8	-40,5	-15,6	-33,4	-25,7	-46,4	-24,3	-45,4
336-341	H7	-9,0	-49,6	2,2	-35,0	-17,5	-63,2	-9,9	-49,2	-9,5	-48,6	-9,3	-44,0	-16,5	-52,0	-16,4	-52,2
341-348	H7	-18,2	-42,7	-0,2	-23,0	-19,9	-41,8	-17,4	-34,9	-14,4	-36,9	-12,6	-28,2	-28,3	-35,3	-29,3	-37,0

	GW3965		BMS-852927		AZ6		AZ7		LXR-623		AZ8		AZ9		AZ5		24,25EC	
	ΔHDX (apo - ligand), %																	
Peptide	30 sec	600 sec	30 sec	600 sec	30 sec	600 sec	30 sec	600 sec	30 sec	600 sec	30 sec	600 sec	30 sec	600 sec	30 sec	600 sec	30 sec	600 sec
**LXRα**																		
200-210	-12,1	-4,3	1,5	-0,3	9,1	2,3	-1,6	-2,0	7,3	-0,3	6,5	1,3	3,3	2,6	7,4	2,2	2,4	-3,5
200-213	-7,3	-3,3	2,9	0,0	2,4	0,9	-2,0	-2,0	8,1	3,8	4,2	-0,1	-1,1	-1,5	5,9	1,6	2,9	0,5
204-213	-5,6	-2,8	2,3	0,7	7,5	10,7	-2,2	-1,9	6,3	4,6	5,3	3,1	-0,6	1,2	5,2	3,0	4,5	2,2
207-213	-6,9	-2,5	1,3	2,0	-5,2	-7,0	2,7	6,6	9,1	6,8	-1,8	-3,4	-4,5	-3,4	5,8	0,6	2,6	1,7
208-213	-4,2	-2,2	0,7	0,8	-5,1	-3,8	-6,9	-1,1	5,2	4,4	-0,1	-2,7	-4,6	-1,1	5,5	2,9	2,6	2,1
214-219	10,0	6,9	-8,4	-13,1	-7,6	-18,1	-6,7	-10,2	-4,2	-8,6	0,4	0,5	-8,2	-14,3	-0,4	0,1	-7,5	-12,6
214-233	1,8	0,0	-4,5	-2,6	-11,6	-8,2	-6,8	-4,7	-3,3	-3,8	-0,5	-1,4	-2,1	-3,5	-0,1	-0,5	-4,7	-3,7
220-233	-2,0	-1,3	-1,6	-2,1	-6,7	-4,9	-4,4	-3,9	-3,5	-3,6	-2,4	-3,1	1,6	2,2	1,0	0,0	-1,8	0,9
221-233	-1,3	-0,2	0,5	0,6	-5,2	-1,3	-4,6	-3,1	-5,7	-3,1	-2,3	0,0	2,9	1,8	3,2	0,9	-0,1	1,9
226-233	-3,8	-2,0	-0,9	-2,3	-8,5	-9,1	-14,7	-14,6	-4,9	-6,0	-2,1	-2,5	-0,8	-1,4	-6,6	-3,7	-1,4	0,4
234-240	-3,3	0,3	0,5	-0,1	-3,2	-1,9	-4,5	-0,5	-1,0	-1,4	-2,2	-1,5	-2,9	-2,4	-0,3	-1,4	-0,3	0,8
234-248	-1,9	-1,8	2,3	1,3	-8,7	-5,6	-13,4	-8,4	-1,4	-2,1	-5,2	-0,5	0,6	-0,7	-0,4	-2,4	-1,2	-0,2
234-254	-9,1	-5,3	3,4	-0,4	-3,5	-5,0	-13,9	-19,7	-1,8	-2,1	3,0	-0,9	3,2	-2,1	-1,3	-0,6	-1,6	-0,8
234-257	-8,0	-2,4	0,5	2,1	-10,5	-4,6	-9,0	-7,3	-4,2	-2,4	-1,8	-3,2	0,4	-0,4	-0,5	-0,5	-1,3	0,5
239-257	-10,3	-2,9	0,9	2,9	-10,7	-5,7	-9,0	-7,5	-1,2	-1,3	-0,3	-2,5	-0,8	0,6	-1,8	-2,1	-1,7	0,2
241-257	-10,7	-3,0	2,2	0,9	-7,8	-7,1	-2,8	-2,8	-2,5	-3,8	-1,8	-3,0	-0,6	-0,5	0,1	-1,2	-1,0	0,1
241-259	-14,0	-8,9	-0,9	-1,6	-0,2	-0,9	0,8	-2,2	-2,4	-5,2	3,1	1,4	-1,8	-0,5	-0,8	-0,7	-0,7	-3,8
249-257	-18,5	-6,0	-2,0	3,1	-14,7	-5,6	-15,3	-11,0	-6,7	-2,8	-2,7	-3,6	0,4	-1,1	-1,0	0,9	-1,8	0,1
260-265	-31,3	-49,5	-44,9	-77,5	-31,7	-9,1	-42,9	-34,0	-17,9	-9,8	-12,2	12,9	-5,8	-0,3	-2,3	3,1	-9,9	-14,7
261-267	-30,7	-40,7	-44,9	-68,9	-29,0	-18,8	-36,1	-31,7	-20,6	-11,8	-9,7	-1,1	-5,3	-6,3	-5,4	-2,8	-10,3	-9,9
264-269	-34,1	-34,6	-50,1	-69,5	-30,3	-15,4	-40,3	-30,9	-20,5	-8,4	-7,5	-1,1	-6,2	-2,2	-4,3	-1,9	-10,2	-5,8
270-275	-17,8	-14,8	-20,7	-6,4	-27,5	-10,1	-25,6	-20,0	-11,6	-7,4	-9,6	-3,4	-8,7	-0,6	-7,2	-4,5	-12,6	-0,8
271-278	7,1	17,6	-1,2	-7,5	-2,2	-4,6	-1,5	-5,6	-1,5	-3,4	-1,7	-2,8	-0,6	-3,9	-1,1	-3,2	-0,3	-3,7
272-278	7,4	16,9	-0,8	-6,1	-1,7	-4,5	-1,7	-5,2	-1,4	-4,1	-1,1	-2,7	-0,7	-2,0	-1,2	-3,1	-0,6	-2,2
273-278	14,1	30,8	-1,8	-11,9	-3,7	-9,9	-0,7	-10,9	-2,5	-5,8	-2,6	-6,3	-0,2	-8,0	-2,5	-9,1	0,8	-4,3
271-281	13,4	19,2	-0,2	-6,2	-2,2	-5,1	0,4	-3,2	-1,0	-5,6	-2,6	-3,5	-0,1	-2,9	-1,2	-3,0	0,0	-1,3
272-281	12,1	15,8	0,4	-5,1	-1,3	-4,9	0,1	-2,8	2,7	-1,9	-2,0	-4,1	1,4	-2,4	0,3	-2,9	-0,2	-2,2
279-286	2,7	7,4	-0,6	-2,6	-5,3	-5,4	-4,7	-2,8	-2,3	-3,7	-3,2	-3,8	-0,3	-1,1	-0,8	-2,2	-0,3	-0,4
280-290	5,1	12,5	-1,1	-4,2	-4,7	-4,6	-3,0	-3,0	-2,5	-3,8	-3,0	-4,6	-0,1	-3,9	-1,4	-4,1	0,6	-2,2
282-289	3,2	16,1	-0,7	-4,9	-3,6	-3,6	-0,9	-3,0	-2,4	-3,6	-2,1	-2,4	-0,5	-2,0	-0,6	-1,9	0,2	-1,6
282-290	4,7	18,2	-1,1	-6,0	-3,4	-3,9	-1,1	-2,8	-2,5	-3,5	-2,7	-3,7	0,4	-1,7	-0,9	-2,4	0,3	-1,3
285-290	2,6	20,7	0,2	-3,6	-1,0	-4,2	-3,8	-1,7	-1,3	-2,9	-0,9	-3,8	-0,4	-3,8	1,8	-0,5	0,7	-1,6
290-296	-2,6	-19,1	-14,8	-11,3	-23,9	-25,5	-21,8	-31,5	-17,1	-14,8	-13,0	-7,1	-5,7	-9,6	-9,3	-6,1	-5,5	-11,1
299-311	3,2	-8,7	-13,2	-27,1	-16,5	-21,8	-13,0	-17,2	-4,1	-5,7	-10,5	-7,3	-6,2	-4,4	-7,4	-5,4	-6,2	-5,3
299-312	2,6	-7,4	-16,9	-29,5	-16,8	-18,1	-15,2	-17,4	-5,3	-4,6	-10,1	-5,9	-4,8	-3,4	-6,5	-4,6	-5,5	-5,6
300-311	1,5	-11,4	-14,3	-28,7	-17,5	-21,7	-14,4	-19,1	-3,8	-4,8	-9,9	-7,2	-6,7	-5,0	-9,0	-9,2	-7,2	-6,2
300-312	1,8	-10,7	-18,4	-31,4	-12,4	-20,1	-16,0	-18,8	-5,3	-4,9	-6,8	-4,4	-5,2	-4,0	-4,9	-2,6	-6,5	-4,7
299-314	-0,6	-13,1	-25,2	-35,9	-21,3	-17,0	-19,7	-14,9	-10,0	-5,9	-9,8	-5,2	-4,4	-1,5	-8,0	-4,1	-7,5	-3,6
299-315	-5,1	-15,1	-29,3	-38,5	-23,7	-17,2	-20,9	-13,3	-11,2	-5,4	-10,2	-5,3	-4,0	-2,1	-7,4	-4,0	-8,8	-3,8
300-315	-5,6	-15,6	-31,3	-39,9	-25,9	-17,2	-22,3	-13,5	-11,8	-4,3	-11,4	-5,1	-5,3	-3,5	-8,2	-3,6	-7,6	-3,6
312-316	-20,3	-14,3	-46,6	-37,9	-37,5	-6,0	-34,0	-9,3	-18,3	-3,8	-8,6	-0,1	-4,3	-0,3	-7,1	-0,3	-11,7	0,0
316-326	-6,8	-9,1	-26,5	-27,6	-24,7	-8,7	-22,0	-13,1	-13,7	-5,2	-5,8	-1,9	-2,2	0,8	-5,8	-1,7	-3,7	1,0
319-324	-10,2	-11,0	-28,5	-29,5	-18,9	-4,8	-27,8	-8,5	-13,3	-8,3	-2,7	-1,4	-8,6	-3,2	-1,6	2,2	-7,3	0,0
319-326	-10,3	-15,7	-27,8	-35,4	-28,2	-11,3	-24,3	-15,2	-18,9	-6,6	-9,0	-2,7	-3,8	1,5	-7,2	-2,8	-9,0	0,3
320-326	-10,7	-17,9	-30,7	-37,7	-30,5	-9,9	-33,0	-20,9	-19,7	-11,3	-5,9	0,8	-5,4	1,6	-7,5	-4,6	-11,2	1,9
321-326	-10,3	-19,4	-27,5	-38,5	-32,0	-16,7	-37,6	-25,4	-21,7	-9,2	-11,4	5,2	-5,6	0,6	-12,9	-7,1	-10,9	0,5
319-331	-12,6	-17,0	-32,8	-22,6	-33,8	-11,7	-29,8	-12,2	-19,4	-6,2	-10,7	-1,5	-4,5	0,6	-9,7	-3,7	-13,8	-1,9
320-331	-20,4	-23,1	-35,3	-23,8	-36,2	-14,8	-33,2	-14,2	-19,0	-7,1	-11,4	-1,2	-10,1	0,5	-12,9	-7,5	-14,3	-0,9
321-331	-12,1	-14,9	-35,7	-21,9	-35,9	-15,9	-41,3	-19,7	-13,8	-4,9	-5,9	0,9	3,1	1,3	-12,2	-10,2	-21,2	-2,0
326-331	-19,5	-11,7	-26,9	-5,1	-40,4	-12,2	-29,2	-10,2	-20,8	-7,5	-14,9	-6,4	-7,4	0,1	-11,4	-5,6	-15,8	-0,9
327-331	-19,6	-12,1	-31,9	-5,6	-43,5	-12,8	-32,6	-9,2	-21,2	-9,4	-19,3	-2,3	-10,0	0,6	-14,5	-2,3	-16,5	-0,2
333-341	-6,4	-22,3	-21,1	-42,2	-29,2	-39,0	-25,0	-43,8	-22,7	-23,6	-12,4	-18,5	-15,4	-15,3	-14,1	-10,3	-3,7	-16,6
334-340	2,3	-8,3	-16,1	-47,2	-35,4	-48,4	-27,9	-56,7	-12,1	-21,6	-10,3	-7,9	-7,7	-16,8	-10,8	-20,0	-3,9	-19,1
334-341	5,5	-16,2	-19,2	-47,8	-24,3	-41,0	-22,9	-48,8	-19,5	-24,5	-9,5	-10,1	-10,7	-24,4	-11,1	-12,7	-7,8	-20,3
335-340	2,8	-13,3	-11,7	-40,7	-25,3	-39,5	-17,8	-42,1	-20,1	-28,4	-10,2	-15,7	-10,1	-25,5	-9,9	-13,5	-7,1	-22,7
335-341	6,1	-17,1	-14,5	-46,7	-23,8	-42,1	-16,7	-44,8	-19,3	-27,4	-16,6	-15,5	-8,8	-26,0	-11,0	-12,8	-6,8	-25,4
336-341	13,7	-18,3	-14,3	-59,1	-17,0	-50,2	-12,8	-54,4	-14,8	-32,6	-13,2	-18,7	-8,0	-34,3	-10,0	-15,1	-5,0	-32,3
341-348	9,5	-13,5	-22,2	-44,9	-29,4	-33,1	-24,1	-40,0	-24,1	-18,2	-21,1	-7,3	-10,0	-19,6	-15,5	-7,7	-9,2	-18,2

342-348	H7	-18,5	-40,8	-5,2	-27,6	-21,6	-35,0	-17,1	-38,7	-16,6	-39,3	-14,4	-31,9	-29,2	-36,8	-28,8	-37,0	8,4	-15,5	-20,1	-40,8	-29,3	-33,4	-21,2	-34,6	-24,3	-19,4	-20,2	-9,2	-13,2	-24,3	-14,6	-7,5	-8,8	-18,6
349-353	loop H7-H8	-5,0	-16,1	6,4	-8,3	-6,1	-4,1	-2,5	-12,3	-1,3	-13,3	-1,0	-8,4	-11,0	-8,1	-13,7	-14,2	13,7	0,7	-2,9	-21,4	-15,5	-14,5	-4,3	-21,6	-8,0	-6,5	-6,7	-4,2	-0,2	-6,8	-5,2	-4,7	-1,5	-10,3
349-355	loop H7-H8, H8	-0,6	-9,3	2,3	-2,7	-0,4	-3,3	-3,5	-8,3	-1,2	-9,7	1,4	-6,1	-7,0	-7,0	-7,1	-7,6	11,9	3,6	-4,6	-13,3	-8,1	-11,1	-5,5	-12,2	-6,5	-4,9	-4,7	-4,6	-2,0	-5,0	-3,4	-1,9	-1,2	-5,2
349-356	loop H7-H8, H8	-2,1	-8,4	2,6	1,3	-1,5	-10,0	-7,4	-14,1	-3,2	-9,0	0,0	-4,2	-2,8	-2,9	-5,2	-6,9	8,0	7,1	-3,5	-10,2	-4,3	-6,5	-4,8	-9,9	-3,7	-4,1	-1,1	-1,3	-3,1	-5,0	-1,5	-2,0	-1,8	-6,6
350-356	loop H7-H8, H8	-0,1	-2,7			-1,0	-6,0	0,5	-3,4	-1,5	-6,8	1,1	-3,1	0,6	-1,3	-3,2	-4,0	5,0	12,1	0,5	-5,2	-1,4	-4,0	-0,8	-4,2	2,0	-2,2	0,4	-2,2			-1,7	-2,4	0,4	-2,3
352-356	H8	1,1	-2,9	0,7	3,4	-1,3	-5,1	-0,9	-4,4	0,8	-5,0	2,0	-2,6	2,9	-3,2	2,3	-2,0	4,6	13,7	1,1	-4,7	2,1	-2,9	-1,4	-3,4	3,3	-2,3	1,1	-2,3	-0,7	-3,3	0,1	-1,4	2,4	-0,9
357-361	H8	-0,4	-1,7	-0,5	0,3	0,9	-2,3	0,3	-1,0	-0,1	-0,9	0,0	-1,2	0,0	0,0	0,4	0,2	0,5	5,9	-0,2	-2,5	0,3	0,2	1,2	-0,7	0,3	1,2	0,5	0,4	-0,2	-0,6	0,7	0,0	-0,1	-1,0
360-365	H8	-0,4	-3,8	1,5	0,4	-0,5	-6,0	0,0	-4,0	1,0	-3,9	1,1	-2,8	-0,8	-1,6	-0,8	-1,3	1,9	9,3	-0,8	-4,6	-0,8	-1,7	-0,6	-3,5	-0,7	-0,9	-0,4	-1,3	0,8	-2,5	-0,2	-1,4	-0,2	-3,1
362-367	H8, loop H8-H9	-3,2	-5,7	2,0	-1,5	-0,6	-1,9	-1,9	-3,8	3,1	-1,6	2,3	-3,0	-4,3	-5,3	-4,9	-7,2	9,4	14,6	2,4	-5,1	-3,3	-5,3	-7,9	-7,1	2,6	-3,5	-3,3	-5,0	1,4	-3,2	-3,3	-5,4	-1,5	-1,1
362-376	H8, loop H8-H9	-9,6	-10,1	1,6	-7,1	-11,9	-5,4	-10,4	-11,5	-7,3	-13,5	-7,2	-6,1	-14,2	-9,0	-14,3	-9,9	10,6	-2,5	-4,9	-2,7	-14,1	-7,6	-11,4	-6,9	-11,4	-5,7	-9,2	-2,4	-4,7	-3,4	-6,9	-2,7	-4,4	-4,4
366-376	loop H8-H9, H9	-12,4	-13,3	-6,2	-7,7	-16,4	-5,0	-19,2	-12,7	-15,2	-10,3	-15,3	-6,2	-19,5	-13,9	-20,8	-12,5	9,1	-3,1	-6,5	0,2	-19,3	-9,8	-14,4	-7,1	-14,9	-7,0	-13,1	-3,9	-11,2	-4,8	-9,2	-3,6	-10,5	-2,2
366-377	loop H8-H9, H9	-12,5	-13,2	-2,2	-6,8	-8,4	-1,8	-19,5	-12,2	-13,3	-9,8	-14,3	-6,2	-18,4	-11,4	-17,9	-10,0	10,6	-3,8	-6,6	0,6	-18,5	-9,9	-14,6	-8,7	-14,2	-7,0	-12,4	-2,6	-7,8	-4,0	-9,1	-3,1	-4,0	-3,0
366-378	loop H8-H9, H9	-11,2	-13,3	0,9	-5,1	-13,7	-5,1	-15,7	-13,1	-9,5	-9,1	-6,9	-3,7	-16,4	-12,3	-15,7	-11,2	11,6	0,1	-6,4	-1,4	-16,6	-10,4	-14,2	-10,5	-12,6	-8,0	-10,8	-3,9	-5,7	-3,5	-7,8	-3,2	-4,7	-2,1
366-389	loop H8-H9, H9	-5,0	-9,8	-2,7	-3,5	-6,6	-2,7	-8,3	-10,6	-6,7	-10,1	-6,2	-3,6	-11,7	-10,1	-11,5	-10,2	19,7	10,1	-3,6	-5,6	-11,3	-8,1	-6,1	-6,3	-9,8	-8,4	-8,2	-5,8	-7,3	-4,6	-6,1	-3,8	-2,5	-3,1
377-385	H9	0,7	-5,8	11,0	4,6	3,4	-5,4	-3,0	-8,0	-2,1	-10,4	-1,3	-5,6	-4,4	-4,2	-3,8	-3,3	24,2	20,3	-1,2	-6,5	-3,2	-3,2	-1,0	-1,8	-7,2	-7,1	-4,3	-5,7	-1,6	-5,3	-2,7	-5,6	-0,2	-3,8
377-386	H9	-1,7	-7,4	11,9	6,2	-1,8	-10,0	-0,6	-6,1	0,0	-5,8	-0,8	-3,1	-5,2	-6,8	-5,1	-4,7	23,1	21,4	-2,1	-7,5	-4,9	-5,7	-0,7	-2,3	-5,0	-4,7	-4,7	-4,8	-0,2	-3,4	-1,8	-3,0	-0,1	-3,5
377-387	H9	-1,7	-7,8	9,8	7,2	-1,1	-9,2	-2,3	-6,8	-2,7	-8,3	-1,5	-4,7	-3,9	-5,4	-3,0	-4,2	21,8	20,0	-2,7	-8,0	-3,9	-5,4	-1,2	-3,5	-4,4	-5,4	-3,9	-4,8	-2,1	-4,5	-2,7	-4,1	-0,4	-3,3
377-389	H9	-1,4	-6,3	8,3	6,6	-0,9	-9,1	-2,0	-6,0	-2,0	-7,0	-1,7	-4,7	-2,9	-4,4	-2,4	-3,8	18,3	19,5	-2,1	-7,8	-2,9	-4,1	-0,8	-3,6	-3,2	-4,2	-2,9	-3,7	-1,6	-3,8	-1,9	-3,1	-0,4	-3,0
378-389	H9	-1,2	-8,1	4,3	6,7	0,9	3,3	-2,8	-6,8	0,2	-9,5	-1,7	-8,7	-3,1	-5,6	-2,0	-4,4	7,9	18,9	-1,5	-2,2	-6,3	-7,6	0,0	2,4	-5,7	-7,8	-3,6	-4,5	-1,4	-8,3	-1,2	-3,5	0,1	-7,5
379-389	H9	-2,0	-6,2	9,5	7,9	-0,4	-3,4	-2,0	-5,7	-1,4	-6,0	-1,0	-3,9	-2,0	-3,0	-0,9	-2,3	18,4	22,7	-2,0	-8,8	-2,5	-2,6	-2,1	-4,7	-3,1	-3,7	-2,1	-2,4	-1,0	-3,1	-0,8	-3,8	-0,1	-3,6
382-389	H9	-1,6	-8,0	4,9	7,5	-1,5	-4,5	-2,5	-7,8	-2,5	-9,7	-2,1	-4,3	-3,6	-5,1	-3,7	-4,5	17,7	23,2	-1,8	-10,6	-2,7	-5,9	-2,6	-8,8	-1,5	-3,7	-3,0	-5,2	-3,2	-5,3	-3,5	-4,9	0,1	-2,7
385-389	H9	-3,4	1,3	4,2	9,9	-7,3	-8,9	-2,2	-7,1	-1,5	-8,3	-2,8	-7,1	-1,8	-3,6	0,1	-4,9	16,4	25,6	-3,1	-8,0	-1,5	-3,7	-4,2	-8,0	-4,3	-5,3	1,2	-4,2	-1,4	-5,2	1,1	-3,5	-1,0	-2,4
390-402	H9-H10	3,9	0,5	-3,9	-6,2	3,1	1,2	-3,6	-8,0	-4,1	-5,9	-4,1	-6,7	-0,3	-8,5	-0,1	-5,2	2,6	5,6	-0,6	-4,9	-2,9	-9,0	-3,6	-13,5	3,2	-3,0	3,6	-3,2	-1,9	-4,8	1,6	-1,3	-1,2	-1,7
390-404	H9-H10	-0,7	-1,3	-0,8	-2,2	-0,7	-4,1	-4,2	-5,1	-3,1	-6,1	-2,8	-5,2	-2,2	-3,7	-3,1	-5,4	3,4	2,1	-1,2	-3,1	-3,6	-5,6	-3,1	-4,0	0,1	-2,5	-0,4	-2,5	-1,9	-2,4	-0,3	-1,6	-0,2	-1,7
392-402	H9-H10	-6,2	-5,9	-3,3	-9,9	8,2	-1,1	-5,2	-7,6	-1,0	-13,9	-2,8	-8,6	-4,1	-12,6	-2,4	-9,5	-2,8	-6,9	-2,8	-10,4	-8,1	-17,0	-18,3	-24,6	-5,1	-3,9	-7,7	-10,9	1,4	-1,5	-8,1	-3,8	-1,4	-2,5
392-404	H9-H10	-1,9	-1,4	-1,5	-3,6	-3,1	-4,2	-3,6	-5,8	-3,6	-5,4	-3,6	-5,1	-2,6	-2,8	-4,5	-5,2	2,9	-1,2	-0,9	-2,1	-5,4	-6,0	-4,4	-4,6	0,3	-0,2	-0,6	-0,8	-1,7	-1,1	0,6	-0,6	-0,9	-1,5
393-404	H9-H10	1,2	2,4	-4,4	1,1	-2,4	-6,9	-10,1	-4,5	-9,3	0,2	-8,5	-3,5	-4,5	-4,9	-8,8	-9,0	1,4	0,9	0,6	-1,0	-8,0	-9,0	-3,0	-2,8	-2,7	-3,3	-2,5	-3,3	-6,3	0,1	1,0	-1,9	-1,1	1,0
390-408	H9-H10	-3,1	0,0	7,5	-0,8	-2,5	-3,1	-2,6	-9,3	-0,9	-10,3	-2,5	-8,6	-3,8	-9,0	-6,5	-10,2	10,2	7,0	-4,8	-4,8	-7,1	-7,4	-9,2	-10,2	-2,7	-5,3	-4,8	-3,1	4,0	-9,6	0,7	-0,8	-1,2	-5,3
392-407	H9-H10	-2,0	-6,2	2,8	-2,1	-3,2	-9,0	-3,3	-8,8	-2,6	-9,8	-3,0	-4,8	-2,2	-6,5	-4,6	-9,4	7,2	6,5	-1,6	-2,1	-2,4	-5,2	-4,2	-6,5	-1,3	-5,1	1,9	-1,7	-0,3	-4,5	-0,2	-1,9	-3,0	-3,2
392-409	H9-H10	-1,9	-5,3	-5,6	-2,4	-0,9	-6,6	-4,2	-9,6	-8,4	-9,6	-5,0	-6,4	-1,9	-11,3	-1,6	-10,1	4,4	2,3	-1,8	-2,3	-4,2	-9,9	-4,1	-7,3	-1,7	-10,4	-0,5	-5,1	0,8	-5,9	-1,0	-4,3	-3,6	-5,3
408-413	H10/11	0,7	1,2	-3,7	-3,7	-1,0	-3,7	-1,6	-4,8	-0,8	-7,5	0,6	0,4	-2,1	-9,7	-0,6	-3,0	-4,9	-2,0	1,0	0,1	-0,5	-3,6	-2,8	-5,3	-5,4	-11,6	-1,9	-6,7	-2,2	-7,4	-0,8	-5,2	-0,6	-3,0
410-414	H10/11	0,1	-1,7	-2,1	-5,3	-2,8	-8,2	-1,4	-6,0	-2,0	-9,0	-0,3	-2,9	-3,7	-12,7	-1,6	-6,5	-2,9	-1,3	0,9	6,3	-1,3	-7,5	-3,4	-9,5	-7,7	-12,7	-3,2	-7,2	-2,1	-6,9	-1,7	-5,4	-2,2	-4,9
412-420	H10/11	-16,9	-2,5	-10,0	-5,5	-2,9	-3,8	-15,5	-4,1	-14,3	-10,1	-14,1	-3,0	-20,8	-10,4	-15,5	-4,2	-0,2	-0,8	6,3	0,6	-14,2	-3,4	-9,4	-4,3	-23,0	-11,3	-9,8	-6,3	-8,7	-4,2	-6,4	-4,4	2,9	-1,4
415-420	H10/11	-31,8	-2,8	-19,1	-3,5	-3,1	3,0	-27,4	-0,6	-24,7	-5,5	-24,4	0,5	-34,8	-6,9	-25,3	-1,0	-6,5	-4,0	8,7	1,7	-25,1	0,4	-15,7	-4,5	-32,9	-10,7	-15,1	-3,8	-11,9	0,5	-8,9	-5,0	6,8	-1,3
412-424	H10/11	-30,6	-3,6	-12,7	-6,7	-9,6	-7,0	-20,9	-5,1	-11,9	-8,9	-16,1	-1,5	-31,6	-10,0	-24,8	-4,8	-6,4	-4,2	-0,7	-1,1	-21,4	-7,4	-13,6	-2,1	-29,2	-13,7	-14,4	-6,0	-6,4	-5,1	-7,3	-8,5	4,7	-4,2
412-426	H10/11	-30,7	-4,6	-16,6	-4,0	-5,7	-3,8	-30,0	-7,1	-22,7	-9,7	-23,3	-0,8	-32,9	-9,9	-27,3	-6,0	-5,6	-2,3	3,5	0,6	-22,9	-4,3	-9,2	-1,4	-27,0	-9,9	-12,6	-6,6	-9,2	-1,1	-5,9	-2,9	-0,5	-0,4
412-427	H10/11	-33,3	-6,2	-17,7	-5,3	-6,0	-3,8	-31,7	-8,6	-23,4	-10,8	-22,5	-0,7	-33,9	-9,3	-28,5	-6,0	-6,3	-3,3	3,0	0,7	-23,4	-4,0	-10,3	-1,8	-27,6	-10,0	-12,0	-5,8	-8,8	-2,1	-5,9	-2,9	-0,8	-0,7
414-426	H10/11	-35,0	-4,1	-20,6	-3,2	-5,7	-1,6	-34,9	-6,5	-27,0	-7,1	-26,4	0,0	-34,8	-4,6	-28,8	-2,8	-8,8	-3,6	3,6	-0,9	-24,6	-1,2	-10,8	0,1	-29,4	-7,6	-12,4	-4,2	-10,7	0,2	-5,1	-0,5	-2,1	-0,1
414-427	H10/11	-37,6	-4,2	-23,7	-3,9	-5,4	-3,9	-40,9	-8,7	-30,0	-7,4	-29,6	-0,8	-37,1	-5,1	-31,8	-4,1	-11,2	-6,0	2,9	-0,4	-26,7	-2,0	-11,5	-0,8	-30,3	-7,8	-11,8	-3,7	-11,9	-0,1	-5,9	-0,9	-3,5	-0,5
415-426	H10/11	-40,2	-5,0	-19,6	-2,5	-7,0	-1,0	-36,1	-7,5	-28,8	-5,9	-26,7	0,7	-40,3	-5,6	-34,2	-3,7	-9,6	-4,1	2,6	-1,4	-28,4	-1,9	-12,1	-0,1	-31,0	-8,1	-12,3	-3,4	-9,6	0,8	-6,6	-1,0	-2,8	-1,0
415-427	H10/11	-43,8	-5,7	-21,2	-3,5	-7,3	-1,6	-39,9	-7,4	-28,3	-5,8	-29,8	0,0	-40,6	-5,9	-34,9	-4,2	-9,2	-3,6	2,2	-1,0	-28,9	-2,3	-12,9	-0,8	-30,6	-7,8	-12,5	-3,9	-9,3	0,8	-6,3	-1,1	-2,7	-0,1
427-439	H10/11-H12	-11,9	1,6	-7,0	-0,8	-2,8	-1,3	-13,8	-1,5	-7,7	-1,8	-10,0	-1,7	-11,4	-0,6	-9,7	-1,4	-3,4	-1,1	0,1	-0,8	-10,1	-3,3	-2,5	-1,4	-8,0	-1,3	-2,9	-1,7	-2,6	0,4	-1,0	-0,1	-0,7	0,3
427-440	H10/11-H12	-14,4	1,3	-7,4	-0,7	-3,7	-1,7	-14,7	-2,0	-8,9	-3,6	-9,5	-1,0	-13,8	-1,7	-11,7	-1,5	-1,4	-0,7	0,6	-0,5	-10,4	-2,8	-3,1	-1,3	-10,5	-2,6	-3,0	-1,5	-3,5	-0,7	-1,9	-1,1	-0,6	-0,3
428-439	H10/11-H12	-10,4	1,7	-5,9	-1,8	-3,1	-1,4	-11,0	-1,2	-7,7	-3,3	-10,2	-2,9	-11,0	-2,6	-10,6	-3,6	-3,1	-1,6	0,4	-0,4	-10,3	-4,6	-3,6	-2,3	-7,5	-2,1	-4,1	-3,2	-2,5	-0,2	-2,3	-2,3	-1,2	-0,3
427-441	H10/11-H12	-15,6	1,5	-7,4	-0,5	-2,5	-1,7	-16,7	-0,8	-9,4	-1,8	-10,0	-0,2	-15,9	-2,6	-13,2	-3,3	-3,3	-1,0	-0,4	-0,3	-10,3	-3,0	-1,7	-0,1	-10,4	-2,4	-4,2	-2,7	-2,1	0,3	-2,3	-1,3	-1,7	-0,5
428-440	H10/11-H12	-12,9	4,4	-8,0	-1,5	-3,8	-0,1	-15,3	-1,3	-10,1	-3,9	-11,1	-0,4	-13,6	-2,3	-13,0	-3,3	-3,6	-1,6	0,5	-0,3	-10,4	-3,3	-1,2	0,9	-9,5	-2,5	-3,5	-2,0	-3,2	0,5	-1,9	-0,3	-1,4	0,4
427-442	H10/11-H12	-19,8	1,4	-4,1	-1,2	0,5	0,2	-18,0	1,0	-10,8	-3,5	-11,2	2,4	-8,9	0,5	-13,2	-0,1	-3,3	-1,4	0,3	0,0	-12,1	-1,5	-2,0	1,9	-10,2	-0,2	-1,9	0,8	-3,2	-0,1	-0,7	-1,4	-2,4	-0,4
428-441	H10/11-H12	-14,1	3,9	-7,7	-1,3	-2,2	0,2	-17,1	-2,7	-11,0	-3,5	-11,7	-2,6	-15,3	-2,3	-13,2	-2,0	-4,0	-3,4	0,9	-0,2	-10,9	-3,4	-2,1	-0,5	-10,7	-1,6	-2,9	-2,6	-3,5	-0,7	-4,1	-1,1	-2,0	-0,4
427-443	H10/11-H12	-24,4	1,1	-9,0	-0,5	0,7	2,0	-26,5	-1,8	-16,1	-4,0	-17,4	-1,1	-22,2	0,7	-18,4	-1,1	-1,7	-1,8	1,0	-0,2	-15,1	-0,8	-5,7	0,5	-8,3	1,3	-4,6	-0,5	-5,2	0,6	-2,8	0,3	-1,0	-0,6
428-442	H10/11-H12	-20,3	1,9	-5,3	-1,4	3,5	3,9	-19,1	0,7	-12,5	-4,5	-14,1	2,2	-13,8	3,2	-15,6	-3,2	-3,3	-1,8	1,0	0,1	-11,4	-1,1	-1,0	2,4	-10,0	0,1	-2,2	2,7	-2,6	2,8	-2,4	0,5	-1,7	-0,4
428-443	H10/11-H12	-23,2	3,6	-9,2	-1,7	5,8	4,8	-24,9	-2,4	-16,7	-5,0	-17,8	-1,6	-22,2	-0,7	-20,7	-1,7	-2,1	-1,5	1,5	-0,3	-19,0	-1,7	-2,7	-0,3	-16,5	-2,8	-3,1	-0,1	-5,7	-0,2	-5,0	-2,0	-1,2	0,0
440-447	H12	-26,2	-1,6	-13,1	-1,3	-7,9	-2,5	-29,4	-3,1	-21,8	-6,3	-21,7	-1,2	-26,3	-3,2	-23,7	-4,0	-0,1	-0,7	0,6	-0,3	-20,0	-2,0	-8,6	-1,4	-18,4	-3,7	-7,7	-2,1	-9,3	0,0	-5,0	-1,3	-1,2	0,3
441-446	H12	-27,3	0,4	-21,7	-5,9	-12,6	-6,0	-16,6	-0,5	-36,3	2,1	-28,7	-4,0	-26,0	-0,2	-21,7	-5,1	-4,0	-0,5	0,2	-0,4	-17,6	2,8	-4,2	0,0	-13,3	-4,5	-4,3	0,3	-8,0	0,9	-1,0	4,1	-0,6	3,1
443-447	H12	-1,6	0,4	-1,3	1,1	-4,0	-2,3	0,4	-1,0	-2,0	-3,3	-1,0	-0,4	-1,8	-0,7	-3,7	-2,4	1,3	0,4	-0,1	-0,3	-4,0	-2,5	-2,2	-1,9	-2,7	-1,3	0,1	-1,0	-1,6	0,2	0,4	0,1	-1,2	0,9
**LXRβ**																																			
218-222	H1	2,1	0,4	-2,1	1,7	0,0	-1,2	-0,4	1,3	-0,5	2,0	2,9	1,8	2,0	-0,9	-0,2	0,5	-1,5	1,6	2,4	4,2	3,5	2,5	4,0	2,9	-1,4	3,9	-1,1	3,0	-3,7	3,5	1,5	4,1	0,6	2,4
218-223	H1	1,2	0,2	-2,8	-0,3	1,8	-1,2	-2,0	0,2	2,0	5,0	0,2	-1,0	0,2	-0,4	-4,1	5,1	-1,8	-0,2	-1,4	1,9	0,0	1,4	0,0	0,6	-4,1	1,8	1,5	3,3	-0,5	3,8	2,3	6,4	3,0	4,9
218-225	H1	1,4	0,1	1,1	1,1	-0,9	-1,0	-0,3	0,7	-0,3	-1,6	1,4	0,4	1,7	-1,9	-2,5	-1,9	4,2	0,4	-1,8	-2,2	1,8	0,6	2,7	0,3	-3,2	-0,2	-1,0	1,1	-3,5	-1,4	-0,9	-2,0	1,0	1,0
218-226	H1	1,1	-1,3	4,9	4,2	1,1	2,5	-1,0	0,9	-3,3	-7,6	-0,2	-3,1	1,5	-4,1	-1,4	0,4	7,3	4,9	-2,2	-1,5	0,7	4,1	0,1	0,7	-4,0	-1,4	-1,1	0,3	-1,4	-3,3	1,6	-2,5	-2,3	-6,4
221-226	H1	0,2	0,2	6,6	4,9	1,9	3,9	-0,8	1,9	-1,4	-10,4	-0,1	-6,1	1,5	-4,0	-3,5	-5,1	10,5	7,1	-1,5	-6,4	2,6	2,8	2,3	0,0	-3,8	-1,6	0,0	-4,3	-1,8	-9,0	0,4	-9,9	0,7	-4,8
221-227	H1	4,7	1,0	14,6	11,2	12,6	1,9	5,3	2,9	0,8	-7,8	1,4	-8,5	4,3	-1,5	-4,5	-7,2	19,3	13,1	-2,3	-14,8	3,0	-1,8	2,7	-2,0	-0,8	-0,8	-0,7	-14,7	-0,9	-16,8	1,2	-16,8	2,8	-1,7
226-230	H1	0,1	-1,8	13,2	18,1	1,8	2,1	-2,1	-4,1	0,1	-11,3	0,2	-8,5	0,7	-2,5	-2,6	-8,0	16,6	32,8	0,1	-12,4	0,9	-3,1	1,1	-4,1	-0,7	-8,9	-0,9	-8,0	-0,6	-10,2	-0,4	-11,7	-0,6	-7,9
226-231	H1	-0,1	-2,0	12,3	19,8	1,8	0,0	-0,5	-4,0	-0,8	-9,4	-0,8	-9,8	-0,1	-3,6	-0,8	-5,2	16,8	34,1	-1,4	-10,7	0,4	-4,2	0,2	-5,5	-0,9	-7,5	-0,3	-5,1	0,2	-7,8	0,3	-8,2	0,2	-5,9
227-231	H1	0,4	-0,9	1,1	2,0	0,2	-3,5	-1,9	-2,6	-0,8	-2,8	-2,9	-4,7	-0,4	-1,5	4,1	-2,9	3,8	8,5	0,1	-5,0	-0,3	-4,3	-0,1	-4,7	-2,5	-3,2	2,1	-10,6	0,0	-8,5	-3,9	-12,2	1,9	-7,4
226-233	H1	-0,7	-2,1	12,2	27,8	1,8	-1,2	-0,8	-3,4	-0,9	-10,0	-1,6	-8,2	-0,9	-3,8	-0,5	-4,3	17,6	40,9	-0,9	-9,3	-0,1	-3,3	-0,2	-4,3	-0,5	-6,6	0,6	-6,5	0,4	-8,9	0,8	-9,1	-0,4	-6,8
227-233	H1	-0,1	-2,3	11,7	21,4	1,1	-2,9	-0,2	-3,2	-0,5	-7,0	-0,5	-5,9	-0,2	-2,6	-0,3	-4,7	15,4	36,7	0,0	-6,6	0,2	-3,0	-0,4	-3,2	-0,5	-5,4	-0,8	-5,1	-0,7	-6,6	0,1	-6,7	-0,4	-4,9
228-233	H1	0,2	-2,2	12,6	24,1	2,7	-3,8	0,2	-4,1	0,0	-6,5	1,2	-5,6	0,3	-2,2	-0,4	-7,7	16,1	38,3	0,9	-6,2	1,1	-3,1	1,9	-2,7	0,6	-4,4	0,0	-5,4	0,8	-5,6	0,8	-6,3	0,3	-5,2
232-236	H1	-5,9	-24,1	14,3	-20,9	-10,2	-14,7	-4,2	-13,8	-6,5	-47,7	-10,1	-44,9	-6,4	-34,9	-8,1	-15,3	24,3	-0,7	-9,8	-45,0	-5,5	-22,6	-5,4	-25,6	-7,9	-28,7	-3,8	-26,8	-6,0	-39,6	-5,6	-45,6	-3,6	-34,8
234-238	H1	-3,2	-14,2	-0,3	-16,2	-4,4	-8,0	-2,3	-5,9	-2,9	-23,6	-6,9	-25,4	-3,2	-20,8	-6,3	-2,8	4,1	-8,0	-6,2	-23,2	-4,7	-11,7	-3,7	-13,3	-5,5	-12,3	-0,8	-11,9	-6,3	-17,7	-5,5	-22,5	-4,2	-16,6

274-279	H3	-15,4	-53,6	-18,7	-45,8	-33,2	-73,4	-20,6	-45,6	-19,1	-79,3	-23,1	-62,0	-15,4	-54,9	-40,4	-84,4	-20,6	-45,0	-24,6	-63,7	-19,2	-31,4	-19,2	-32,2	-21,2	-53,4	-15,9	-46,7	-18,0	-61,3	-17,9	-65,7	-12,1	-45,7
275-279	H3	-23,8	-54,7	-29,2	-50,0	-34,5	-65,3	-27,4	-50,7	-25,4	-72,6	-31,3	-72,5	-23,0	-57,8	-44,7	-46,4	-24,1	-45,7	-30,6	-73,2	-29,7	-35,1	-30,0	-36,5	-29,5	-66,8	-20,8	-36,4	-25,8	-50,9	-23,1	-55,8	-16,3	-33,2
274-281	H3	-17,7	-49,9	-15,4	-47,2	-35,0	-64,9	-16,8	-48,9	-17,8	-66,1	-18,8	-67,1	-17,8	-51,3	-41,2	-56,2	-14,5	-41,2	-18,7	-66,0	-15,6	-33,5	-15,6	-34,6	-16,5	-55,0	-11,7	-33,8	-15,2	-50,9	-15,2	-54,2	-9,5	-39,3
275-281	H3	-16,5	-50,8	-19,8	-48,7	-34,0	-63,9	-22,1	-49,2	-23,3	-68,5	-23,1	-63,8	-16,3	-50,4	-41,0	-48,2	-19,9	-42,2	-23,1	-66,0	-19,7	-31,5	-19,1	-33,0	-22,1	-53,8	-18,3	-32,9	-21,5	-49,6	-21,6	-54,7	-14,9	-31,7
279-284	H3	-1,6	-7,3	4,1	4,4	-0,9	-18,8	1,6	-11,6	-1,9	-13,2	0,3	-16,1	-0,8	-7,5	-8,1	-13,6	3,1	7,0	-0,7	-13,8	0,6	-8,5	-0,3	-8,4	-1,1	-13,1	-3,3	-8,3	-3,7	-11,3	-1,0	-11,6	-0,5	-9,2
280-284	H3	-0,8	-2,1	4,8	6,2	0,5	-7,9	3,7	-2,4	-0,6	-3,9	3,4	-5,5	2,9	-1,2	-0,6	-5,7	6,1	6,5	1,8	-6,1	8,3	1,3	11,4	6,3	1,6	-5,6	0,4	-3,8	0,7	-3,3	0,6	-3,0	1,9	-2,0
280-285	H3	0,5	-2,1	1,5	6,4	0,3	-5,8	-0,6	-3,0	0,0	-2,4	0,7	-3,3	1,0	-1,0	0,8	-7,2	3,1	10,7	-0,4	-4,2	-0,7	-3,3	-0,4	-2,1	0,4	-1,9	-1,4	-2,6	-0,7	-1,7	-0,3	-2,2	-0,5	-1,7
281-285	H3	-0,5	-0,3	3,2	13,5	2,4	-2,8	-0,2	-2,9	0,2	-2,3	-1,7	-3,4	0,6	0,8	-0,5	-8,2	3,6	17,0	-1,0	-4,8	-1,2	-3,6	-0,7	-3,8	-1,3	-4,0	0,4	-1,7	0,2	-1,1	1,0	-1,5	0,3	-1,2
285-292	H3, loop H3-H4	-0,6	-1,6	4,1	13,4	0,3	-1,8	-0,3	-1,0	1,5	-0,6	-1,5	-3,2	-2,2	-3,1	-0,5	-2,3	5,3	23,9	-1,8	-2,9	-1,3	-2,4	-2,2	-2,9	-2,0	-3,2	0,7	-0,6	0,0	-1,0	1,6	0,0	0,9	-0,7
285-293	H3, loop H3-H4	-0,4	-0,6	8,2	13,2	1,7	0,1	1,1	3,3	-2,4	-1,8	-4,1	0,3	0,4	-4,1	-1,8	0,6	10,5	21,4	-6,1	-4,2	-5,7	1,4	-5,0	-1,7	-4,6	1,2	-2,8	-2,6	-3,1	-7,2	-3,6	-6,7	-2,8	-3,0
286-292	H3, loop H3-H4	0,7	0,0	4,4	12,9	1,5	-1,2	-0,4	-1,7	2,1	0,9	-1,1	-3,4	-0,4	-1,1	0,0	-1,4	5,6	22,7	-1,5	-2,7	-1,2	-2,5	-1,1	-2,2	-0,6	-1,7	1,5	-0,2	2,2	0,3	1,4	-0,2	2,3	1,0
285-295	H3, loop H3-H4	-0,9	-1,1	8,7	14,1	2,1	-0,9	0,2	-0,2	-0,8	-1,2	0,1	-1,6	-0,1	-1,7	-1,6	-0,6	11,6	20,7	-0,5	-0,7	2,1	-0,1	0,7	-0,6	-1,2	-0,4	-1,3	-0,8	-1,4	-1,7	-1,1	-1,7	-1,7	-1,2
286-295	H3, loop H3-H4	0,1	-0,6	6,9	11,4	2,0	-1,2	-0,1	0,1	-0,8	-1,7	0,3	-0,8	0,6	-1,6	-1,4	-1,0	8,7	17,0	0,3	-0,5	2,5	0,2	0,7	-0,5	-0,6	-0,1	-2,0	-1,8	-1,6	-2,6	-1,7	-2,4	-1,6	-1,4
293-300	H4-H5	1,2	0,1	4,3	7,0	2,0	0,1	1,0	1,8	-0,6	1,5	1,5	2,7	1,0	-1,6	-1,2	1,1	3,3	8,2	0,6	1,4	1,9	2,6	1,3	2,1	0,5	2,6	-1,6	0,3	-1,6	0,7	0,1	1,3	-1,4	1,4
294-299	H4-H5	1,5	0,7	6,5	5,7	0,6	0,1	3,5	0,2	-1,7	-2,4	3,4	2,8	2,1	0,3	-2,8	-1,9	5,6	7,2	2,8	0,0	2,5	8,1	0,6	5,8	2,1	0,5	-4,8	3,4	-2,5	3,6	4,1	5,1	-0,7	-1,1
294-304	H4-H5	2,5	1,0	6,5	13,9	2,3	0,6	0,8	1,1	0,4	0,9	2,4	3,8	2,6	-0,4	-1,2	-0,1	5,8	17,7	1,2	1,5	2,0	2,9	1,8	2,6	1,4	2,4	-3,0	-0,2	-1,8	0,7	-1,3	-0,9	-1,3	1,2
296-303	H5	0,9	0,6	6,1	13,2	2,0	0,3	0,4	-0,6	0,9	0,5	2,2	2,1	1,1	0,9	-1,0	-1,5	6,1	19,2	0,8	0,1	1,4	1,0	1,4	0,5	0,6	0,9	-0,6	-0,1	0,0	-0,1	0,7	-0,8	0,2	1,1
296-304	H5	0,0	0,1	5,4	16,2	2,3	1,5	1,6	0,5	0,6	0,7	3,2	-5,4	0,6	0,1	-0,6	-1,0	6,2	22,1	2,4	2,4	3,1	2,5	3,2	2,9	2,7	2,9	0,1	0,8	-0,2	0,4	0,8	-0,1	0,5	0,5
304-308	H5	-13,9	-32,2	-12,5	-45,0	-26,0	-35,2	-20,5	-50,0	-22,1	-67,8	-20,1	-56,6	-15,4	-45,9	-37,8	-41,3	-10,4	-33,1	-12,7	-10,6	-22,1	-45,2	-18,7	-39,5	-20,3	-39,1	-17,5	-32,7	-20,2	-44,8	-21,7	-60,5	-13,9	-34,4
304-310	H5	-18,4	-26,3	-16,9	-36,3	-32,7	-22,8	-23,7	-40,8	-26,5	-57,6	-25,2	-44,9	-19,6	-35,8	-36,3	-31,0	-13,2	-21,8	-8,3	-6,7	-22,5	-31,8	-22,3	-31,7	-22,4	-30,6	-18,9	-23,7	-21,3	-33,7	-23,8	-48,8	-17,6	-26,5
305-310	H5	-17,9	-26,8	-15,5	-35,6	-30,7	-29,4	-21,9	-40,7	-23,7	-53,2	-22,6	-43,3	-19,0	-35,4	-27,6	-30,3	-12,3	-18,0	-5,8	-7,1	-21,0	-30,4	-20,9	-32,1	-20,6	-32,8	-17,0	-21,6	-19,4	-32,1	-21,8	-45,1	-17,1	-26,8
313-325	H5, βsheets	-11,6	-13,0	-3,0	-10,8	-16,5	-21,8	-9,0	-17,3	-16,1	-27,8	-14,3	-25,7	-16,1	-24,7	-16,8	-19,3	-1,1	-2,0	-16,5	-28,2	-12,2	-9,9	-11,7	-10,6	-6,9	-2,9	-8,4	-7,6	-11,4	-9,3	-10,5	-15,6	-8,6	-13,4
313-326	H5, βsheets	-15,8	-13,0	-9,5	-15,7	-19,9	-19,2	-14,8	-15,9	-20,9	-31,1	-20,5	-29,7	-19,6	-27,1	-17,8	-20,0	-6,4	-6,0	-21,2	-31,3	-16,1	-12,2	-15,0	-12,0	-11,6	-4,9	-10,7	-9,1	-15,6	-10,8	-16,6	-16,9	-9,5	-11,6
314-326	H5, βsheets	-16,9	-13,9	-11,1	-18,4	-20,1	-18,4	-15,1	-17,9	-22,5	-32,1	-20,0	-28,7	-21,6	-27,7	-19,4	-19,7	-7,6	-8,7	-20,0	-31,3	-16,5	-10,9	-14,5	-10,6	-10,4	-5,2	-11,4	-7,5	-16,1	-8,9	-16,1	-15,8	-10,0	-11,1
330-340	βsheets, H6	-20,8	-14,1	-29,7	-31,1	-28,6	-12,5	-15,8	-7,9	-39,7	-37,7	-35,7	-32,9	-29,9	-23,7	-33,7	-17,5	-24,4	-24,4	-38,3	-38,4	-20,8	3,7	-17,5	3,9	-29,7	-11,4	-14,2	2,4	-29,0	-2,3	-29,5	-8,7	-10,6	1,6
333-339	βsheets, H6	-30,3	-26,3	-30,1	-35,1	-29,4	-15,3	-27,3	-11,5	-33,4	-37,6	-35,2	-37,6	-30,0	-30,7	-20,8	-6,9	-24,1	-28,3	-34,5	-44,4	-25,3	-0,7	-23,4	0,8	-31,1	-17,5	-17,2	-2,6	-26,7	-8,1	-27,2	-10,3	-11,4	-3,2
333-340	βsheets, H6	-29,9	-23,1	-27,6	-34,8	-29,9	-15,9	-25,4	-12,3	-35,2	-38,3	-32,0	-37,9	-30,8	-29,1	-32,4	-20,1	-21,2	-28,7	-36,9	-45,0	-20,9	-0,3	-17,1	0,0	-29,5	-15,0	-13,9	2,0	-25,4	-3,6	-27,2	-8,1	-10,7	2,2
335-340	βsheets, H6	-29,7	-23,8	-29,0	-35,2	-29,5	-14,5	-25,3	-10,9	-37,1	-43,0	-31,7	-37,5	-29,6	-29,7	-23,3	-19,8	-24,8	-28,0	-36,5	-46,2	-22,8	0,9	-19,9	3,3	-29,2	-16,4	-21,7	-5,6	-32,7	-7,9	-28,6	-10,5	-13,5	-0,8
333-345	βsheets, H6	-43,5	-25,6	-29,9	-29,4	-44,1	-14,1	-37,2	-13,2	-48,5	-47,6	-44,6	-40,7	-44,2	-32,7	-42,8	-24,1	-25,4	-22,2	-26,7	-18,0	-30,1	0,8	-23,8	2,5	-24,6	-8,2	-22,7	1,0	-33,7	-4,3	-37,2	-9,1	-17,6	-2,6
333-346	βsheets, H6	-46,7	-25,8	-39,6	-28,1	-48,4	-11,9	-42,4	-10,4	-54,4	-51,8	-45,7	-36,2	-47,5	-34,0	-50,4	-27,7	-35,3	-16,6	-26,8	-19,8	-34,4	8,3	-27,4	8,9	-34,4	-11,0	-27,7	1,7	-39,1	-3,3	-43,1	-9,6	-21,0	-1,9
334-345	βsheets, H6	-61,9	-28,1	-38,6	-35,8	-44,4	-3,8	-48,9	-18,8	-50,3	-49,7	-49,0	-44,2	-66,8	-26,0	-57,4	-28,2	-34,4	-29,6	-36,6	-26,3	-42,4	-4,5	-35,3	-4,3	-34,5	-19,2	-39,2	1,8	-53,9	-9,6	-37,0	-6,9	-18,2	-0,4
341-346	H6-H7	-51,7	-24,4	-43,0	-33,5	-64,8	-11,4	-53,0	-14,0	-68,0	-61,0	-62,9	-52,3	-52,6	-29,9	-72,8	-33,3	-31,9	-19,4	-18,4	2,6	-48,1	-4,7	-39,4	-2,1	-41,4	-10,0	-32,0	-0,3	-45,2	-6,9	-50,2	-10,5	-25,3	-4,1
341-347	H6-H7	-45,0	-19,8	-33,8	-25,4	-44,7	-7,8	-40,7	-8,6	-53,4	-46,1	-51,6	-43,9	-46,3	-27,6	-51,1	-23,3	-27,0	-17,2	-13,2	3,3	-41,7	-5,0	-32,2	-2,4	-34,7	-9,8	-26,7	-3,1	-38,7	-7,2	-41,2	-7,2	-21,3	0,6
341-348	H6-H7	-43,2	-20,1	-29,1	-24,4	-37,8	-7,7	-35,3	-8,9	-48,7	-35,8	-44,1	-36,6	-43,9	-24,6	-42,8	-16,2	-25,3	-18,5	-6,6	-3,5	-38,5	-2,0	-31,5	-1,5	-27,4	-9,7	-22,2	2,0	-35,7	0,0	-32,6	-0,1	-20,0	1,4
341-349	H6-H7	-42,2	-26,9	-26,1	-26,9	-41,5	-9,8	-41,0	-13,9	-52,5	-45,9	-48,4	-42,1	-43,1	-30,5	-42,9	-21,4	-23,7	-21,5	-10,9	-1,3	-34,9	-0,3	-28,3	-3,5	-33,2	-13,3	-23,3	-1,6	-39,2	-8,0	-39,6	-10,9	-20,7	-2,6
347-355	H7	-25,8	-43,5	-30,6	-62,8	-38,6	-47,1	-33,0	-50,9	-36,7	-71,4	-37,6	-79,1	-26,4	-45,4	-37,6	-52,2	-28,6	-49,8	-18,7	-40,0	-33,7	-31,9	-30,0	-32,9	-33,7	-55,9	-26,9	-21,4	-34,1	-41,9	-30,0	-45,9	-19,9	-24,9
348-355	H7	-22,2	-42,3	-17,2	-47,6	-34,4	-54,1	-27,7	-48,3	-27,2	-65,2	-29,4	-68,9	-21,7	-42,7	-42,8	-42,2	-14,8	-35,5	-17,9	-39,9	-25,1	-29,8	-19,9	-30,3	-26,1	-47,3	-16,8	-26,8	-23,7	-42,7	-23,6	-44,5	-13,9	-28,3
349-354	H7	-24,7	-42,5	-20,3	-46,0	-30,2	-45,4	-25,9	-43,9	-28,8	-65,5	-28,8	-61,9	-24,1	-42,5	-37,4	-42,4	-16,8	-28,8	-6,0	-36,6	-23,1	-29,5	-20,4	-31,7	-24,4	-44,8	-15,5	-31,0	-23,6	-39,9	-22,9	-40,3	-13,1	-30,6
349-355	H7	-25,2	-48,1	-20,7	-48,4	-32,4	-49,6	-26,6	-46,4	-28,9	-67,5	-30,6	-64,5	-23,7	-48,6	-38,9	-39,6	-17,2	-33,6	-12,0	-42,5	-23,9	-31,3	-22,1	-33,0	-25,7	-47,1	-17,0	-31,0	-23,2	-43,0	-24,4	-45,6	-14,0	-30,8
350-354	H7	-5,8	-43,6	-5,5	-43,8	-17,2	-63,7	-11,9	-55,0	-11,5	-66,4	-11,5	-62,0	-4,4	-40,2	-30,9	-48,3	-5,2	-33,8	-9,3	-53,6	-10,3	-41,8	-9,8	-44,9	-11,2	-57,3	-10,3	-48,7	-10,7	-57,5	-10,1	-58,0	-7,9	-43,8
350-355	H7	-10,9	-48,2	-11,2	-47,7	-24,1	-63,0	-17,7	-52,6	-16,9	-66,7	-18,2	-60,7	-11,0	-47,6	-35,8	-43,6	-9,6	-38,0	-17,9	-53,7	-16,4	-37,3	-15,4	-39,7	-17,5	-54,9	-15,5	-42,4	-17,0	-53,7	-14,8	-53,4	-11,6	-38,1
356-369	H7-H8	2,3	-2,2	-4,0	-7,1	-2,0	-9,2	-2,0	-8,1	-4,7	-9,2	3,1	-6,0	1,2	-8,4	-7,0	-10,6	-6,5	-8,7	-0,3	-6,9	-5,2	-4,3	-5,0	-4,9	-1,4	-6,5	-7,6	-4,7	0,6	0,6	-4,1	-5,9	-5,2	-6,7
357-369	H7-H8	3,7	0,9	-0,8	-6,7	-6,2	-13,5	-4,4	-4,1	-6,4	-8,7	8,9	2,0	2,9	-6,3	-3,7	-6,8	-6,1	-8,2	4,6	1,4	-0,3	2,8	1,2	2,3	3,9	0,7	-6,7	-1,9	1,2	3,1	-2,3	-2,1	-4,3	-5,4
360-369	H7-H8	0,1	0,8	-5,6	-0,7	0,3	0,2	-1,7	-1,1	-5,0	0,5	2,6	2,6	-0,1	-0,7	-3,0	1,1	-8,5	-3,3	2,0	1,7	-3,4	4,0	-3,5	3,9	-1,0	0,0	-6,9	5,7	-1,3	6,7	-1,8	6,7	-5,1	1,3
360-370	H7-H8	3,4	3,0	0,8	6,5	3,3	0,3	2,2	1,8	-1,6	5,2	-1,4	-2,5	3,1	1,8	-3,8	-1,0	-3,7	2,0	-0,6	-0,8	-4,3	-2,9	-2,6	-3,4	-0,3	-0,3	-4,0	5,2	1,2	6,4	-2,3	5,0	-2,6	3,2
374-378	H8	-0,6	-1,8	6,6	5,4	0,1	-0,2	1,4	-1,0	1,0	0,2	0,4	-3,9	-1,9	-5,4	1,1	0,7	3,1	6,2	1,5	-2,3	-1,0	-3,5	-1,0	-3,7	-0,5	-2,2	1,0	0,3	2,0	1,0	1,4	0,1	1,3	0,1
374-379	H8	1,9	1,5	1,8	4,6	0,0	-0,3	0,1	0,7	0,4	-0,6	0,0	0,9	1,2	0,7	0,6	0,6	1,6	6,6	0,1	0,5	-0,6	-0,3	-0,8	-0,3	0,1	0,5	-1,2	-0,6	-1,3	-0,9	-0,2	-0,4	-0,8	-0,9
374-394	H8-H9	-1,4	-2,8	12,2	6,7	0,1	-0,9	-1,6	-3,8	-2,5	-3,0	0,3	-0,5	-0,1	-5,0	-2,8	-1,7	11,2	9,8	0,7	0,6	-0,3	1,1	-1,0	-0,1	-0,8	-5,3	-2,1	-1,9	-2,2	-2,0	-2,3	-2,1	-2,2	-2,1
378-394	H8-H9	-2,7	-1,6	-1,7	-1,9	-3,3	-3,3	-4,0	-3,5	-3,9	-8,0	-0,7	0,1	-1,2	-2,6	-2,7	-6,2	1,7	-2,7	-0,3	1,5	-1,9	1,0	-2,2	-0,5	-1,9	0,4	-4,1	-7,1	-4,5	-6,2	-4,5	-7,3	-3,4	-6,5
380-392	H8-H9	-1,6	-3,9	3,0	-2,9	-3,2	-0,3	-2,3	-6,0	-2,3	-11,1	1,8	-3,0	-2,1	-5,5	-7,0	-8,1	6,5	0,3	2,8	3,4	-0,4	-1,5	-0,6	-4,7	0,7	1,2	-2,8	-9,6	-1,6	-7,8	-1,5	-9,4	-2,2	-10,4
379-394	H8-H9	-3,6	-3,6	6,7	2,9	-3,0	-3,0	1,6	0,6	-4,6	-10,2	-0,5	6,2	-1,9	-5,3	-2,9	-5,0	3,0	-2,0	0,1	-2,1	-2,3	7,2	-2,3	5,4	-2,3	-3,7	-5,3	-8,8	-5,6	-8,2	-4,3	-9,0	-4,6	-8,2
380-394	H8-H9	-2,3	-3,3	3,9	-2,4	-1,8	-3,4	-3,0	-6,3	-1,5	-10,2	-2,1	-6,1	-2,4	-5,5	-6,1	-5,3	8,2	3,1	-1,4	-3,0	-2,2	-3,4	-2,5	-5,5	-2,7	-4,1	-2,0	-10,2	-2,0	-10,1	-1,9	-10,5	-1,6	-8,8
376-401	H8-H9	-1,9	-2,6	-2,4	-1,2	-1,2	-2,8	-2,9	-3,9	-1,6	-5,4	-0,9	-2,6	-1,2	-3,0	-1,4	-4,9	0,1	1,8	-1,2	-2,6	-1,9	-2,0	-2,2	-3,0	-2,1	-2,8	-1,3	-4,8	-1,2	-5,1	-3,1	-6,0	-1,7	-4,4
380-401	H8-H9	-1,5	-2,7	5,5	1,7	-0,8	-3,2	-1,9	-4,9	-1,2	-7,7	-1,1	-3,8	-0,6	-3,9	-0,7	-5,6	9,1	9,0	-1,0	-2,9	-1,1	-2,6	-1,3	-3,9	-1,3	-3,2	-1,5	-7,6	-1,5	-7,5	-2,4	-9,0	-1,2	-6,4
380-403	H8-H9	-1,4	-2,5	5,4	2,4	-0,5	-3,2	-2,3	-4,7	-1,2	-7,1	-0,6	-2,7	-0,9	-3,4	-0,2	-4,7	8,9	9,1	-1,0	-2,4	-0,3	-1,9	-0,5	-2,8	-1,1	-2,5	-1,3	-6,6	-1,1	-6,5	-2,2	-8,2	-1,1	-5,7
389-394	H9	-0,9	-2,8	7,1	2,0	-2,4	-1,4	-0,7	-4,8	-2,4	-9,6	1,4	-3,5	-1,1	-4,9	-2,2	-4,0	9,2	8,4	4,2	-1,6	-1,5	3,1	-3,4	0,1	2,7	-4,2	-5,7	-4,7	-5,0	-5,5	0,9	-4,8	-2,9	-5,7
393-403	H9	-1,3	-0,2	21,5	19,8	0,8	-0,1	2,3	0,0	0,6	-2,2	1,9	-0,1	-1,4	-0,9	1,5	-0,7	19,3	24,6	0,3	0,3	0,7	2,3	1,0	0,1	2,3	0,0	-0,8	-2,5	-0,2	-1,8	0,0	-2,1	0,4	-2,8
395-401	H9	-1,4	-1,0	12,5	14,5	1,5	-0,7	-0,6	-1,8	0,8	-1,0	-0,4	-2,6	-1,6	-1,7	0,6	-1,8	13,7	21,1	-1,4	-3,1	-0,6	-3,4	-0,4	-3,9	-0,4	-2,0	1,4	-1,6	1,2	-2,2	0,9	-2,3	0,3	-1,0
394-403	H9	1,2	0,9	2,9	9,5	0,8	-0,2	-1,7	-1,8	-0,8	-3,0	-2,9	-0,9	0,0	-0,1	-2,0	-1,3	1,5	12,8	0,4	2,2	-1,6	-2,5	-1,8	-1,7	-0,5	0,9	-3,7	-4,2	-2,4	-3,5	-1,3	-2,6	-2,2	-2,5
395-402	H9	0,0	-0,9	14,7	14,3	0,6	-0,2	-0,4	-2,4	-1,6	-3,5	0,1	-4,5	-1,1	-2,1	1,0	-1,0	16,0	23,3	-0,6	-3,1	-4,6	-5,0	-1,1	-2,2	-0,9	-3,6	-0,9	-2,5	0,1	-2,5	-0,4	-1,8	-1,2	-2,0
395-403	H9	-0,6	-0,8	10,1	12,8	0,9	-1,1	-0,7	-1,5	0,3	-1,8	-0,8	-1,0	-0,7	-1,2	1,3	-0,5	10,9	17,7	-1,0	-2,1	-0,3	-1,3	-0,9	-1,8	-0,6	-1,9	-0,3	-2,0	0,1	-1,8	-0,1	-1,9	0,0	-1,7
398-403	H9	-1,9	2,5	12,0	8,3	1,7	0,0	1,3	-3,1	2,0	-0,1	0,7	-5,9	0,6	5,4	-0,6	-1,4	11,3	12,3	2,4	-3,5	1,1	-3,0	1,4	-2,6	3,1	-1,6	2,4	0,3	1,3	-0,6	3,7	-0,1	2,0	-0,2
403-418	H9-H10	-0,9	1,8	4,8	0,7	0,5	0,0	-1,4	-0,8	-1,9	-1,3	0,6	-1,6	-2,4	-1,1	-2,8	-3,0	2,8	-4,3	0,2	-1,0	-0,4	-1,3	1,7	-0,2	-0,3	0,7	-4,0	0,8	-1,7	0,2	-1,8	0,1	-0,7	0,0
404-418	H9-H10	-0,2	0,1	2,6	0,6	-1,1	-0,8	-0,5	-0,2	-1,7	-0,9	0,8	1,1	-0,4	-2,1	-3,2	-1,9	0,4	-2,6	0,2	0,5	-0,1	2,0	-0,3	1,5	-0,6	0,5	-2,9	1,5	-2,0	1,5	-0,7	0,8	-1,2	0,1
405-418	H9-H10	0,2	0,3	3,3	0,7	-1,3	-0,9	1,2	-0,2	-1,8	-1,5	3,7	7,1	0,6	-1,7	-4,0	-0,6	0,4	-1,7	2,8	5,4	3,2	7,1	2,6	3,0	2,0	5,4	-3,0	0,7	-4,3	-0,5	-2,7	-0,8	-1,7	-0,1
403-421	H9-H10	-0,7	-4,8	3,4	-1,4	-1,1	-7,5	-1,6	-5,3	0,7	-4,7	1,2	-4,9	0,1	-7,1	-3,2	-5,1	3,0	-0,6	-0,7	-3,2	1,3	-1,2	0,6	-2,7	-2,5	-6,1	-0,1	-0,7	0,9	-2,5	1,5	-1,4	1,7	0,4
406-418	H9-H10	0,4	0,7	0,9	-1,9	-1,4	-0,3	-0,8	-0,8	-2,5	-1,0	2,7	3,6	0,5	-0,8	-5,5	-4,2	-1,8	-5,5	1,4	2,8	0,7	4,7	0,5	4,1	0,4	2,8	-3,7	1,5	-2,6	1,7	-2,0	0,1	-1,2	0,7
404-422	H9-H10	-0,6	-4,0	3,2	1,1	-1,0	-3,8	-1,5	-4,6	-3,7	-6,4	-1,1	-2,9	-0,5	-5,7	-3,3	-5,2	2,3	1,8	0,0	1,1	-1,7	-0,7	-1,3	-2,8	-2,0	-2,5	-2,6	-4,1	-2,4	-3,8	-2,1	-5,5	-3,3	-4,4
405-421	H9-H10	-0,6	-3,0	3,3	-2,2	0,0	-5,4	0,2	-4,6	-3,1	-6,9	-0,7	1,2	-0,6	-5,3	-5,2	-6,2	1,9	-0,5	-2,1	2,6	-2,1	0,3	-2,1	-0,4	-2,4	-1,6	-4,0	-2,7	-4,1	-3,4	-2,4	-4,0	-3,2	-4,4

406-421	H9-H10	-0,8	-3,5	1,6	-0,3	-1,7	-5,9	-1,7	-1,1	-3,2	-7,2	-2,3	-3,1	0,1	-7,2	-3,7	-7,7	2,1	-0,2	-0,7	-2,1	-2,2	-2,0	-3,4	-3,9	-1,0	-5,3	-4,0	-5,2	-3,3	-5,6	-4,5	-7,1	-2,2	-5,7
407-421	H9-H10	-4,7	-8,2	3,6	-2,2	-1,2	-2,8	-1,1	-6,5	-3,6	-8,0	1,2	-2,3	-11,9	-9,1	-5,4	-8,9	-0,2	-2,1	0,3	-2,3	-0,4	-2,8	-1,1	-3,3	-0,4	-4,3	-4,1	-3,3	-2,8	-2,8	-1,9	-4,0	-1,7	-4,4
423-428	H10/11	-2,3	-0,3	-3,5	7,9	-1,2	0,3	-3,0	-0,9	-0,3	-4,5	-2,9	-4,8	-0,3	-4,0	-7,5	-15,7	-8,7	-4,5	-2,1	-5,7	-2,6	-0,8	-3,0	-7,3	-4,8	-2,4	-4,7	-7,1	-3,3	-5,8	-0,9	-5,4	-0,3	-5,2
424-428	H10/11	-3,7	-2,3	-4,2	1,0	-3,8	-7,8	-4,6	-4,3	-2,1	-7,7	-4,5	-9,3	-0,9	-5,7	-8,5	-13,5	-10,8	-10,0	-2,8	-5,2	-5,3	-5,0	-4,9	-8,8	-7,2	-5,7	-5,7	-7,7	-5,5	-8,1	-0,6	-8,1	-3,1	-6,6
426-434	H10/11	-22,5	-21,9	-16,8	-18,7	-22,3	-13,1	-25,4	-21,3	-27,5	-29,5	-27,0	-24,1	-21,9	-21,9	-35,3	-23,0	-11,4	-8,4	1,4	2,2	-22,2	-15,1	-21,2	-17,2	-28,6	-18,2	-22,5	-15,5	-27,0	-22,2	-21,1	-21,8	-15,5	-12,3
426-440	H10/11	-44,1	-28,4	-37,0	-27,7	-33,4	-8,0	-46,6	-22,9	-50,6	-44,1	-49,0	-31,8	-43,5	-23,9	-40,3	-25,1	-15,4	-10,0	2,4	2,9	-38,0	-10,8	-32,8	-11,3	-45,1	-17,9	-31,5	-12,2	-44,0	-21,8	-40,7	-22,4	-24,4	-7,7
426-441	H10/11	-46,2	-29,4	-37,0	-27,6	-35,2	-7,5	-47,1	-22,0	-53,2	-45,3	-49,8	-28,5	-44,5	-23,0	-41,1	-23,7	-14,7	-11,3	2,8	4,0	-38,4	-8,9	-33,2	-9,7	-46,0	-16,7	-32,5	-10,5	-46,0	-20,2	-43,1	-22,4	-25,2	-7,4
428-440	H10/11	-52,4	-28,0	-40,5	-28,0	-39,3	-3,4	-59,2	-20,6	-58,9	-48,2	-60,3	-33,7	-49,2	-23,6	-48,3	-27,5	-16,9	-11,7	5,8	8,4	-35,6	-6,9	-35,7	-7,2	-50,3	-13,2	-36,1	-8,6	-50,5	-18,6	-48,4	-22,2	-27,9	-5,6
429-440	H10/11	-60,6	-30,6	-45,6	-32,7	-42,1	-3,5	-56,3	-23,5	-64,4	-51,8	-60,2	-33,0	-58,1	-27,4	-50,7	-26,2	-15,5	-10,7	4,3	5,8	-46,1	-4,6	-38,7	-4,2	-52,3	-15,9	-36,8	-6,9	-53,9	-18,4	-52,6	-21,1	-29,7	-5,4
429-441	H10/11	-63,6	-31,8	-44,8	-32,1	-44,1	-2,7	-57,4	-22,2	-66,9	-52,2	-60,9	-29,0	-59,6	-25,5	-51,4	-26,1	-15,7	-12,3	4,7	6,6	-46,6	-3,0	-39,6	-2,5	-53,7	-15,2	-36,4	-6,0	-55,4	-17,6	-54,5	-20,4	-29,9	-4,9
441-453	H10/11-H12	-16,0	-1,5	-12,8	-2,7	-5,0	1,0	-16,1	-1,6	-21,7	-3,4	-17,4	-1,3	-13,6	-0,5	-15,5	-5,4	-5,7	-2,6	1,9	-1,2	-12,4	-0,1	-8,5	0,4	-14,9	-2,2	-8,1	1,9	-16,1	1,7	-15,5	1,1	-4,5	3,5
441-454	H10/11-H12	-20,6	-1,1	-18,1	-2,9	-5,3	0,9	-22,1	-1,0	-29,0	-5,5	-22,0	1,1	-16,5	-0,9	-20,4	-5,1	-7,2	-3,9	2,9	0,3	-15,7	1,5	-10,6	2,0	-18,3	0,8	-9,9	1,7	-20,6	1,9	-20,4	0,7	-5,5	3,1
442-453	H10/11-H12	-14,5	-1,3	-11,3	-3,0	-5,2	0,9	-14,6	-0,7	-18,6	-4,3	-15,3	-2,7	-12,5	-1,6	-13,9	-3,7	-6,7	-2,6	1,3	-0,7	-11,8	0,1	-9,6	0,9	-13,0	-1,6	-7,6	0,9	-14,7	0,6	-13,4	0,9	-4,1	2,8
441-456	H10/11-H12	-31,6	-2,0	-15,3	-0,3	-12,5	2,3	-26,0	1,7	-42,8	-18,8	-29,9	5,5	-26,1	-3,5	-28,7	-0,9	-7,8	-1,8	3,5	3,8	-22,2	6,6	-17,6	6,7	-26,6	-12,6	-20,9	5,2	-29,7	2,6	-32,2	-5,4	-12,0	1,9
441-457	H10/11-H12	-33,8	-5,6	-22,5	-2,7	-14,9	1,4	-32,4	-3,9	-43,9	-14,5	-34,3	-1,6	-28,9	-5,0	-34,9	-6,7	-4,9	-3,0	1,8	0,5	-29,5	2,7	-20,8	3,5	-33,6	-6,3	-17,7	5,2	-33,1	0,4	-30,7	0,0	-13,3	4,4
454-461	H12	-31,3	-6,4	-23,5	-4,7	-23,3	1,0	-33,8	-5,7	-39,1	-16,8	-33,9	-6,4	-31,4	-4,6	-31,5	-9,6	-5,2	-1,2	3,2	0,6	-30,6	-2,8	-25,2	-0,5	-33,6	-4,7	-24,0	-1,3	-36,9	-6,2	-34,6	-7,6	-17,1	2,1
455-460	H12	-32,3	-7,7	-24,1	-3,8	-20,1	6,8	-33,8	-4,6	-40,1	-17,1	-36,4	-6,2	-32,7	-7,0	-31,4	-8,8	-7,6	-1,2	2,3	-2,2	-33,6	-3,7	-28,1	-1,4	-36,5	-10,0	-31,5	-11,8	-39,6	-16,6	-36,1	-7,7	-17,4	1,2
455-461	H12	-22,0	-2,6	-16,0	-8,3	-15,3	-0,9	-22,9	-12,2	-31,9	-13,7	-17,7	-3,3	-24,2	-5,3	-23,5	-6,9	-3,3	-6,6	16,2	7,5	-15,0	-1,9	-7,9	0,3	-13,2	-0,9	-16,0	1,0	-29,4	-3,2	-25,1	-4,5	-5,9	0,0
457-461	H12	1,5	0,7	-0,2	1,9	1,4	-1,1	2,0	4,4	5,7	6,3	-3,2	0,1	0,4	0,8	-2,2	0,8	-1,0	4,5	-0,4	5,7	0,2	4,2	1,5	8,0	-0,3	7,7	0,4	4,1	-1,0	5,2	3,3	6,2	7,4	9,5

Supplementary Table 2: Crystallographic data and refinement.

Related to Figure 2

	LXRβ-AZ3	LXRβ-AZ6	LXRβ-AZ8	LXRβ-WAY-254011
Data collection				
Space group	$P3_2$ 2	$P2_1$	$P3_22_1$	$I4_122$
Cell dimensions	a=125.1 Å, b=125.1 Å, c=125.1 Å	a=55.4 Å, b=109.4 Å, c=89.0 Å, β=90.9°	a=58.7 Å, b=58.7Å, c=148.0 Å	a= 135.3 Å, b=135.3 Å, c=69.73 Å
Resolution (Å)	20.0 – 3.0 (3.23-3.0)[1]	89.1 – 1.9 (2.0-1.9)[1]	24.0– 1.6 (1.69-1.60)[1]	95.8– 2.0 (2.11-2.0)[1]
R_{merge}	0.081 (0.48)	0.093 (0.59)	0.055 (0.45)	0.093 (0.62)
$<I / sI>$	16.6 (4.9)	8.3 (1.7)	8.3 (1.8)	5.5 (1.3)
Completeness (%)	99.8 (100)	92.7 (86.8)	96.2 (81.6)	99.8 (100)
Redundancy	- (-)	2.0 (1.9)	4.9 (3.1)	9.2 (9.5)
Refinement				
Measured / unique refl.	- / 30616	150698 / 80516	475154 /38673	203885 /22128
R_{work} / R_{free}	0. 241/ 0.271	0.203 / 0.259	0.240 / 0.251	0.222 / 0.254
No. atoms				
Protein	5742	7695	1924	1933
Water	18	-	338	30
Ligand	96	245		78
Average *B*-factors				
Protein ($Å^2$)	74,5	22,5	42,2	35,8
Water ($Å^2$)	53,6	-	35,2	46,5
Ligand ($Å^2$)	80,5	29,8	20,8	28,2
R.m.s deviations				
Bond lengths (Å)	0,007	0,011	-	0,014
Bond angles (°)	0,98	1,4	-	1,8

[1] Values in parentheses refer to highest-resolution shell

Supplementary Table 3: Peptides with highest contribution to the discrimination between lipogenic and non-lipogenic compounds.
Related to Figure 3
Significance selection criteria included correlation coefficients (|p(corr)| > 0.7) and the coefficients of contribution to the model separation between TG classes (p > 0.05).

Peptide	LXR isoform & Labelling Time	Amino Acid Sequence	Secondary Structure	OPLS-DA		Lipogenic (n = 5) vs non-lipogenic (n = 7): unpaired t-test		
				p	p(corr)	P-value	P-value summary	t, df*
TG OPLS-DA: LXRα and LXRβ HDX-MS								
270-275	LXRα/30 sec	DFAKQL	H3	0,0744295	0,697956	0,0189	*	t=2,681, df=13
412-426	LXRα/30 sec	VSLRTLSSVHSEQVF	H10/11	0,092981	0,715587	0,0344	*	t=2,362, df=13
412-427	LXRα/30 sec	VSLRTLSSVHSEQVFA	H10/11	0,0971066	0,721315	0,0304	*	t=2,429, df=13
414-426	LXRα/30 sec	LRTLSSVHSEQVF	H10/11	0,104203	0,734507	0,027	*	t=2,492, df=13
414-427	LXRα/30 sec	LRTLSSVHSEQVFA	H10/11	0,116561	0,74986	0,0204	*	t=2,639, df=13
415-426	LXRα/30 sec	RTLSSVHSEQVF	H10/11	0,116667	0,74078	0,0279	*	t=2,474, df=13
415-427	LXRα/30 sec	RTLSSVHSEQVFA	H10/11	0,124186	0,748133	0,0199	*	t=2,652, df=13
428-441	LXRα/30 sec	LRLQDKKLPPLLSE	H10/11-H12	0,0510492	0,773292	0,0132	*	t=2,869, df=13
427-443	LXRα/30 sec	ALRLQDKKLPPLLSEIW	H10/11-H12	0,0827411	0,775786	0,013	*	t=2,875, df=13
428-442	LXRα/30 sec	LRLQDKKLPPLLSEI	H10/11-H12	0,0652641	0,741346	0,0354	*	t=2,347, df=13
428-443	LXRα/30 sec	LRLQDKKLPPLLSEIW	H10/11-H12	0,0834724	0,711789	0,0699	ns	t=1,975, df=13
440-447	LXRα/30 sec	SEIWDVHE	H12	0,0934413	0,825616	0,0084	**	t=3,104, df=13
441-446	LXRα/30 sec	EIWDVH	H12	0,116567	0,920834	0,0005	***	t=4,654, df=13
214-219	LXRα/600 sec	IEKLVA	H1	0,11695	0,825104	0,0011	**	t=4,160, df=13
290-296	LXRα/600 sec	LKTSAIE	H5	0,0811372	0,743081	0,0061	**	t=3,270, df=13
326-331	LXRα/600 sec	FAKAGL	H6	0,0735871	0,697892	0,0024	**	t=3,762, df=13
327-331	LXRα/600 sec	AKAGL	H6	0,0850626	0,759729	0,0009	***	t=4,300, df=13
333-341	LXRα/600 sec	VEFINPIFE	H7	0,116319	0,727585	0,006	**	t=3,275, df=13
333-345	LXRβ/30 sec	FTYSKDDFHRAGL	βsheets, H6	0,0952277	0,887247	<0,0001	****	t=5,865, df=13
333-346	LXRβ/30 sec	FTYSKDDFHRAGLQ	βsheets, H6	0,0902183	0,870471	<0,0001	****	t=6,063, df=13
341-346	LXRβ/30 sec	HRAGLQ	H6-H7	0,143145	0,818952	0,001	**	t=4,215, df=13
341-347	LXRβ/30 sec	HRAGLQV	H6-H7	0,107901	0,837063	0,0028	**	t=3,677, df=13
341-348	LXRβ/30 sec	HRAGLQVE	H6-H7	0,100804	0,791383	0,0076	**	t=3,156, df=13
341-349	LXRβ/30 sec	HRAGLQVEF	H6-H7	0,108573	0,833864	0,0057	**	t=3,308, df=13
349-354	LXRβ/30 sec	FINPIF	H7	0,0599492	0,705961	0,0102	*	t=3,004, df=13
349-355	LXRβ/30 sec	FINPIFE	H7	0,0547438	0,70169	0,0104	*	t=2,990, df=13
429-440	LXRβ/30 sec	RTLSSVHSEQVF	H10/11	0,154339	0,717002	0,0236	*	t=2,562, df=13
429-441	LXRβ/30 sec	RTLSSVHSEQVFA	H10/11	0,161647	0,724354	0,0234	*	t=2,567, df=13
441-453	LXRβ/30 sec	ALRLQDKKLPPLL	H10/11-H12	0,0501846	0,713708	0,0661	ns	t=2,006, df=13
441-454	LXRβ/30 sec	ALRLQDKKLPPLLS	H10/11-H12	0,0680182	0,713626	0,057	ns	t=2,088, df=13
441-457	LXRβ/30 sec	ALRLQDKKLPPLLSEIW	H10/11-H12	0,104948	0,723603	0,0853	ns	t=1,863, df=13
304-310	LXRβ/600 sec	LKASTIE	H5	0,0945143	0,698917	0,0563	ns	t=2,095, df=13
305-310	LXRβ/600 sec	KASTIE	H5	0,0936218	0,7409	0,0259	*	t=2,515, df=13
333-345	LXRβ/600 sec	FTYSKDDFHRAGL	βsheets, H6	0,131977	0,733755	0,0023	**	t=3,774, df=13
333-346	LXRβ/600 sec	FTYSKDDFHRAGLQ	βsheets, H6	0,14834	0,736355	0,003	**	t=3,646, df=13
341-346	LXRβ/600 sec	HRAGLQ	H6-H7	0,176995	0,796951	0,0022	**	t=3,798, df=13
341-347	LXRβ/600 sec	HRAGLQV	H6-H7	0,130883	0,756441	0,0055	**	t=3,324, df=13
341-348	LXRβ/600 sec	HRAGLQVE	H6-H7	0,108676	0,738019	0,0037	**	t=3,531, df=13
341-349	LXRβ/600 sec	HRAGLQVEF	H6-H7	0,131281	0,768548	0,0029	**	t=3,656, df=13
348-355	LXRβ/600 sec	EFINPIFE	H7	0,10038	0,718621	0,0069	**	t=3,208, df=13
349-354	LXRβ/600 sec	FINPIF	H7	0,0989918	0,79585	0,0046	**	t=3,419, df=13
349-355	LXRβ/600 sec	FINPIFE	H7	0,097601	0,769606	0,0056	**	t=3,317, df=13
426-434	LXRβ/600 sec	VSLRTLSSV	H10/11	0,0628284	0,712973	0,0291	*	t=2,452, df=13
426-440	LXRβ/600 sec	VSLRTLSSVHSEQVF	H10/11	0,107806	0,792896	0,0071	**	t=3,189, df=13
426-441	LXRβ/600 sec	VSLRTLSSVHSEQVFA	H10/11	0,109961	0,7865	0,0067	**	t=3,220, df=13
428-440	LXRβ/600 sec	LRTLSSVHSEQVF	H10/11	0,129789	0,785936	0,006	**	t=3,273, df=13
429-440	LXRβ/600 sec	RTLSSVHSEQVF	H10/11	0,143264	0,803832	0,0034	**	t=3,578, df=13
429-441	LXRβ/600 sec	RTLSSVHSEQVFA	H10/11	0,141402	0,785316	0,0044	**	t=3,442, df=13

* t: t-values, df: degrees of freedom

Supplementary Table 4: Differences in deuteration levels (% of maximal deuteration) of LXR LBDs in presence of the SRC1 coactivator peptide and different ligands.

Related to Figure 3

Deuterium uptake values for all peptides were corrected to back exchange using the fully deuterated controls. Missing peptides are shown in dark grey. HDX-MS measurements were performed in triplicates and the average values are shown. Color scheme is identical to Figure 2.

		T0-091317		WAY-254011		F1		AZ876		AZ1		AZ2		AZ3		AZ4		GW3965		BMS-852927		AZ6		AZ7		LXR-623		AZ8		AZ9		AZ5		24,25EC	
		ΔHDX (apo - ligand), %																																	
Peptide	Secondary Structure	30 sec	600 sec	30 sec	600 sec	30 sec	600 sec	30 sec	600 sec	30 sec	600 sec	30 sec	600 sec	30 sec	600 sec	30 sec	600 sec	30 sec	600 sec	30 sec	600 sec	30 sec	600 sec	30 sec	600 sec	30 sec	600 sec	30 sec	600 sec	30 sec	600 sec	30 sec	600 sec	30 sec	600 sec
		LXRα																																	
200-210	H1	1,7	-5,9	-0,9	-8,9	-2,0	-2,3	-1,2	1,5	-4,8	-7,6	-2,3	-8,4	-1,0	-2,5	2,5	-8,1	-0,5	-8,5	3,7	-8,6	2,0	-0,5		-8,3	-2,0	-7,2	1,4	1,2	9,9	-5,6	-1,1	-9,2	-3,5	-4,6
200-213	H1	6,4	-0,5	2,6	-1,8	6,0	2,1	3,1	-0,3	2,9	-2,7	-1,7	-5,4	3,8	1,7	3,1	-0,2	1,5	-2,0	7,3	-0,4	5,4	3,1	4,5	-0,2	-1,8	-3,7	3,6	-1,9	3,3	0,8	-2,4	-5,2	4,4	-0,4
204-213	H1	6,9	-1,3	-0,6	-1,9	3,4	-0,5	-1,5	-1,8	-1,0	-2,3	-4,3	-5,7	-1,2	-1,1	-0,2	0,9	-2,0	-6,0	6,2	-2,0	4,5	3,2	0,6	0,6	-5,0	-3,1	-0,9	-0,3	-0,8	0,5	-3,5	-5,4	0,5	-3,6
208-213	H1	5,8	1,7	3,8	-6,3	3,5	2,8	-2,5	-14,9	-3,5	-5,9	5,1	3,7	3,0	2,8	2,4	-1,0	0,2	-1,4	5,2	1,1	4,6	4,2	2,8	-3,1	0,5	-11,5	-1,4	-7,0	1,0	-2,6	7,7	6,5	7,6	2,1
214-219	H1	-19,0	-28,6	-17,8	-49,6	-15,5	-39,2	-19,7	-47,1	-20,0	-48,3	-17,1	-41,9	-19,8	-39,7	-19,4	-47,0	-18,1	-32,4	-20,9	-29,4	-23,1	-40,1	-12,8	-41,8	-18,2	-36,9	-10,4	-20,3	-17,5	-42,7	-14,6	-29,3	-20,2	-36,4
214-233	H1	-17,7	-13,2	-22,1	-23,0	-18,8	-17,2	-22,0	-20,8	-18,0	-18,0	-22,5	-20,5	-17,0	-13,5	-18,6	-17,7	-15,6	-15,0	-15,1	-12,5	-22,0	-13,5	-17,3	-15,5	-17,8	-16,6	-13,9	-12,6	-16,9	-15,9	-18,6	-16,0	-19,4	-17,7
220-233	H1	-9,1	-8,1	-12,3	-8,4	-9,1	-3,5	-11,6	-7,4	-10,7	-7,0	-13,9	-11,7	-9,9	-4,6	-9,2	-4,3	-9,0	-8,1	-7,7	-7,5	-10,7	-2,2	-10,9	-5,0	-10,2	-8,1	-9,9	-6,0	-10,1	-5,8	-11,7	-9,9	-9,1	-4,8
234-240	loop H1-H3	0,2	1,2	-6,3	0,3	-3,5	2,4	-4,3	-0,6	-2,2	-3,3	-5,7	-5,3	-3,3	2,3	-2,5	-1,2	-4,2	-2,7	1,2	0,4	-2,0	4,3	-1,9	0,9	-3,6	-1,5	-0,3	-0,2	-0,8	1,5	-3,6	-3,5	-6,8	-0,3
234-248	loop H1-H3	-3,7	-4,9	-9,1	-6,0	-1,7	8,9	-22,6	-5,3	-4,5	-5,7	-8,2	-4,0	0,4	10,7	-4,8	-4,5	5,7	8,4	-2,3	-1,7	-5,9	1,7	-3,3	-1,8	-5,6	-6,7	-7,9	-7,2	-7,0	-0,7	-3,9	-1,8	-3,8	10,4
234-257	loop H1-H3, H3	-9,9	-3,9	-16,7	-4,4	-11,5	-1,1	-23,1	-4,8	-9,3	-2,2	-15,4	-6,4	-11,9	-1,1	-9,2	0,2	-9,0	-2,2	-3,7	-0,7	-10,6	3,6	-7,8	0,4	-9,0	-3,8	-8,8	-2,2	-9,3	-0,9	-8,8	-4,6	-17,0	-1,0
239-257	loop H1-H3, H3	-13,0	-4,5	-18,5	-7,2	-11,3	-0,6	-21,9	-7,1	-11,3	-4,5	-19,3	-7,8	-11,5	-0,3	-11,0	-1,8	-8,8	-0,8	-4,4	-0,8	-11,4	4,6	-8,4	-1,9	-10,1	-5,7	-8,1	-3,9	-9,7	-2,8	-13,7	-6,2	-15,9	1,0
241-257	loop H1-H3, H3	-5,6	-6,9	0,0	4,8	-5,8	-2,5	2,0	5,7	2,4	5,5	-9,1	-7,3	-6,1	-3,3	4,4	7,7	-8,1	-7,4	-3,7	-5,4	-5,9	-1,9	1,8	7,2	2,3	5,1	4,5	8,3	2,9	8,0	-10,8	-6,9	-8,1	-3,6
260-265	H3	-51,0	-73,3	-44,9	-78,8	-40,6	-75,4	-48,3	-84,2	-47,4	-82,2	-33,9	-80,8	-40,6	-72,7	-47,5	-79,6	-38,5	-68,6	-52,2	-72,2	-49,1	-55,6		-80,6	-41,5	-53,9		-56,0	-43,7	-63,6	-20,3	-36,6	-35,6	-63,5
261-267	H3	-49,9	-67,8	-44,7	-78,1	-41,0	-69,7	-46,3	-81,9	-45,4	-78,6	-41,6	-64,5	-39,7	-64,7	-43,0	-70,5	-41,2	-64,3	-51,7	-69,6	-47,1	-54,4	-41,5	-63,7	-38,2	-54,8	-40,2	-54,2	-39,4	-56,9	-27,7	-27,1	-35,9	-51,8
267-271	H3	2,7	-14,7	-3,4	-18,8	-3,3	-16,2	-4,7	-19,0	-4,8	-21,4	0,1	-13,3	-3,7	-16,1	-4,2	-18,8	-3,1	-13,0	2,7	-14,9	2,3	-12,5	-3,5	-19,3	-2,1	-15,3	-2,8	-18,7	-3,3	-17,4	1,5	-10,2	-2,8	-10,2
271-278	H3	-1,1	-10,2	-1,8	-9,0	-0,5	-8,9	-1,5	-9,0	-1,8	-9,6	-2,6	-10,2	-1,2	-8,4	-1,6	-8,2	-0,4	-6,9	-0,9	-9,7	-0,5	-6,8	-2,0	-8,1	-1,3	-8,0	-1,7	-9,0	-1,2	-7,3	-2,2	-8,4	-1,0	-5,7
271-281	H3-H4	-1,1	-9,1	-3,9	-7,9	0,0	-7,6	-4,9	-8,8	-4,4	-9,2	-5,0	-11,5	-0,8	-7,8	-5,1	-7,7	-0,9	-7,0	-1,3	-8,1	-3,7	-6,3	-6,1	-8,1	-2,1	-7,0	-5,2	-7,4	-3,2	-5,8	-4,4	-7,0	-0,8	-6,3
272-278	H3-H4	0,4	-9,0	-0,5	-8,3	1,6	-7,3	1,7	-5,5	0,2	-8,7	-1,6	-10,2	0,5	-7,2	0,4	-7,5	-0,1	-7,3	0,3	-8,2	0,3	-6,0	0,8	-5,4	0,2	-6,5	0,8	-6,3	0,2	-3,6	-1,0	-8,0	0,3	-5,4
272-281	H3-H4	-1,5	-9,5	-6,5	-11,1	-2,4	-9,4	-6,3	-11,2	-6,0	-11,5	-7,7	-13,5	-3,1	-8,8	-6,7	-9,4	-3,5	-7,0	-2,0	-8,7	-4,1	-6,5	-7,2	-10,2	-4,8	-10,5	-6,5	-9,8	-5,1	-9,1	-7,2	-10,6	-3,5	-6,7
273-278	H3-H4	3,4	-15,7	-1,5	-16,9	2,4	-7,0	-6,7	-15,8	-8,0	-20,3	4,1	-8,3	4,2	-4,4	-5,4	-15,6	1,7	-1,8	-1,1	-18,3	0,3	-12,5	-6,7	-18,0	-1,3	-15,3	1,3	-16,1	-2,9	-11,6	4,4	-5,1	1,1	-1,0
279-286	H4-H5	4,7	-6,8	2,9	-3,7	3,3	0,7	7,1	4,6	-6,3	-6,3	-2,1	-10,8	4,5	0,5	1,6	2,3	0,7	2,8	4,5	-5,3	-0,1	-1,6		-7,8	6,3	-4,9	-6,7	-0,6	5,1	-4,9	-1,8	-7,2	0,1	-2,4
280-290	H4-H5	-4,0	-10,7	-2,8	-6,9	-1,1	-7,9	-3,9	-6,5	-3,1	-7,5	-6,7	-20,0	-2,3	-8,2	-3,1	-5,7	-2,1	-8,2	-3,7	-10,9	-5,2	-8,5	-5,1	-5,3	-3,2	-6,1	-4,0	-6,6	-3,4	-5,7	-6,7	-18,2	-2,2	-7,9
282-289	H4-H5	0,4	-7,8	-0,7	-7,4	0,0	-6,8	-1,1	-6,7	-1,3	-7,1	-2,5	-9,7	-0,8	-7,1	-1,2	-6,1	-0,9	-6,6	-0,1	-8,0	-0,2	-5,9	-1,4	-6,5	-0,5	-6,8	-0,4	-6,8	0,0	-6,3	-2,3	-7,4	-1,7	-5,0
282-290	H4-H5	0,4	-9,0	0,7	-7,4	0,2	-6,6	2,5	-5,4	-0,3	-7,1	-2,3	-10,4	0,3	-6,2	0,7	-6,8	-0,3	-4,9	-0,4	-9,1	-1,0	-7,9	1,2	-5,3	1,2	-6,7	3,2	-4,6	2,0	-5,5	-1,5	-8,2	0,5	-3,7
285-290	H5	3,3	-4,5	0,3	-5,2	-3,2	-6,2	-2,5	-5,2	-3,2	-7,7	-1,0	-3,2	-3,3	-6,3	-1,4	-8,9	-2,0	-3,1	2,9	-3,8	3,2	-3,1	-1,8	-9,2	0,6	-5,4	-3,4	-5,2	-2,8	-4,5	0,1	0,6	-3,3	-5,1
290-294	H5	-32,3	-51,5	-27,2	-61,9	-27,2	-51,3	-29,8	-64,0	-26,6	-62,2	-25,5	-58,3	-29,0	-55,7	-26,7	-62,8	-29,1	-53,4	-28,1	-32,0	-32,1	-53,6	-28,0	-60,3	-23,9	-53,5	-30,3	-60,1	-25,4	-58,2	-15,9	-45,0	-27,5	-48,2
295-299	H5-βsheets	2,3	-6,8	-1,1	-8,8	0,4	-7,7	-2,7	-8,6	-3,6	-10,3	-1,8	-8,0	-2,6	-9,1	-3,4	-9,4	-3,4	-9,6	2,1	-7,2	3,6	-4,9	-3,8	-10,4	-1,1	-7,2	-2,2	-9,3	-2,2	-8,5	-1,3	-6,5	-2,5	-7,1
299-311	H5-βsheets	-19,4	-21,6	-18,5	-32,9	-18,0	-33,8	-18,2	-26,4	-17,4	-28,1	-19,4	-23,5	-16,2	-20,1	-16,4	-28,0	-17,4	-30,9	-21,6	-31,7	-20,7	-24,4	-17,0	-23,5	-4,3	-7,3	-16,7	-17,6	-15,1	-16,5	-16,3	-20,7	-16,0	-22,2
299-314	H5-βsheets	-29,3	-27,5	-30,3	-44,1	-29,0	-40,6	-30,9	-34,5	-29,4	-40,6	-27,2	-34,4	-26,3	-32,5	-27,7	-36,6	-26,7	-37,9	-33,4	-41,6	-30,6	-32,5	-28,5	-30,6	-18,3	-15,4	-26,4	-25,9	-25,3	-20,5	-20,1	-21,8	-23,3	-28,0
299-315	H5-βsheets	-29,4	-22,7	-34,3	-46,6	-29,5	-38,9	-32,5	-32,9	-33,4	-42,6	-36,3	-37,9	-29,8	-34,3	-31,6	-38,8	-31,0	-39,9	-36,7	-40,3	-32,8	-30,0	-30,7	-30,7	-19,9	-15,6	-29,2	-22,7	-26,4	-19,6	-27,3	-23,9	-26,6	-28,9
300-311	H5-βsheets	-19,7	-19,8	-18,5	-33,6	-18,6	-34,5	-19,5	-29,7	-16,6	-27,4	-19,3	-22,2	-16,7	-20,0	-15,3	-27,1	-17,7	-30,5	-21,7	-31,2	-20,7	-22,9	-17,5	-22,8	-2,8	-5,2	-18,0	-17,5	-15,0	-14,8	-15,4	-18,6	-16,2	-21,2
300-312	H5-βsheets	-26,1	-24,4	-25,4	-39,5	-24,6	-39,9	-28,4	-35,4	-23,9	-33,9	-25,0	-29,6	-22,5	-27,1	-21,9	-32,0	-23,5	-35,8	-28,4	-36,0	-27,1	-26,2	-24,3	-26,8	-11,6	-11,2	-24,4	-20,5	-21,2	-19,0	-19,3	-20,2	-21,3	-26,2
300-315	H5-βsheets	-30,6	-21,1	-37,3	-48,5	-31,3	-39,5	-35,2	-33,8	-35,4	-44,0	-39,3	-39,4	-31,9	-34,2	-33,3	-40,1	-31,9	-39,8	-39,3	-41,0	-35,6	-28,9	-32,2	-30,9	-22,4	-16,6	-30,9	-22,0	-28,3	-19,7	-29,9	-24,2	-28,2	-28,4
312-316	βsheets	-24,8	-3,2	-69,1	-54,1	-37,4	-22,9	-38,5	-14,6	-62,8	-42,2	-52,6	-30,8	-51,0	-34,3	-62,5	-36,0	-52,1	-36,8	-45,7	-31,7	-56,3	-20,9	-56,5	-22,8	-32,4	-16,7	-33,2	-12,6	-31,2	-8,6	-31,4	-14,3	-45,1	-24,9
316-326	βsheets-H6	-29,5	-30,6	-37,6	-32,0	-33,6	-32,8	-30,9	-33,2	-30,9	-31,1	-31,9	-31,8	-28,0	-30,2	-31,6	-24,6	-32,3	-31,1	-34,4	-32,2	-33,8	-21,4	-28,7	-23,6	-27,3	-25,0	-25,2	-22,1	-25,5	-22,8	-24,5	-21,1	-27,3	-26,2
319-324	βsheets-H6	-11,6	-11,8	-15,5	-12,0	-9,3	-12,2	-12,8	-7,2	-10,9	-11,6	-6,6	-7,3	-8,3	-9,5	-11,3	-7,5	-9,1	-11,3	-9,4	-10,5	-10,3	-6,9	-8,8	-2,8	-12,8	-5,6	-1,7	-0,1	-7,6	-2,0	-5,7	1,4	-11,8	-7,5
319-326	βsheets-H6	-39,3	-38,7	-42,2	-45,2	-35,3	-43,8	-42,6	-47,4	-39,0	-45,0	-39,7	-43,0	-34,5	-38,8	-37,5	-38,7	-35,2	-39,6	-40,2	-39,5	-38,1	-28,3	-36,1	-36,5	-36,8	-34,1	-33,2	-32,5	-34,6	-34,0	-30,2	-21,4	-35,0	-32,1
319-331	βsheets-H6	-49,5	-39,1	-41,8	-36,3	-47,6	-47,9	-55,0	-55,3	-50,5	-48,2	-47,5	-44,0	-42,5	-39,2	-47,7	-41,3	-38,7	-30,6	-38,4	-17,9	-46,6	-28,6	-40,0	-30,1	-35,1	-26,5	-41,1	-26,7	-37,9	-27,8	-32,8	-16,4	-41,4	-34,2
320-331	βsheets-H6	-57,9	-45,4	-46,5	-41,2	-53,1	-54,7	-61,6	-64,5	-56,3	-55,5	-41,5	-45,9	-46,6	-45,4	-53,0	-47,5	-40,6	-34,9	-42,6	-18,4	-54,4	-31,7	-47,2	-36,1	-40,4	-30,4	-48,0	-30,4	-43,8	-32,0	-25,0	-20,9	-46,3	-38,5
321-331	βsheets-H6	-71,0	-56,1	-46,2	-42,9	-61,6	-59,8	-64,2	-66,9	-59,4	-59,7	-56,0	-53,6	-52,4	-50,8	-53,5	-50,1	-44,3	-36,8	-49,1	-22,0	-62,7	-37,2	-48,5	-39,2	-38,0	-31,2	-47,1	-30,7	-43,7	-31,6	-37,8	-25,3	-51,5	-42,8
326-331	H6	-58,0	-30,7	-37,0	-25,9	-62,0	-43,8	-69,2	-55,9	-62,7	-44,1	-52,4	-32,4	-49,0	-32,6	-57,2	-39,8	-37,3	-11,6	-28,0	11,0	-55,7	-18,8	-42,5	-16,7	-33,0	-15,7	-48,0	-14,9	-40,3	-22,3	-32,6	-6,1	-48,7	-33,1
327-331	H6	-53,2	-36,6	-37,5	-31,2	-59,3	-47,9	-67,0	-54,2	-60,3	-48,6	-51,5	-39,6	-47,4	-40,7	-54,4	-41,9	-35,4	-18,8	-33,2	6,3	-48,3	-25,5	-39,4	-31,4	-33,8	-26,9	-48,9	-28,5	-36,2	-27,4	-31,1	-9,3	-48,9	-31,5
332-341	H7	-30,9	-52,4	-24,5	-58,7	-26,4	-51,6	-28,7	-61,1	-27,9	-59,7	-32,2	-56,5	-24,3	-51,1	-26,2	-55,0	-22,0	-44,1	-23,2	-32,7	-31,1	-44,4	-25,0	-53,1	-21,2	-45,1	-23,4	-49,0	-22,6	-50,7	-23,6	-39,9	-21,7	-43,3
334-341	H7	-32,2	-61,6	-24,4	-65,0	-25,1	-60,5	-27,0	-67,1	-27,8	-64,6	-25,0	-60,5	-23,8	-58,1	-24,8	-60,2	-22,3	-52,2	-23,8	-49,6	-30,4	-52,0	-25,3	-63,0	-21,4	-53,8	-21,7	-55,4	-24,9	-59,3	-19,2	-44,0	-21,2	-46,9
335-340	H7	-33,3	-59,2	-25,9	-62,2	-30,5	-58,3	-26,4	-61,6	-28,3	-59,5	-29,4	-60,5	-30,9	-55,5	-26,9	-53,6	-27,1	-47,3	-21,6	-48,6	-31,3	-48,6	-23,7	-53,4	-21,9	-51,0	-22,8	-48,9	-22,2	-49,9	-21,8	-48,4	-26,3	-44,6
335-341	H7	-30,2	-60,8	-25,2	-67,7	-26,7	-59,3	-25,7	-67,3	-25,7	-65,0	-28,7	-60,1	-27,0	-56,2	-25,2	-60,6	-24,5	-50,3	-20,9	-51,9	-27,8	-51,2	-22,9	-56,3	-21,3	-54,3	-20,9	-53,6	-22,3	-55,8	-22,7	-45,1	-24,2	-47,3
336-341	H7	-18,6	-66,4	-11,2	-68,2	-14,5	-62,6	-12,6	-69,1	-12,4	-69,4	-15,6	-66,6	-14,6	-60,8	-12,0	-66,8	-15,3	-60,2	-18,4	-63,9	-18,2	-59,8	-12,3	-65,3	-9,6	-62,2	-11,9	-65,8	-10,4	-63,7	-13,5	-54,5	-14,4	-52,6
341-348	H7	-26,9	-47,9	-18,1	-46,5	-25,7	-46,3	-22,2	-51,3	-21,1	-49,2	-24,0	-48,8	-23,4	-42,0	-19,6	-46,3	-20,2	-38,9	-24,8	-46,4	-26,9	-42,7	-21,0	-45,0	-16,7	-41,4	-21,4	-43,2	-17,6	-41,7	-20,8	-40,4	-23,0	-39,1
342-348	H7	-22,9	-41,5	-20,6	-47,4	-21,6	-40,7	-20,5	-51,7	-20,3	-49,5	-23,3	-43,5	-20,8	-39,6	-20,0	-46,5	-20,1	-37,3	-21,3	-40,6	-23,2	-36,6	-18,6	-45,2	-16,7	-40,5	-19,0	-43,9	-17,4	-42,3	-18,7	-32,9	-20,4	-35,8
349-353	loop H7-H8	-3,7	-16,2	-8,4	-25,0	-9,4	-24,4	-11,4	-26,5	-8,1	-19,7	5,0	-9,5	-3,0	-22,6	-6,6	-23,0	-5,7	-21,4	-0,5	-12,1	-4,4	-14,6	-8,9	-25,4	-7,1	-19,6	-10,8	-22,7	-7,9	-22,8	3,1	-10,0	-8,6	-31,0
349-356	loop H7-H8, H8	-8,5	-21,1	-6,7	-20,1	-10,3	-21,5	-3,0	-19,0	-5,4	-19,8	-11,3	-30,1	-10,8	-22,8	-4,7	-18,8	-9,7	-20,4	-7,7	-19,9	-7,9	-18,8	-5,7	-16,7	-4,2	-14,7	-3,0	-13,2	-3,9	-16,0	-9,9	-29,1	-11,0	-21,9
350-356	loop H7-H8, H8	-10,7	-20,1	-11,9	-17,2	-8,3	-11,4	-14,5	-17,9	-13,6	-16,4	-11,3	-21,6	-9,6	-12,2	-13,1	-16,0	-11,6	-9,8	-11,2	-20,1	-11,5	-16,5	-18,1	-17,5	-12,2	-16,0	-14,7	-17,8	-12,6	-17,0	-8,2	-19,8	-10,0	-10,0
352-356	H8	3,1	-3,1	-0,4	-6,0	1,9	-4,7	1,3	-5,8	0,8	-6,8	-0,1	-6,0	1,0	-4,2	0,9	-5,6	1,0	-4,1	3,4	-2,2	2,6	1,1	0,2	-4,9	0,5	-5,9	0,4	-5,8	1,1	-4,6	0,4	-3,0	0,7	-2,3
357-361	H8	4,2	-1,9	-1,2	-2,8	-0,2	-3,0	-2,9	-4,4	-2,7	-3,0	-0,7	-4,3	-0,8	-3,1	-1,3	-3,2	-0,8	-2,5	3,3	-2,3	2,8	-2,3	-1,6	-3,5	-0,8	-3,2	-2,7	-4,5	-2,9	-4,2	-0,5	-3,3	-0,4	-3,4
360-365	H8	-0,1	-7,7	-1,5	-5,6	-1,3	-5,7	-2,7	-6,1	-2,3	-6,2	-1,3	-4,8	-0,7	-4,9	-2,0	-5,8	-0,6	-4,3	-1,0	-8,0	-1,1	-6,3	-2,6	-6,2	-1,3	-5,2	-2,7	-6,0	-2,4	-4,6	-1,1	-3,8	-0,8	-4,4
362-376	H8, loop H8-H9	-19,4	-9,0	-14,9	-14,6	-18,5	-14,7	-16,6	-13,8	-16,7	-14,9	-22,0	-17,8	-16,1	-13,5	-14,8	-10,7	-12,7	-11,1	-13,3	-5,6	-18,6	-4,4	-16,0	-10,7	-13,9	-12,3	-17,4	-11,9	-16,4	-11,1	-18,1	-15,6	-16,2	-11,3
366-376	loop H8-H9, H9	-25,7	-10,7	-26,1	-15,6	-24,7	-13,1	-28,3	-16,8	-27,1	-15,3	-31,6	-18,6	-23,1	-10,2	-24,9	-10,3	-19,2	-9,9	-18,4	-5,4	-25,3	-5,5	-25,8	-11,4	-24,7	-13,9	-27,1	-13,4	-25,9	-13,5	-25,4	-16,0	-22,1	-11,7
366-377	loop H8-H9, H9	-23,9	-7,5	-19,5	-12,1	-34,8	-15,8	-22,0	-13,3	-21,4	-12,6	-37,8	-16,0	-29,7	-13,7	-18,0	-7,0	-26,2	-12,0	-16,9	-4,1	-24,0	-4,3	-20,8	-8,3	-18,6	-9,7	-20,7	-8,4	-19,3	-10,0	-32,5	-13,9	-28,0	-14,7
366-378	loop H8-H9, H9	-21,2	-10,2	-17,3	-13,3	-21,2	-15,5	-19,6	-14,1	-17,2	-13,1	-18,9	-12,7	-19,1	-12,8	-15,8	-8,3	-15,0	-11,5	-14,7	-6,8	-20,9	-5,5	-17,0	-8,8	-16,2	-11,2	-18,1	-11,6	-16,2	-10,7	-16,8	-9,3	-18,6	-12,8
377-386	H9	-6,1	-19,3	-4,3	-11,5	-6,9	-14,1	-6,0	-15,0	-5,6	-12,7	-9,3	-24,0	-5,6	-12,4	-4,3	-10,8	-3,7	-8,7	-5,0	-13,0	-4,2	-11,4	-5,1	-12,2	-4,6	-11,0	-6,1	-14,7	-5,0	-11,0	-7,5	-18,7	-3,8	-7,6

377-387	H9	-4,5	-16,3	-4,5	-14,5	-4,7	-15,7	-4,5	-16,0	-4,5	-15,2	-6,5	-16,7	-4,1	-14,3	-3,9	-12,8	-3,9	-11,3	-5,7	-14,5	-4,9	-12,7	-4,5	-13,2	-3,9	-13,2	-4,7	-14,9	-3,8	-12,3	-5,6	-13,6	-3,7	-9,9
377-389	H9	-3,2	-15,4	-2,7	-12,9	-4,9	-15,3	-3,1	-13,4	-3,0	-13,0	-5,8	-15,9	-4,4	-14,6	-3,0	-12,2	-4,3	-13,5	-4,2	-14,1	-3,8	-13,4	-3,6	-12,4	-3,0	-12,1	-2,4	-12,8	-2,5	-12,6	-5,4	-13,6	-4,4	-11,5
379-389	H9	-4,5	-10,6	-4,4	-12,5	-3,6	-10,3	-4,8	-12,1	-3,8	-12,6	-5,3	-16,8	-3,2	-8,7	-4,2	-11,3	-3,7	-7,0	-5,6	-11,3	-5,5	-7,0	-4,6	-11,8	-3,9	-10,3	-4,1	-12,5	-3,1	-9,5	-4,8	-13,9	-1,2	-5,7
382-389	H9	-5,1	-14,7	-4,4	-13,0	-3,7	-12,7	-8,2	-14,8	-7,3	-14,9	-4,9	-11,1	-2,8	-10,8	-7,1	-12,8	-2,8	-9,4	-5,9	-13,9	-5,3	-9,8	-8,4	-15,3	-4,2	-11,4	-8,8	-15,3	-7,7	-12,6	-3,3	-8,6	-2,9	-6,2
385-389	H9	-1,9	-11,5	-8,1	-13,9	-8,2	-16,1	-6,8	-15,0	-4,1	-13,8	-10,2	-11,1	-9,4	-13,4	-4,1	-11,7	-6,5	-7,9	-2,5	-10,7	-3,0	-8,6	-10,3	-15,8	-6,5	-16,8	-6,2	-15,4	-5,4	-14,8	-7,4	-3,8	-9,4	-11,2
390-404	H9-H10	-8,3	-13,7	-7,1	-15,5	-5,8	-9,7	-7,5	-14,9	-7,7	-16,1	-9,7	-14,8	-5,9	-9,6	-7,2	-14,6	-5,6	-11,3	-8,1	-14,1	-9,6	-10,5	-6,4	-14,3	-7,7	-16,6	-6,3	-15,1	-5,6	-13,7	-8,7	-12,8	-5,7	-8,2
392-404	H9-H10	-7,6	-11,3	-7,6	-14,2	-6,3	-8,1	-8,0	-13,7	-8,2	-15,3	-12,5	-16,0	-6,5	-8,1	-7,5	-13,1	-5,9	-10,6	-7,3	-11,6	-10,0	-7,8	-6,9	-13,1	-8,3	-15,5	-7,1	-13,4	-6,2	-12,5	-13,6	-14,2	-6,0	-7,4
392-407	H9-H10	-6,5	-17,5	-5,7	-18,2	-7,7	-16,3	-7,8	-17,2	-8,0	-16,6	-11,9	-20,9	-7,5	-15,7	-4,6	-13,6	-6,3	-14,6	-7,1	-14,3	-9,2	-13,7	-7,5	-16,0	-6,8	-16,7	-6,6	-14,6	-5,7	-16,5	-10,2	-18,2	-6,0	-15,0
408-413	H10/11	1,5	-3,0	-0,1	-8,7	-0,5	-9,6	1,8	-7,5	0,5	-8,0	-0,6	-5,9	-0,8	-6,2	0,6	-5,1	-0,9	-6,5	1,2	-3,1	2,3	-1,5	-0,3	-6,3	0,9	-4,7	1,9	-2,3	1,6	-4,0	0,3	-4,3	-1,8	-8,5
410-414	H10/11	2,6	-5,4	0,7	-11,7	-1,4	-11,6	3,7	-8,0	0,0	-12,1	-1,5	-11,6	-2,4	-9,6	0,1	-7,1	-3,9	-9,7	2,2	-1,3	0,5	-5,5	1,3	-7,2	1,3	-7,6	-1,8	-2,4	0,4	-4,9	-0,4	-8,2	-2,3	-10,1
412-420	H10/11	-19,4	-6,3	-25,0	-20,0	-18,2	-9,9	-22,3	-12,3	-22,4	-13,1	-25,2	-16,8	-23,0	-12,4	-16,8	-6,7	-15,7	-10,2	2,4	-4,2	-16,2	-3,1	-19,7	-8,9	-20,1	-11,2	-23,4	-12,0	-20,8	-10,9	-16,8	-12,0	-13,9	-9,2
412-426	H10/11	-41,0	-21,0	-48,9	-36,7	-40,2	-21,9	-47,8	-36,3	-48,0	-31,8	-47,5	-31,4	-44,1	-27,5	-40,9	-21,5	-25,9	-12,9	-2,9	-7,9	-37,3	-13,1	-41,0	-18,0	-40,5	-18,5	-42,4	-17,7	-40,6	-18,7	-31,6	-17,9	-32,6	-16,0
412-427	H10/11	-43,9	-21,7	-47,5	-36,5	-42,2	-22,5	-47,7	-36,7	-47,3	-32,6	-45,7	-31,0	-47,1	-29,2	-40,7	-21,5	-25,6	-13,7	-2,3	-7,1	-39,8	-12,2	-40,3	-16,7	-39,1	-16,9	-40,3	-15,5	-39,8	-17,9	-29,5	-14,5	-34,2	-16,2
415-426	H10/11	-60,2	-29,7	-58,3	-42,5	-54,7	-28,3	-58,9	-45,8	-58,7	-40,8	-58,1	-35,2	-59,2	-36,8	-51,6	-30,7	-35,1	-16,2	-9,3	-12,0	-54,9	-18,9	-51,2	-24,3	-49,8	-23,8	-51,9	-20,5	-50,8	-24,4	-38,5	-17,6	-46,2	-20,5
415-427	H10/11	-61,0	-29,5	-63,5	-42,2	-57,4	-26,6	-65,8	-47,1	-64,6	-40,1	-59,6	-33,8	-61,8	-36,5	-56,6	-27,9	-33,4	-13,1	-7,0	-9,8	-55,5	-16,8	-55,0	-20,2	-53,6	-19,2	-55,0	-17,0	-54,4	-20,6	-37,9	-15,0	-47,1	-17,6
427-439	H10/11-H12	-14,7	-7,2	-18,4	-10,2	-16,2	-5,8	-21,1	-13,1	-17,6	-10,2	-22,9	-13,0	-17,1	-7,0	-15,4	-6,9	-9,6	-3,1	-3,0	-2,8	-15,2	-0,6	-14,8	-3,7	-14,8	-5,4	-14,9	-3,0	-14,5	-3,2	-15,2	-7,6	-12,9	-2,1
427-441	H10/11-H12	-25,1	-9,6	-30,1	-17,0	-26,1	-6,3	-35,8	-22,9	-32,1	-16,0	-32,9	-17,2	-28,5	-12,1	-27,9	-10,5	-14,0	-4,9	-3,6	-4,0	-26,2	-3,4	-24,4	-3,8	-23,3	-6,5	-25,5	-1,3	-23,9	-3,8	-23,5	-12,2	-16,6	-3,2
427-442	H10/11-H12	-28,0	-13,8	-28,0	-17,3	-24,9	-12,4	-43,1	-19,5	-35,6	-20,8	-31,2	-18,8	-26,8	-15,9	-26,0	-12,8	-13,1	-8,7	-5,3	-5,2	-29,1	-6,6	-21,3	-6,4	-22,5	-9,2	-23,6	-4,0	-26,0	-6,2	-19,8	-12,3	-18,1	-6,8
427-443	H10/11-H12	-33,5	-15,9	-34,6	-21,9	-33,7	-12,4	-43,2	-29,0	-40,0	-21,7	-40,4	-20,5	-34,9	-19,4	-34,7	-14,1	-17,3	-4,6	-1,6	-5,1	-33,2	-7,6	-30,9	-4,6	-26,4	-3,7	-30,9	-1,6	-28,5	-3,7	-21,1	-7,7	-22,2	-1,3
428-439	H10/11-H12	-13,6	-6,5	-17,7	-10,5	-14,0	-3,3	-20,6	-13,2	-17,5	-10,5	-18,9	-10,6	-14,9	-4,8	-15,3	-6,2	-8,7	-1,5	-1,3	-1,1	-14,1	0,2	-14,5	-2,9	-14,1	-4,3	-15,0	-1,9	-14,1	-2,5	-12,1	-4,7	-10,3	0,0
428-440	H10/11-H12	-22,2	-10,7	-25,9	-14,6	-18,8	-5,4	-29,8	-19,2	-25,4	-13,3	-23,6	-10,4	-19,7	-10,6	-22,6	-6,7	-9,6	-4,9	-1,3	-2,8	-21,5	-0,9	-20,5	-2,6	-19,5	-4,9	-19,4	-2,1	-20,3	-1,7	-13,3	-4,0	-11,6	-2,9
428-441	H10/11-H12	-26,9	-9,4	-31,1	-18,3	-27,0	-6,7	-38,3	-27,1	-35,2	-19,5	-33,0	-15,6	-30,0	-13,5	-30,6	-12,5	-15,7	-4,4	-2,1	-2,7	-27,5	-3,0	-26,1	-3,3	-24,3	-6,9	-28,4	-4,2	-25,8	-5,3	-18,7	-7,3	-19,7	-2,9
440-447	H12	-36,9	-17,7	-47,0	-29,4	-41,7	-19,8	-49,2	-34,2	-45,5	-28,6	-42,4	-23,6	-40,9	-23,1	-40,9	-20,6	-27,4	-7,9	-5,3	-3,1	-38,9	-10,2	-40,5	-13,5	-38,9	-12,9	-40,1	-10,5	-39,7	-12,6	-25,7	-8,8	-32,3	-6,6
441-446	H12	-38,5	-25,7	-37,0	-19,7	-38,7	-9,2	-36,4	-26,6	-39,1	-23,6	-34,7	-18,9	-35,7	-13,5	-35,0	-16,9	-15,6	-0,5	-8,4	-8,5	-38,5	-16,4	-35,3	-11,4	-29,5	-7,2	-31,6	-9,1	-34,1	-6,9	-23,7	-8,6	-24,2	0,1
**LXRβ**																																			
218-226	H1	-3,4	-12,4	-6,0	-9,1	-11,7	-10,4	-17,2	-9,2	-18,5	-13,9	-3,4	-17,1	-10,2	-10,1	-16,5	-16,5	-10,4	-8,9	-3,9	-13,7	-3,7	-12,6	-2,5	-13,0	-14,7	-13,6	-13,9	-9,3	-15,2	-13,1	-5,4	-13,6	-9,0	-10,0
226-230	H1	-0,1	-8,4	-5,1	-18,5	-2,3	-11,3	-1,3	-14,2	-2,5	-21,4	2,5	-6,3	-1,5	-8,7	-2,7	-24,0	-1,4	-11,5	0,1	-11,6	-1,0	-5,8	-1,4	-9,2	-2,1	-17,1	-3,1	-8,0	-4,3	-19,5	3,2	-11,6	-1,0	-11,3
226-231	H1	-1,8	-8,5	-1,8	-13,1	-2,4	-8,5	-0,9	-10,9	-1,3	-17,4	-1,3	-10,4	-1,3	-8,6	-2,5	-16,1	-2,3	-9,1	-1,6	-10,1	-1,3	-8,2	-1,0	-8,0	-1,0	-11,6	-0,5	-6,5	-1,1	-14,8	-1,1	-12,4	-1,2	-7,6
226-233	H1	-1,8	-11,6	-1,8	-14,2	-1,9	-10,2	-1,1	-12,0	-1,8	-17,0	-1,8	-13,3	-0,7	-11,1	-1,5	-16,2	-1,6	-11,2	-2,1	-12,6	-1,7	-10,7	-1,7	-11,1	-1,2	-11,9	-1,1	-7,6	-1,8	-14,3	-2,0	-15,0	-1,2	-9,9
227-233	H1	-1,8	-10,2	-1,0	-13,0	-1,1	-10,9	1,8	-7,1	0,9	-11,9	-1,6	-11,2	-0,7	-11,0	0,2	-10,5	-0,8	-10,8	-2,0	-11,4	-1,6	-10,0	-1,6	-10,2	-0,2	-9,1	-0,4	-5,3	-0,3	-7,9	-1,3	-11,5	-0,7	-10,7
228-233	H1	-2,4	-14,8	-0,5	-13,8	-1,5	-10,5	0,4	-11,5	-0,7	-13,7	-2,1	-15,6	-0,6	-11,5	-0,3	-14,0	-0,8	-11,0	-3,1	-16,7	-2,3	-13,9	-3,1	-14,2	-0,8	-10,9	0,1	-10,7	-0,7	-11,9	-2,6	-15,9	-0,9	-11,5
234-238	H1	-4,6	-23,8	-4,0	-25,4	-1,1	-12,2	-5,0	-16,1	-7,1	-23,5	-4,0	-27,0	-3,6	-11,2	-5,8	-23,5	-5,1	-6,2	-5,9	-26,7	-9,2	-23,2	-3,6	-23,6	-2,1	-10,0	-2,6	-15,6	-3,5	-15,7	-6,5	-29,4	-9,7	-28,9
274-279	H3	-25,5	-87,7	-18,4	-80,4	-30,1	-79,2	-18,6	-80,2	-19,6	-81,1	-25,3	-87,2	-30,7	-79,8	-19,5	-80,4	-30,3	-80,2	-25,7	-89,1	-25,0	-87,3	-25,3	-87,2	-17,7	-79,6	-17,6	-77,5	-18,4	-77,1	-24,9	-85,7	-30,0	-76,2
274-281	H3	-31,8	-85,0	-20,6	-76,6	-29,4	-72,6	-21,2	-76,7	-21,3	-77,8	-31,2	-84,8	-29,1	-72,0	-21,9	-77,7	-29,3	-72,5	-32,1	-86,2	-31,2	-84,6	-31,8	-84,7	-19,9	-75,9	-19,8	-75,1	-21,0	-75,8	-31,1	-83,6	-28,9	-69,4
275-281	H3	-30,9	-80,3	-20,1	-70,7	-30,0	-74,0	-20,0	-70,6	-20,5	-71,4	-30,5	-80,7	-29,6	-73,5	-20,3	-70,5	-29,9	-73,7	-30,8	-81,0	-30,6	-79,8	-30,5	-80,2	-19,1	-69,7	-19,1	-66,6	-19,4	-67,1	-30,4	-79,1	-28,9	-70,0
281-285	H3	-1,0	-0,1	-2,5	-6,7	-1,1	-2,8	-1,1	-6,3	-2,3	-7,6	0,4	-1,6	-0,2	-2,6	-1,2	-8,7	0,2	-1,8	-0,6	-4,7	3,9	-4,3	1,5	-1,7	-0,2	-4,6	-2,3	-6,7	-3,2	-5,7	2,3	-0,8	-0,4	-4,4
285-292	H3, loop H3-H4	0,5	-2,8	-0,2	-2,7	-0,3	-2,9	0,0	-2,1	-0,6	-2,7	0,0	-2,8	0,1	-3,0	-0,2	-2,8	0,2	-2,6	0,8	-2,0	0,0	-2,9	0,3	-2,5	-0,2	-2,5	-0,2	-2,1	-0,1	-2,3	0,1	-3,3	0,3	-2,7
286-292	H3, loop H3-H4	1,9	-0,9	1,2	-1,6	1,2	-1,3	1,5	-0,6	1,0	-0,3	1,1	-1,2	2,2	-0,7	1,3	-0,8	1,6	-0,7	0,7	-1,8	1,3	-1,3	1,3	-1,0	1,6	-0,4	1,2	-0,9	1,6	-0,7	1,4	-1,6	1,8	-1,4
286-295	H3, loop H3-H4	-6,7	-7,3	-5,6	-5,6	-3,8	-3,8	-5,8	-5,2	-6,9	-5,6	-5,5	-6,1	-3,0	-3,6	-6,4	-5,4	-4,2	-3,5	-6,7	-7,4	-6,4	-7,3	-6,1	-6,3	-6,4	-6,0	-4,9	-5,2	-6,1	-5,3	-6,2	-7,3	-3,2	-3,8
290-295	H3, loop H3-H4	0,3	-9,4	-0,2	5,0	-3,4	2,8	-4,9	1,0	-10,8	-0,6	2,2	-8,7	-2,9	3,1	-9,9	-5,0	-3,5	2,1	0,2	-9,6	-2,7	-10,3	-1,6	-7,9	-1,3	3,2	-0,5	2,4	-7,5	4,1	-0,5	-10,2	-4,2	0,6
293-300	H4-H5	-4,4	-5,0	-4,2	-3,7	-3,5	-4,6	-7,0	-5,8	-8,0	-7,9	-4,1	-3,8	-2,1	-2,0	-6,6	-7,5	-2,2	-1,8	-4,5	-6,1	-5,2	-4,7	-4,4	-3,8	-8,0	-7,8	-6,8	-6,2	-7,5	-7,5	-3,9	-5,8	-3,4	-3,2
293-303	H4-H5	-8,6	-9,1	-5,3	-6,4	-5,1	-5,3	-5,4	-6,2	-6,0	-6,7	-7,9	-8,6	-4,7	-5,1	-5,6	-6,5	-4,7	-5,4	-8,6	-9,5	-8,7	-9,3	-8,4	-8,9	-5,7	-6,7	-5,6	-6,1	-5,8	-6,0	-8,0	-9,6	-5,0	-5,5
296-303	H5	-2,5	-2,7	-0,6	-1,0	-2,0	-1,4	-0,4	0,8	-1,3	-0,1	-2,1	-3,2	-0,8	-1,0	-0,8	-0,3	-1,8	-1,3	-2,7	-3,5	-3,1	-3,5	-2,7	-3,4	-0,7	0,4	-0,8	0,5	-0,2	-0,1	-2,6	-3,6	-1,7	-2,4
296-304	H5	-1,6	-1,3	-1,3	-1,7	-1,4	-0,7	-0,9	-0,6	-1,5	-1,5	-1,2	-1,2	-0,8	-0,5	-1,9	-1,2	-1,1	-0,4	-1,7	-1,6	-1,8	-1,6	-1,6	-1,2	-0,5	-1,2	-1,2	-1,2	-1,5	-1,3	-1,0	-2,0	-1,2	-0,9
304-308	H5	-30,9	-67,2	-24,5	-70,5	-33,8	-83,6	-23,4	-75,5	-24,3	-76,2	-31,5	-78,8	-31,4	-78,3	-23,1	-78,4	-32,6	-81,4	-25,6	-24,8	-32,0	-83,6	-31,4	-82,5	-23,6	-70,8	-20,2	-63,4	-23,0	-61,8	-31,1	-79,8	-31,1	-77,8
305-310	H5	-26,9	-38,1	-25,5	-53,3	-33,1	-52,2	-24,6	-53,6	-27,4	-53,8	-33,1	-52,3	-32,7	-50,0	-25,0	-59,7	-30,8	-40,6	-16,8	-9,1	-34,0	-53,7	-30,1	-54,2	-24,0	-40,9	-25,2	-45,9	-23,1	-32,2	-28,4	-50,4	-33,0	-53,8
313-326	H5, βsheets	-27,5	-28,8	-27,5	-34,7	-24,4	-32,9	-23,1	-23,7	-28,9	-35,5	-31,1	-41,7	-27,1	-37,1	-28,4	-34,6	-25,9	-36,8	-32,1	-44,2	-31,7	-41,8	-30,5	-39,5	-14,3	-5,3	-19,9	-20,5	-21,3	-14,5	-30,0	-41,6	-23,4	-30,2
314-326	H5, βsheets	-28,4	-30,5	-28,8	-36,8	-27,4	-37,8	-23,6	-27,3	-29,6	-37,4	-33,9	-43,9	-27,0	-37,7	-29,1	-36,9	-27,6	-38,6	-34,9	-46,5	-34,1	-44,1	-32,7	-42,0	-17,4	-11,8	-20,9	-24,5	-21,9	-18,2	-31,9	-42,4	-24,4	-32,0
330-340	βsheets, H6	-41,8	-45,6	-45,7	-48,7	-43,5	-46,6	-29,9	-34,1	-45,2	-45,7	-48,1	-45,5	-44,9	-46,3	-42,9	-47,0	-45,3	-47,8	-52,5	-54,3	-48,4	-49,6	-46,0	-48,2	-43,7	-38,4	-30,5	-26,2	-39,1	-29,7	-45,6	-44,8	-38,6	-33,7
333-340	βsheets, H6	-48,0	-58,7	-40,7	-50,9	-43,2	-51,1	-37,9	-40,9	-39,4	-49,0	-46,1	-54,9	-41,6	-50,6	-39,3	-46,3	-43,1	-52,8	-50,7	-62,3	-47,1	-53,1	-46,1	-53,5	-41,1	-45,0	-34,0	-29,4	-37,2	-35,1	-44,7	-49,3	-42,9	-41,2
333-345	βsheets, H6	-64,5	-62,3	-44,0	-43,8	-55,1	-48,3	-51,2	-42,1	-53,3	-51,7	-64,8	-59,7	-52,9	-47,6	-51,8	-47,9	-44,6	-35,1	-50,1	-44,3	-65,0	-58,1	-58,0	-51,4	-44,3	-41,7	-44,1	-29,6	-40,6	-30,6	-61,2	-52,3	-52,2	-37,7
333-346	βsheets, H6	-72,9	-71,2	-50,6	-47,6	-68,9	-55,2	-56,4	-42,6	-61,0	-60,9	-73,5	-66,5	-64,4	-58,0	-60,9	-55,5	-56,2	-37,0	-46,8	-43,9	-72,1	-60,9	-64,9	-50,8	-51,0	-44,4	-49,6	-31,9	-49,3	-33,3	-68,0	-54,4	-64,9	-45,1
341-346	H6-H7	-63,1	-48,7	-47,8	-31,1	-65,2	-36,8	-57,1	-24,4	-66,0	-50,0	-65,8	-48,6	-61,2	-40,0	-64,3	-46,6	-43,7	-4,0	-15,2	-2,9	-65,2	-45,7	-55,9	-28,9	-47,6	-24,7	-53,1	-14,8	-45,2	-19,9	-61,6	-39,6	-56,9	-21,1
347-355	H7	-42,0	-83,2	-40,9	-70,9	-41,3	-69,1	-42,7	-72,1	-43,3	-75,0	-41,8	-80,3	-41,2	-69,6	-42,7	-73,7	-41,5	-65,6	-24,2	-46,8	-41,9	-78,1	-41,4	-73,5	-40,3	-63,2	-39,5	-58,2	-42,7	-63,7	-40,5	-72,5	-40,4	-65,7
348-355	H7	-35,7	-77,8	-24,7	-67,8	-32,2	-65,3	-26,3	-68,3	-27,0	-70,8	-35,6	-73,0	-31,9	-62,7	-26,3	-71,7	-32,2	-60,0	-24,4	-51,9	-34,8	-72,2	-34,7	-68,8	-23,3	-62,1	-24,9	-58,1	-25,5	-62,5	-34,2	-66,6	-30,2	-62,4
349-354	H7	-31,9	-78,4	-26,2	-63,5	-31,3	-56,5	-25,6	-65,2	-26,3	-64,1	-32,3	-73,9	-30,6	-56,7	-25,6	-61,1	-29,1	-51,0	-16,3	-61,3	-32,1	-70,9	-32,1	-66,3	-24,4	-57,2	-22,1	-49,5	-25,3	-58,8	-30,9	-64,9	-29,5	-54,6
349-355	H7	-38,6	-76,7	-30,2	-67,6	-33,6	-65,5	-30,1	-66,3	-30,6	-65,6	-37,8	-71,9	-32,0	-62,9	-30,6	-63,5	-32,6	-60,7	-24,2	-57,1	-37,5	-68,6	-37,7	-66,3	-29,4	-59,9	-27,0	-54,5	-29,7	-58,9	-36,8	-62,0	-31,5	-61,8
350-354	H7	-20,3	-73,9	-13,9	-65,7	-14,7	-64,3	-9,6	-62,2	-9,8	-61,0	-19,2	-71,1	-13,5	-63,6	-8,9	-57,9	-14,8	-65,5	-20,6	-64,4	-20,2	-69,6	-18,2	-66,8	-10,9	-61,0	-9,6	-60,0	-10,4	-56,7	-16,0	-64,9	-13,8	-64,3
350-355	H7	-23,7	-77,0	-15,9	-70,3	-21,1	-68,8	-16,3	-69,6	-16,9	-68,7	-23,0	-74,2	-20,9	-66,3	-16,9	-67,2	-21,0	-70,7	-23,2	-67,5	-22,9	-71,0	-22,8	-70,9	-14,7	-66,3	-15,5	-67,2	-16,2	-63,5	-22,4	-65,3	-20,3	-66,8
356-369	H7-H8	-3,1	-11,5	-6,2	-11,7	-3,0	-13,4	-6,0	-7,8	-6,6	-9,4	-4,8	-16,9	1,1	-10,7	-9,3	-11,1	-1,6	-11,7	-6,9	-17,2	-9,1	-18,5	-7,6	-17,8	-4,1	-6,4	-9,0	-9,5	-0,7	-2,1	-8,4	-19,9	-2,3	-12,5
357-369	H7-H8	-1,4	-9,1	-3,4	-11,8	-3,2	-11,5	-3,0	-8,5	-4,3	-11,3	-2,8	-13,0	0,3	-9,7	-7,0	-11,1	-1,7	-11,0	-5,1	-14,9	-7,8	-15,0	-6,1	-14,2	-0,6	-6,1	-5,0	-9,3	4,2	-2,3	-5,9	-15,7	-2,5	-11,1
360-369	H7-H8	-5,8	-7,6	-2,3	2,4	-1,7	-4,1	-2,2	4,6	-3,3	4,1	-6,3	-6,6	2,1	-3,0	-4,2	3,9	0,4	-3,1	-6,5	-8,4	-8,8	-7,0	-6,9	-6,0	-1,4	3,3	-4,8	4,9	-0,3	4,1	-6,8	-8,6	-0,8	-3,8
374-378	H8	1,1	3,8	-1,4	-1,4	-1,3	-0,8	-2,0	-2,4	-4,0	-3,5	1,9	2,8	-1,0	-0,7	-4,3	-3,0	-0,7	-0,6	0,7	-0,7	2,1	2,2	-0,3	3,7	-0,8	-1,3	-1,7	-2,1	-1,9	-1,5	1,9	3,8	-0,9	-0,1
374-379	H8	-0,5	-0,6	-0,3	-0,7	-0,7	-1,3	-1,1	-1,6	-1,1	-2,0	0,0	-0,9	-1,3	-1,9	-1,4	-2,1	-0,5	-1,8	-0,2	-1,1	-0,3	-0,5	-0,2	-0,8	0,0	-1,2	-1,0	-1,5	-1,1	-1,2	-0,5	-0,7	-1,0	-0,9
374-394	H8-H9	-9,9	-13,0	-5,2	-7,4	-4,1	-5,7	-6,8	-8,3	-6,5	-8,6	-8,7	-12,2	-2,7	-5,1	-6,8	-9,1	-2,7	-3,8	-8,6	-10,1	-9,4	-12,1	-9,4	-12,8	-6,6	-8,3	-6,8	-8,4	-6,4	-7,4	-8,7	-13,6	-4,0	-7,3
376-394	H8-H9	-11,4	-15,2	-6,9	-10,2	-5,6	-7,4	-7,9	-11,4	-7,9	-11,6	-9,7	-13,8	-4,4	-7,4	-7,6	-11,4	-4,1	-6,6	-9,9	-12,1	-11,1	-14,3	-10,5	-13,9	-8,1	-11,3	-8,0	-11,2	-7,5	-9,6	-9,8	-15,3	-5,5	-10,0
378-394	H8-H9	-14,6	-14,7	-8,0	-10,0	-7,9	-9,9	-9,6	-13,4	-9,7	-9,7	-12,7	-12,6	-6,1	-8,6	-8,5	-10,5	-6,6	-6,5	-13,1	-10,5	-14,5	-13,3	-13,6	-12,9	-9,1	-10,0	-9,8	-13,0	-8,9	-10,4	-12,6	-14,7	-8,2	-11,0
379-394	H8-H9	-13,6	-20,3	-8,4	-12,4	-7,3	-7,7	-10,2	-13,8	-10,5	-14,0	-12,0	-18,8	-6,1	-7,4	-9,1	-12,9	-5,4	-6,4	-11,8	-16,6	-13,5	-19,2	-12,4	-18,0	-9,9	-13,6	-9,8	-14,0	-9,8	-10,9	-11,4	-19,9	-7,5	-10,4
380-394	H8-H9	-8,2	-13,2	-6,8	-10,4	-5,5	-5,8	-7,8	-12,2	-7,4	-10,8	-6,7	-11,3	-4,8	-6,9	-7,4	-11,5	-4,1	-4,5	-7,0	-7,9	-7,6	-11,5	-7,0	-11,3	-8,2	-11,9	-7,9	-12,2	-7,5	-9,2	-6,1	-12,8	-5,7	-10,2

380-401	H8-H9	-10,1	-15,5	-6,0	-12,1	-6,2	-9,1	-6,9	-13,5	-7,0	-13,5	-8,5	-14,4	-5,8	-9,0	-6,5	-13,6	-5,3	-8,6	-10,1	-12,8	-9,9	-15,1	-9,2	-14,6	-7,3	-13,2	-7,1	-13,6	-6,7	-11,7	-8,9	-16,0	-6,2	-11,6
380-403	H8-H9	-10,2	-15,5	-5,8	-10,6	-4,7	-7,6	-6,3	-11,4	-6,2	-11,4	-8,8	-14,0	-4,3	-7,5	-6,2	-11,4	-4,1	-6,9	-9,7	-12,8	-9,7	-14,8	-9,3	-14,4	-6,6	-11,0	-6,4	-11,5	-6,1	-9,8	-8,8	-15,0	-4,9	-9,6
389-394	H9	-12,8	-14,9	-3,7	-6,5	-6,0	-4,3	-7,3	-6,6	-7,6	-7,6	-8,7	-10,4	-4,7	-3,9	-7,8	-11,7	-1,5	-2,6	-10,9	-16,7	-12,7	-14,3	-13,4	-11,9	-5,3	-2,1	-5,4	-7,3	-5,8	-5,9	-10,4	-13,0	-4,3	-5,0
393-403	H9	1,5	-0,5	2,6	1,9	-0,6	-0,2	1,6	-0,3	-0,3	-0,7	1,0	-0,5	-1,2	-0,5	-0,1	0,0	0,6	0,1	0,4	-0,9	1,3	-1,5	2,1	0,2	2,3	1,9	1,8	1,7	0,5	0,3	-0,2	-1,3	0,3	-0,3
395-401	H9	-1,3	-2,9	-1,3	-3,1	-0,6	-1,2	0,2	-1,6	-1,2	-2,1	-1,3	-4,6	-1,0	-2,1	-1,7	-3,3	-0,1	-1,5	-1,8	-3,4	-0,1	-2,8	-0,2	-3,9	-0,7	-2,4	-0,6	-2,2	-0,5	-1,1	-1,4	-3,7	-1,0	-3,0
395-403	H9	-1,2	-3,4	-0,3	-3,0	-0,8	-3,3	-0,4	-2,5	-0,2	-3,1	-1,8	-3,6	0,5	-2,5	-0,5	-2,4	0,1	-3,0	-1,9	-3,3	-1,6	-3,9	-2,0	-4,1	0,0	-2,7	-0,5	-2,8	-0,5	-1,7	-1,7	-3,7	0,2	-2,8
404-418	H9-H10	-6,0	-7,3	-6,4	-3,6	-6,6	-4,5	-7,3	-2,5	-9,3	-3,3	-6,3	-6,3	-4,0	-3,3	-7,6	-4,0	-5,0	-3,8	-5,4	-7,3	-6,5	-7,1	-5,3	-5,9	-6,1	-3,1	-5,6	-2,1	-5,5	-2,9	-5,4	-8,0	-1,8	-4,3
405-418	H9-H10	-9,6	-11,0	-5,4	-6,7	-9,2	-6,7	-7,4	-5,9	-10,9	-8,1	-10,0	-9,2	-6,6	-5,4	-7,9	-8,7	-7,6	-5,5	-8,9	-10,6	-10,4	-10,9	-9,1	-9,3	-5,5	-6,6	-5,8	-5,4	-6,8	-9,3	-9,7	-11,3	-5,0	-5,9
406-418	H9-H10	-6,0	-5,8	-5,5	1,5	-7,4	-5,8	-6,8	3,0	-8,0	4,4	-6,3	-6,7	-3,7	-4,6	-8,6	2,3	-5,3	-3,6	-8,0	-5,6	-6,0	-3,8	-5,2	-5,2	-6,3	3,7	-5,2	4,0	-4,3	2,4	-7,7	-8,6	-2,5	-5,5
406-421	H9-H10	-9,5	-16,2	-9,9	-15,0	-8,8	-10,0	-10,5	-11,9	-12,3	-12,6	-10,3	-15,6	-6,4	-10,0	-11,4	-13,5	-7,2	-9,6	-8,8	-9,8	-9,8	-16,3	-9,2	-15,3	-9,9	-13,0	-9,3	-12,2	-8,9	-11,4	-9,0	-17,3	-4,2	-10,8
407-421	H9-H10	-9,8	-15,1	-11,5	-16,6	-8,7	-11,1	-12,0	-14,0	-12,0	-13,7	-9,5	-16,9	-6,1	-10,4	-11,8	-15,6	-6,2	-9,6	-8,5	-10,9	-9,1	-17,2	-9,2	-16,0	-10,7	-14,9	-10,2	-14,1	-9,1	-13,1	-8,7	-18,4	-3,0	-10,7
423-428	H10/11	-4,0	-4,6	-4,6	-10,4	-5,3	-5,8	-4,3	-6,2	-6,9	-11,3	-2,8	-5,7	-2,5	0,9	-4,8	-13,5	-3,0	-2,9	0,0	-1,4	-2,7	-6,0	-3,1	-7,7	0,1	-2,1	-2,5	-9,2	-2,0	-10,2	-2,3	-4,9	-3,1	-4,7
424-428	H10/11	-7,2	-10,0	-5,8	-15,7	-9,1	-10,4	-5,5	-11,6	-8,8	-17,0	-4,6	-12,1	-3,6	-5,8	-6,7	-19,8	-5,3	-7,5	-0,7	-1,5	-5,9	-11,3	-5,8	-12,2	-1,3	-7,7	-4,6	-12,3	-5,2	-13,2	-5,2	-11,2	-5,0	-8,2
426-434	H10/11	-36,3	-43,5	-33,6	-37,0	-30,8	-23,3	-31,1	-30,5	-35,8	-34,8	-36,0	-40,3	-33,2	-32,3	-30,6	-32,5	-29,2	-21,8	-14,1	-15,4	-37,4	-38,9	-36,2	-38,9	-31,2	-31,7	-32,2	-30,6	-30,1	-31,0	-33,5	-35,6	-31,9	-30,6
426-440	H10/11	-62,5	-59,0	-57,3	-53,7	-56,9	-37,6	-55,9	-49,3	-57,3	-52,6	-62,7	-55,5	-59,9	-50,8	-54,6	-48,1	-47,1	-21,7	-23,7	-22,6	-62,5	-53,4	-61,8	-49,8	-54,8	-46,6	-54,4	-36,5	-54,1	-42,6	-58,8	-49,0	-56,2	-38,3
426-441	H10/11	-64,7	-58,4	-59,4	-53,9	-58,6	-34,8	-58,1	-48,5	-59,1	-52,8	-64,0	-52,5	-62,2	-50,6	-56,8	-47,2	-47,3	-18,8	-19,5	-18,0	-64,5	-51,2	-63,7	-46,6	-56,6	-46,0	-55,0	-32,4	-56,1	-41,7	-60,3	-47,1	-57,9	-35,9
428-440	H10/11	-74,9	-64,5	-65,2	-56,4	-65,9	-41,8	-65,6	-52,7	-66,8	-57,2	-74,5	-60,6	-68,8	-55,2	-65,2	-51,8	-54,0	-24,3	-29,8	-30,7	-74,6	-57,5	-73,6	-53,3	-63,3	-49,9	-62,6	-37,2	-63,9	-44,3	-72,1	-52,3	-64,9	-41,3
429-440	H10/11	-76,0	-67,5	-69,1	-60,0	-70,8	-46,1	-68,1	-56,1	-69,3	-60,1	-75,7	-61,7	-75,6	-61,9	-67,4	-54,7	-58,3	-22,1	-26,9	-25,4	-75,5	-59,7	-74,0	-54,1	-68,1	-52,7	-65,2	-37,1	-66,3	-46,0	-72,4	-53,8	-71,0	-43,0
429-441	H10/11	-76,7	-66,8	-70,2	-58,9	-72,3	-40,9	-69,7	-54,3	-70,9	-59,4	-76,1	-58,9	-77,1	-59,6	-69,5	-52,7	-58,4	-18,0	-22,7	-22,4	-76,0	-56,6	-74,5	-50,6	-69,0	-50,3	-65,1	-32,1	-68,1	-44,9	-72,9	-52,1	-72,5	-39,5
441-453	H10/11-H12	-28,0	-19,7	-17,0	-11,6	-16,8	-9,6	-17,6	-11,9	-19,5	-12,0	-27,1	-16,8	-20,5	-11,5	-17,9	-10,9	-15,5	-3,9	-12,6	-11,4	-28,0	-19,4	-23,6	-16,4	-17,1	-9,1	-15,3	-3,9	-17,0	-6,7	-25,3	-17,7	-18,0	-6,8
441-456	H10/11-H12	-50,0	-30,8	-46,2	-38,4	-41,9	-24,8	-47,5	-40,4	-46,2	-34,6	-52,2	-27,5	-42,9	-26,3	-45,7	-33,9	-35,0	-9,0	-16,8	-12,4	-50,3	-31,7	-47,5	-26,5	-46,9	-34,8	-42,7	-30,6	-45,2	-30,9	-47,7	-26,6	-40,9	-14,0
441-457	H10/11-H12	-55,0	-32,7	-42,5	-23,8	-37,5	-26,7	-44,9	-28,2	-46,1	-30,2	-53,6	-29,3	-37,8	-25,1	-45,1	-29,4	-28,1	-3,6	-12,2	-9,8	-56,1	-36,5	-52,1	-29,4	-42,2	-21,4	-38,9	-5,9	-43,6	-15,8	-51,7	-30,6	-31,9	-11,5
442-453	H10/11-H12	-24,9	-16,0	-16,8	-11,3	-16,9	-8,0	-18,1	-11,6	-19,6	-11,7	-23,7	-14,0	-18,4	-9,7	-18,2	-10,6	-15,5	-0,9	-8,7	-7,6	-24,9	-15,8	-21,5	-13,4	-17,2	-7,8	-15,7	-2,3	-17,1	-5,6	-22,8	-14,5	-16,9	-4,8
442-456	H10/11-H12	-52,4	-31,9	-44,6	-25,8	-37,6	-23,9	-48,2	-30,7	-50,6	-32,5	-52,0	-29,4	-35,7	-21,4	-49,6	-29,9	-25,9	-1,7	-12,5	-9,9	-53,6	-34,3	-50,9	-28,1	-46,8	-24,8	-44,5	-8,6	-44,6	-16,4	-50,7	-28,6	-29,1	-7,8
454-461	H12	-49,7	-32,9	-47,3	-27,8	-49,0	-31,3	-46,8	-31,9	-46,5	-32,0	-50,7	-32,1	-47,9	-30,0	-45,5	-31,3	-41,3	-9,5	-15,5	-10,7	-52,3	-36,0	-50,6	-31,8	-48,2	-30,9	-44,4	-16,4	-46,4	-24,0	-47,9	-31,8	-45,9	-18,7

Supplementary Table 5: Peptides with highest contribution to the discrimination between lipogenic and non-lipogenic compounds.
Related to Figure 3

Peptide	LXR isoform & Labelling Time	Amino Acid Sequence	Secondary Structure	OPLS-DA		Lipogenic (n = 5) vs non-lipogenic (n = 7): unpaired t-test		
				p	p(corr)	P-value	P-value summary	t, df*
**TG OPLS-DA: LXRα/SRC1 and LXRβ/SRC1 HDX-MS**								
239-257	LXRα/SRC1/30 sec	RVTPWPMAPDPHSREA RQQRFAHF	loop H1-H3, H3	0,056996	0,740641	0,0061	**	t=3,265, df=13
319-331	LXRα/SRC1/30 sec	FSYNREDFAKAGL	βsheets-H6	0,076847	0,822716	0,0017	**	t=3,945, df=13
326-331	LXRα/SRC1/30 sec	FAKAGL	H6	0,142916	0,711162	0,0089	**	t=3,072, df=13
327-331	LXRα/SRC1/30 sec	AKAGL	H6	0,131653	0,742891	0,0037	**	t=3,523, df=13
427-442	LXRα/SRC1/30 sec	ALRLQDKKLPPLLSEI	H10/11-H12	0,101271	0,702581	0,0153	*	t=2,790, df=13
427-443	LXRα/SRC1/30 sec	ALRLQDKKLPPLLSEIW	H10/11-H12	0,117756	0,698298	0,0094	**	t=3,046, df=13
260-265	LXRα/SRC1/600 sec	LAIVSV	H3	0,135231	0,801881	0,003	**	t=3,635, df=13
261-267	LXRα/SRC1/600 sec	AIVSVQE	H3	0,117213	0,800223	0,0027	**	t=3,687, df=13
321-326	LXRα/SRC1/600 sec	YNREDF	βsheets-H6	0,069397	0,787831	0,0016	**	t=3,983, df=13
319-331	LXRα/SRC1/600 sec	FSYNREDFAKAGL	βsheets-H6	0,154177	0,911449	<0,0001	****	t=6,064, df=13
320-331	LXRα/SRC1/600 sec	SYNREDFAKAGL	βsheets-H6	0,176587	0,889276	0,0001	***	t=5,497, df=13
321-331	LXRα/SRC1/600 sec	YNREDFAKAGL	βsheets-H6	0,1991	0,9126	<0,0001	****	t=6,465, df=13
327-331	LXRα/SRC1/600 sec	AKAGL	H6	0,224377	0,824784	0,0004	***	t=4,771, df=13
334-340	LXRα/SRC1/600 sec	EFINPIF	H7	0,183499	0,757711	0,0018	**	t=3,898, df=13
334-341	LXRα/SRC1/600 sec	EFINPIFE	H7	0,094322	0,77736	0,0027	**	t=3,692, df=13
335-340	LXRα/SRC1/600 sec	FINPIF	H7	0,066668	0,76997	0,0034	**	t=3,568, df=13
335-341	LXRα/SRC1/600 sec	FINPIFE	H7	0,079184	0,900451	<0,0001	****	t=6,845, df=13
336-341	LXRα/SRC1/600 sec	INPIFE	H7	0,077918	0,845374	0,0002	***	t=5,033, df=13
412-426	LXRα/SRC1/600 sec	VSLRTLSSVHSEQVF	H10/11	0,119165	0,831866	0,0005	***	t=4,640, df=13
412-427	LXRα/SRC1/600 sec	VSLRTLSSVHSEQVFA	H10/11	0,131013	0,866482	0,0001	***	t=5,328, df=13
414-426	LXRα/SRC1/600 sec	LRTLSSVHSEQVF	H10/11	0,144189	0,872449	0,0001	***	t=5,511, df=13
415-426	LXRα/SRC1/600 sec	RTLSSVHSEQVF	H10/11	0,167289	0,895212	<0,0001	****	t=6,025, df=13
415-427	LXRα/SRC1/600 sec	RTLSSVHSEQVFA	H10/11	0,052973	0,837297	0,0002	***	t=5,080, df=13
427-439	LXRα/SRC1/600 sec	ALRLQDKKLPPLL	H10/11-H12	0,092414	0,866215	0,0004	***	t=4,788, df=13
428-439	LXRα/SRC1/600 sec	LRLQDKKLPPLL	H10/11-H12	0,087049	0,930961	<0,0001	****	t=7,148, df=13
427-441	LXRα/SRC1/600 sec	ALRLQDKKLPPLLSE	H10/11-H12	0,13526	0,94579	<0,0001	****	t=7,141, df=13
428-440	LXRα/SRC1/600 sec	LRLQDKKLPPLLS	H10/11-H12	0,056373	0,824116	0,0007	***	t=4,416, df=13
427-442	LXRα/SRC1/600 sec	ALRLQDKKLPPLLSEI	H10/11-H12	0,076216	0,840913	0,0003	***	t=4,839, df=13
428-441	LXRα/SRC1/600 sec	LRLQDKKLPPLLSE	H10/11-H12	0,102263	0,841338	0,0006	***	t=4,474, df=13
427-443	LXRα/SRC1/600 sec	ALRLQDKKLPPLLSEIW	H10/11-H12	0,13172	0,903497	<0,0001	****	t=5,843, df=13
440-447	LXRα/SRC1/600 sec	SEIWDVHE	H12	0,098766	0,7811	0,0024	**	t=3,750, df=13

Significance selection criteria included correlation coefficients (|p(corr)| > 0.7) and the coefficients of contribution to the model separation between TG classes (p > 0.05).

* t: t-values, df: degrees of freedom

Supplementary Table 6: Peptides with highest contribution to the discrimination between compounds inducing high and low levels of the intestine *Abca1* . *Related to Figure 4*

Significance selection criteria included correlation coefficients (|p(corr)| > 0.7) and the coefficients of contribution to the model separation between ABCA1 classes (p > 0.05).

Peptide	LXR isoform & Labelling Time	Amino Acid Sequence	Secondary Structure	OPLS-DA		High ABCA1 (n = 12) vs low ABCA1 (n = 4): unpaired t-test		
				p	p(corr)	P-value	P-value summary	t, df*
**ABCA1 OPLS-DA: LXRα and LXRβ HDX-MS**								
**260-265**	LXRα/30 sec	LAIVSV	H3	0,150564	0,894756	<0,0001	****	t=6,545, df=14
**261-267**	LXRα/30 sec	AIVSVQE	H3	0,137346	0,904123	<0,0001	****	t=5,645, df=14
**264-269**	LXRα/30 sec	SVQEIV	H3	0,161037	0,927907	<0,0001	****	t=6,163, df=14
**270-275**	LXRα/30 sec	DFAKQL	H3	0,096683	0,744891	0,0003	***	t=4,766, df=14
**312-316**	LXRα/30 sec	SITFL	βsheets	0,138416	0,80345	0,0002	***	t=5,049, df=14
**319-324**	LXRα/30 sec	FSYNRE	βsheets-H6	0,088788	0,781694	0,0006	***	t=4,444, df=14
**319-326**	LXRα/30 sec	FSYNREDF	βsheets-H6	0,085006	0,724336	0,0028	**	t=3,611, df=14
**320-326**	LXRα/30 sec	SYNREDF	βsheets-H6	0,09595	0,735341	0,0014	**	t=3,970, df=14
**319-331**	LXRα/30 sec	FSYNREDFAKAGL	βsheets-H6	0,108478	0,700074	0,0027	**	t=3,629, df=14
**320-331**	LXRα/30 sec	SYNREDFAKAGL	βsheets-H6	0,118371	0,778358	0,0003	***	t=4,852, df=14
**260-265**	LXRα/600 sec	LAIVSV	H3	0,275759	0,87557	0,0008	***	t=4,267, df=14
**261-267**	LXRα/600 sec	AIVSVQE	H3	0,215162	0,89756	0,0004	***	t=4,624, df=14
**264-269**	LXRα/600 sec	SVQEIV	H3	0,205503	0,861406	0,001	**	t=4,124, df=14
**270-275**	LXRα/600 sec	DFAKQL	H3	0,080182	0,718343	0,0027	**	t=3,636, df=14
**290-296**	LXRα/600 sec	LKTSAIE	H5	0,099353	0,737769	0,0004	***	t=4,563, df=14
**299-311**	LXRα/600 sec	LLETSRRYNPGSE	H5-βsheets	0,074506	0,728003	0,0012	**	t=4,039, df=14
**299-312**	LXRα/600 sec	LLETSRRYNPGSES	H5-βsheets	0,074421	0,699539	0,004	**	t=3,432, df=14
**300-311**	LXRα/600 sec	LETSRRYNPGSE	H5-βsheets	0,074396	0,750937	0,0006	***	t=4,389, df=14
**300-312**	LXRα/600 sec	LETSRRYNPGSES	H5-βsheets	0,092217	0,806184	0,0004	***	t=4,671, df=14
**299-314**	LXRα/600 sec	LLETSRRYNPGSESIT	H5-βsheets	0,095125	0,798007	0,0014	**	t=3,964, df=14
**299-315**	LXRα/600 sec	LLETSRRYNPGSESITF	H5-βsheets	0,098119	0,791755	0,0022	**	t=3,738, df=14
**300-315**	LXRα/600 sec	LETSRRYNPGSESITF	H5-βsheets	0,102035	0,796625	0,0018	**	t=3,851, df=14
**316-326**	LXRα/600 sec	LKDFSYNREDF	βsheets-H6	0,07239	0,784793	0,0058	**	t=3,250, df=14
**319-324**	LXRα/600 sec	FSYNRE	βsheets-H6	0,078487	0,731671	0,0232	*	t=2,549, df=14
**319-326**	LXRα/600 sec	FSYNREDF	βsheets-H6	0,107692	0,860933	0,0011	**	t=4,087, df=14
**320-326**	LXRα/600 sec	SYNREDF	βsheets-H6	0,112167	0,846516	0,0024	**	t=3,685, df=14
**321-326**	LXRα/600 sec	YNREDF	βsheets-H6	0,137569	0,908762	0,0001	***	t=5,358, df=14
**319-331**	LXRα/600 sec	FSYNREDFAKAGL	βsheets-H6	0,106789	0,885911	0,0003	***	t=4,861, df=14
**320-331**	LXRα/600 sec	SYNREDFAKAGL	βsheets-H6	0,120963	0,892896	<0,0001	****	t=5,382, df=14
**321-331**	LXRα/600 sec	YNREDFAKAGL	βsheets-H6	0,113321	0,785537	0,0018	**	t=3,909, df=13
**333-341**	LXRα/600 sec	VEFINPIFE	H7	0,160406	0,795071	0,0002	***	t=4,931, df=14
**334-341**	LXRα/600 sec	EFINPIFE	H7	0,136552	0,752029	0,0007	***	t=4,303, df=14
**335-341**	LXRα/600 sec	FINPIFE	H7	0,122503	0,719301	0,0017	**	t=3,874, df=14
**341-348**	LXRα/600 sec	EFSRAMNE	H7	0,127799	0,758272	0,0008	***	t=4,277, df=14
**342-348**	LXRα/600 sec	FSRAMNE	H7	0,116114	0,777342	0,0004	***	t=4,557, df=14

* t: t-values, df: degrees of freedom

Supplementary Table 7: Average differences in deuteration levels of the LXR/NCOR1 complexes in comparison with the apo states.
Related to Figure 5

Sequence	Start	End	MaxUptake, Da	ΔHDX (apo - apo/NCOR1), % 30 sec	60 sec	180 sec	600 sec	1800 sec	P-value
			LXRα vs. LXRα/NCOR1						
GSILPQLSPEQLGM	200	213	7,65	2,37	4,37	2,28	-0,6	1,91	1,22E-01
PQLSPEQLGM	204	213	4,95	0,23	2,72	0,74	-2,03	0,82	5,51E-01
IEKLVA	214	219	3,31	-8,26	-8,45	-8,56	-5,82	0,25	9,15E-09
IEKLVAAQQQCNRRSFSDRL	214	233	11,39	-8,89	-2,08	-4,45	-6,58	-0,15	7,57E-03
AQQQCNRRSFSDRL	220	233	7,11	-6,87	0,12	-2,89	-6,98	-1,68	1,85E-01
QQQCNRRSFSDRL	221	233	6,33	-7,5	0,12	-1,03	-6,57	0,13	1,81E-01
RRSFSDRL	226	233	2,49	-2,11	1,82	0,82	-1,03	2,27	9,64E-01
RVTPWPM	234	240	2,5	-5,22	-0,95	-4,51	-7,91	-1,33	2,37E-01
RVTPWPMAPDPHSRE	234	248	5,5	-4,8	1,6	-1,52	-3,15	-0,9	4,64E-01
RVTPWPMAPDPHSREARQQRFA	234	257	8,96	-5,11	-0,85	-2,77	-5,64	-1,39	2,09E-01
PMAPDPHSREARQQRFAHF	239	257	6,4	-8,41	-0,33	-0,52	-4,49	2,2	6,79E-02
APDPHSREARQQRFAHF	241	257	6,6	-3,17	2,89	-0,28	-2,75	2,03	7,85E-01
LAIVSV	260	265	3,69	-19,97	-19,92	-21,49	-22,67	2,47	1,39E-10
AIVSVQE	261	267	4,24	-18,41	-16,89	-21,56	-17,76	-3,8	2,32E-11
SVQEIV	264	269	3,03	-15,98	-11,86	-15,02	-15,03	-5,9	2,27E-08
FAKQLPGF	271	278	3,66	-0,81	-0,83	-1,96	-4,59	-6,37	7,64E-01
FAKQLPGFLQL	271	281	5,91	0,49	2,63	-0,04	-2,51	-2,51	1,61E-01
AKQLPGF	272	278	3	-0,4	-0,11	-0,26	-3,88	-4,9	8,23E-01
AKQLPGFLQL	272	281	5,13	-1,61	-1,18	-2,85	-5,74	-5,14	2,39E-01
QLSREDQIALL	280	290	6,25	-0,98	-1,35	-2,8	-3,64	-6,27	3,41E-01
SREDQIAL	282	289	4,17	-0,01	-0,23	-2,03	-4,67	-2,46	6,25E-01
SREDQIALL	282	290	4,71	4,35	3,73	1,64	-1,45	1,74	9,12E-07
LKTSAIE	290	296	3,84	-12,42	-17,2	-19,8	-14,88	-5,35	3,73E-09
IEVML	295	299	2,33	-1,38	-1,68	-1,22	-4,22	-4,4	1,17E-01
LLETSRRYNPGSE	299	311	6,27	-3	-1,17	-2,22	-4,3	-2,36	1,02E-01
LLETSRRYNPGSES	299	312	7,69	-4,62	-2,17	-3,12	-4,19	-3,04	4,97E-03
LLETSRRYNPGSESIT	299	314	9,05	-5,26	-3,51	-5,85	-6,37	-3,46	7,00E-03
LLETSRRYNPGSESITF	299	315	9,65	-5,3	-3,53	-4,64	-6,78	-3,02	4,28E-03
LETSRRYNPGSE	300	311	5,67	-1,83	-0,7	-1,29	-2,95	-1,07	3,15E-01
LETSRRYNPGSES	300	312	6,29	-3,31	-1,36	-1,48	-3,18	0,03	8,59E-02
LETSRRYNPGSESITF	300	315	8,89	-7,05	-4,92	-5,51	-6,81	-2,83	3,70E-04
TSRRYNPGSE	302	311	4,74	0,05	2,88	0,54	-0,97	3,39	5,21E-01
SITFL	312	316	2,41	-7,2	-3,26	-4,32	-3,97	0,73	1,13E-02
LKDFSYNREDF	316	326	5,29	-8,89	-3,82	-7,48	-9,21	-3,27	1,05E-03
FSYNREDFAKAGL	319	331	7,31	-9,93	-4,67	-6,32	-7,61	-0,42	2,81E-04
SYNREDFAKAGL	320	331	5,57	-11,01	-6,51	-6,63	-7,53	-3,94	4,03E-04
YNREDF	321	326	2,69	-8,78	-6,55	-8,67	-8,76	-4,19	1,20E-03
YNREDFAKAGL	321	331	4,35	-17,91	-11,24	-6,1	-6,27	0,43	3,16E-06
FAKAGL	326	331	2,99	-3,65	0,42	0,75	-1,35	2,66	3,04E-01
AKAGL	327	331	2,29	-4,73	0,74	0,34	-0,09	2,94	3,01E-01
QVEFINPIFE	332	341	4,6	-1,87	-3,98	-4,37	-6	-4,27	9,40E-02
VEFINPIFE	333	341	4,26	-2,29	-7,92	-4,02	-8,06	-0,19	5,46E-02
EFINPIFE	334	341	3,6	-4,3	-7,86	-6,27	-9,97	-2,28	1,36E-03
FINPIF	335	340	2,28	-5,69	-2,68	-8,4	-9,78	-4,17	1,59E-03
FINPIFE	335	341	3,03	-3,69	-5,68	-9,11	-11,22	-4,06	3,94E-04
INPIFE	336	341	2,44	-3,35	-5,25	-8,66	-11,63	-3,45	1,37E-03
FSRAMNE	342	348	3,61	0,54	-0,86	-1,88	-4,7	1,35	7,76E-01
LQLND	349	353	2,39	0,13	-0,27	-0,51	-0,8	2,45	7,07E-01
LQLNDAE	349	355	3,84	-2,56	-0,85	-3,73	-3,2	-1,85	7,27E-02
NDAEF	352	356	2,11	0,48	0,51	0,1	-3,91	-0,44	6,15E-01
ALLIA	357	361	2,4	-1,78	-1,18	-2	-3,48	-3,48	2,09E-03
IAISIF	360	365	3,57	-1,18	-1,11	-1,6	-3,25	-2,96	1,86E-02
ISIFSA	362	367	3,23	0,51	2,06	2,07	-4,65	-4,77	3,53E-01
ISIFSADRPNVQDQL	362	376	8,91	-4,11	-4,5	-4,8	-6,81	-3,94	1,06E-02
SADRPNVQDQL	366	376	5,75	-2,5	0,29	0,18	-3,64	-0,2	5,98E-01
SADRPNVQDQLQ	366	377	6,55	2,13	3,32	0,29	-4,15	-0,7	3,31E-01
SADRPNVQDQLQV	366	378	6,94	1,12	0,74	-0,29	-4,27	-0,88	6,50E-01
QVERLQHTY	377	385	3,75	2,19	1,63	2,42	3,44	4,58	1,11E-01
QVERLQHTYV	377	386	4,76	1,95	1,69	0,18	2,08	-0,29	5,23E-02
QVERLQHTYVE	377	387	5,69	-0,74	-1,78	-1,79	-3,21	-2,29	8,68E-02
QVERLQHTYVEAL	377	389	7,23	-1,18	-2,2	-2,96	-4,38	-4,4	1,07E-02
ERLQHTYVEAL	379	389	4,88	1,08	-1,46	-3,62	-2,88	-6,75	9,03E-01
HAYVSIHHPHDRL	390	402	3,44	-4,04	-2,1	-2,12	-5,52	-7,22	3,15E-01
HAYVSIHHPHDRLMF	390	404	4,55	-5,22	-2,94	-5,02	-9,46	-4,83	1,13E-03
YVSIHHPHDRL	392	402	2	-7,46	-8,1	-10,34	-13,72	-3,78	7,91E-03
YVSIHHPHDRLMF	392	404	3,52	-3,28	-2,19	-2,98	-7,01	-3,76	4,46E-02
YVSIHHPHDRLMFPRM	392	407	5,42	-4,5	-3,84	-3,67	-7,38	-1,22	3,24E-04
LMKLVS	408	413	3,26	0,39	0,73	-1,11	-3,13	1,3	9,75E-01
KLVSL	410	414	2,38	-2,31	-1,01	-3,8	-4,4	2,58	2,91E-01
VSLRTLSSV	412	420	5,5	-2,05	-2,28	-2,01	-7,54	-5,23	4,27E-01
VSLRTLSSVHSEQVF	412	426	7,83	-6,52	-1,34	-3,63	-8,56	-3,43	6,31E-02
VSLRTLSSVHSEQVFA	412	427	8,48	-6,64	-2,03	-4,01	-8,68	-3,39	3,55E-02
LRTLSSVHSEQVF	414	426	6,07	-5,44	-2,12	-3,95	-6,76	-0,52	9,60E-02
LRTLSSVHSEQVFA	414	427	6,73	-7,21	-1	-3,74	-9,85	-3,38	7,39E-02
RTLSSVHSEQVF	415	426	5,54	-6,01	-2,93	-3,7	-8,75	-3,25	8,02E-02
RTLSSVHSEQVFA	415	427	6,1	-6,73	-1	-2,96	-7,72	-1,73	1,19E-01
ALRLQDKKLPPLL	427	439	5,94	-3,27	0,84	-0,63	-4,14	0,74	5,82E-01
ALRLQDKKLPPLLS	427	440	7,11	-4,46	0,27	-1,37	-6,48	0,26	3,93E-01
ALRLQDKKLPPLLSE	427	441	7,35	-5,07	0,61	0,69	-4,52	1,02	4,71E-01
ALRLQDKKLPPLLSEI	427	442	8,13	-1,6	1,99	1,27	-2,94	1,11	9,91E-01
ALRLQDKKLPPLLSEIW	427	443	8,37	0,32	3,55	1,53	0,03	2,5	6,36E-01
LRLQDKKLPPLL	428	439	5,27	-2,26	1,14	-0,54	-2,25	1,86	6,87E-01

LRLQDKKLPPLLS	428	440	6,2	-5	-0,21	-1,9	-5,43	0,33	2,90E-01
LRLQDKKLPPLLSE	428	441	6,37	-1,13	1,41	1,07	-3,67	2,48	9,49E-01
LRLQDKKLPPLLSEI	428	442	6,78	-3,02	0,41	0,38	-4,43	0,88	6,64E-01
LRLQDKKLPPLLSEIW	428	443	7,78	1,23	3,25	2,38	-0,96	0,81	5,15E-01
SEIWDVHE	440	447	4,19	-5,62	-0,4	-2,55	-6,79	-1,5	2,28E-01
WDVHE	443	447	1,8	0,15	2,79	3,91	-0,34	1,04	5,55E-01
**LXRβ vs. LXRβ/NCOR1**									
VQLTA	218	222	2,77	1,27	1,7	1,22	0,43	0,71	4,12E-01
VQLTAAQE	218	225	5,16	2,24	0,96	-0,82	-0,25	-1,97	3,03E-01
VQLTAAQEL	218	226	5,72	3,28	2,67	1,1	2,83	-2,34	2,19E-02
TAAQEL	221	226	3,25	5,17	4,17	0,42	6,09	2,8	3,70E-02
LMIQQ	226	230	2,48	0,22	-4,57	-2,55	-1,69	-10,75	6,97E-01
LMIQQLVA	226	233	4,22	0,03	-0,48	-2,12	-2,62	-12,99	7,65E-01
MIQQLVA	227	233	4,25	-0,18	-0,66	-2,5	-3,33	-12,52	8,89E-01
IQQLVA	228	233	3,49	-1,84	-1,84	-1,68	-3,45	-13,07	5,34E-01
VAAQL	232	236	2,86	1,37	-0,32	-1,83	-4,1	-3,88	7,79E-01
AQLQC	234	238	2,5	4,31	3,51	5,16	3,64	1,43	2,95E-02
LAIISV	274	279	3,58	-12,05	-15,92	-22,68	-23,66	-15,73	4,43E-09
AIISVQE	275	281	4,25	-12,52	-13,9	-19,37	-21,55	-14,17	2,44E-13
FAKQVPGF	285	292	3,57	2,85	2,35	1,57	1,93	-1,43	4,18E-03
FAKQVPGFLQL	285	295	6,01	-0,86	-2,69	-1,42	-2,13	-5,89	4,72E-01
AKQVPGF	286	292	2,81	0,06	0,5	0	-1,41	-6,38	2,57E-01
AKQVPGFLQL	286	295	5,35	-0,51	-0,89	-0,96	-2,16	-5,37	2,15E-02
LQLGREDQ	293	300	3,8	-0,94	-0,76	-0,7	-1,29	-3,11	6,60E-01
GREDQIAL	296	303	3,86	-0,42	-0,35	-1,25	-0,76	-2,71	6,27E-01
GREDQIALL	296	304	4,48	0,33	-0,01	-0,65	-0,23	-1,72	5,53E-01
LLKASTIE	303	310	4,59	-12,41	-17,52	-21,04	-16,82	-14,99	5,34E-10
LKASTIE	304	310	3,83	-16,85	-21	-21,36	-12,81	-3,72	1,26E-10
KASTIE	305	310	2,96	-14,79	-15,34	-17,22	-13,91	-4,25	9,89E-10
LLETARRYNHETE	313	325	5,43	7,3	3,94	0,62	2,14	-2,41	3,34E-05
LLETARRYNHETEC	313	326	6,16	7,72	4,43	1,51	2,13	0,28	4,24E-07
LETARRYNHETEC	314	326	5,28	6,07	4,19	0,81	0,31	-0,77	1,73E-04
LKDFTYSKDDF	330	340	4,82	-0,27	-1,19	-4,05	-4,91	-6,77	9,74E-01
FTYSKDDF	333	340	3,63	-1,19	-1,25	-2,81	-5,23	-6,83	8,15E-01
FTYSKDDFHRAGL	333	345	5,41	1,74	1,53	-2,06	-5,33	-5,68	1,22E-01
FTYSKDDFHRAGLQ	333	346	6,1	1,07	2,25	-0,71	-3,18	-4,4	1,72E-01
YSKDDF	335	340	2,46	-1,33	-1,04	-2,81	-5,75	-9,58	6,87E-01
HRAGLQV	341	347	3,22	0,81	5,13	2,25	-1,67	-3,64	6,73E-02
HRAGLQVE	341	348	3,54	2,75	5,39	3,3	1,25	-0,56	1,33E-01
VEFINPIFE	347	355	4,41	2,98	0,69	-0,05	0,93	-0,36	1,72E-01
EFINPIFE	348	355	3,67	-0,48	0,74	0,99	0,88	1,15	8,67E-01
FINPIF	349	354	2,38	-0,19	-1,64	-3,18	-3,76	-1,25	4,43E-01
FINPIFE	349	355	3,07	0,2	-1,5	-1,87	-0,23	-2,11	5,78E-01
INPIFE	350	355	2,46	-1,19	-2,04	-0,58	1,78	0,06	4,22E-01
FSRAMRRLGLDDAE	356	369	6,84	-0,72	-1,06	-1,99	-1,99	-4,39	7,96E-01
SRAMRRLGLDDAE	357	369	6,61	-2,91	-0,77	-1,7	-2,45	-6,34	3,96E-01
MRRLGLDDAE	360	369	4,45	-0,23	0,12	-0,91	0,28	-0,46	9,07E-01
IAINI	374	378	2,77	-0,02	-1,89	-0,94	-0,79	-1,07	6,47E-02
IAINIF	374	379	3,5	-0,52	-0,87	-0,91	-1,43	-1,2	2,65E-03
IAINIFSADRPNVQEPGRVEA	374	394	12,22	0,2	-2,02	-3	-3,45	-4,59	5,83E-01
INIFSADRPNVQEPGRVEA	376	394	10,97	-1,3	-1,94	-3,09	-3,92	-4,62	8,75E-02
IFSADRPNVQEPGRVEA	378	394	8,94	-3,32	-2,33	-2,11	-3,18	-5,67	1,21E-01
FSADRPNVQEPGRVEA	379	394	8,19	-1,02	-2,01	-2,92	-2,48	-2,93	2,58E-01
SADRPNVQEPGRV	380	392	6,59	-0,01	-2,14	-2,7	-2,37	-4,14	5,59E-01
SADRPNVQEPGRVEA	380	394	7,84	-0,44	-2,4	-3,52	-3,46	-4,22	2,34E-01
SADRPNVQEPGRVEALQQPYVE	380	401	12,75	-2,21	-3,32	-3,87	-5,29	-6,93	3,66E-04
SADRPNVQEPGRVEALQQPYVEA	380	403	13,97	-1,29	-2,72	-3,38	-3,52	-5,83	3,40E-03
PGRVEA	389	394	2,76	-0,04	-1,98	-4,04	-0,86	-1,64	5,01E-01
LQQPYVE	395	401	3,48	0,09	-0,17	-1,04	-1,48	-7,21	5,90E-01
LSYTRIKRPQDQLRF	404	418	8,54	-0,61	-1,94	-2,38	-3,68	-6,09	5,23E-01
SYTRIKRPQDQLRF	405	418	7,77	0,69	-2,19	-2,68	-2,52	-5,14	9,45E-01
YTRIKRPQDQLRF	406	418	6,77	-3,19	-3,38	-6,15	-3,64	-4,53	1,16E-02
YTRIKRPQDQLRFPRM	406	421	8,57	-0,98	-2,14	-2,54	-2,37	-6,72	4,44E-01
TRIKRPQDQLRFPRM	407	421	7,88	-1,71	-3,69	-4,95	2,17	-2,87	5,61E-02
KLVSL	424	428	2,43	-1,14	-3,92	-4,76	-3,35	-5,02	3,85E-01
VSLRTLSSV	426	434	5,58	-3,78	-4,33	-4,76	-4,66	-4,91	7,39E-03
VSLRTLSSVHSEQVF	426	440	7,96	-4,62	-4,16	-5,54	-7,15	-9,55	6,07E-03
VSLRTLSSVHSEQVFA	426	441	8,66	-2,88	-3,43	-4,81	-6,02	-8,46	9,08E-02
LRTLSSVHSEQVF	428	440	6,12	-5,55	-8,26	-7,3	-8,86	-10,3	8,86E-04
RTLSSV	429	434	2,99	0,78	0,23	0,24	0,62	-2,21	5,07E-01
RTLSSVHSEQVF	429	440	5,56	-4,72	-4,52	-6,37	-6,92	-8,74	9,75E-03
RTLSSVHSEQVFA	429	441	6,14	-4,09	-4,48	-5,85	-6,5	-8,57	2,31E-02
ALRLQDKKLPPLL	441	453	6,08	-2,86	-0,52	-2,05	-3,28	-3,89	6,84E-01
ALRLQDKKLPPLLS	441	454	6,89	-1,23	-1,51	-1,82	-1,62	-2,49	7,70E-01
ALRLQDKKLPPLLSEI	441	456	7,48	1,35	3,05	0,02	2,14	-3,07	3,24E-01
ALRLQDKKLPPLLSEIW	441	457	8,52	2,45	0,95	-0,12	1,44	1,02	4,27E-01
LRLQDKKLPPLL	442	453	5,37	0,35	0,35	-1,7	-0,04	-0,97	8,37E-01
SEIWDVHE	454	461	4,24	-0,48	-0,75	-1,78	-3,96	-2,1	8,33E-01
WDVHE	457	461	1,85	3,07	-0,82	-0,43	0,44	-1,03	4,00E-01

Number of replicates: 3
Number of exposure times: 6
% of deuterium: 95 %
Statistical significance value: 1 %
Statistically significant peptides are highlighted in grey

Supplementary Table 8: Contributions of LXRα and LXRβ peptides to separation between lipogenic and non-lipogenic classes of ligands (TG p(corr)) and ***Related to Figure 6***

Peptide	LXR isoform & Labelling Time	Amino Acid Sequence	Secondary Structure	TG p(corr)	ABCA1 p(corr)
**260-265**	LXRα/30 sec	LAIVSV	H3	0,182522	0,894756
**261-267**	LXRα/30 sec	AIVSVQE	H3	0,159467	0,904123
**264-269**	LXRα/30 sec	SVQEIV	H3	0,188553	0,927907
**316-326**	LXRα/30 sec	LKDFSYNREDF	βsheets-H6	0,284438	0,666177
**260-265**	LXRα/10 min	LAIVSV	H3	0,115661	0,87557
**261-267**	LXRα/10 min	AIVSVQE	H3	0,172573	0,89756
**264-269**	LXRα/10 min	SVQEIV	H3	0,109048	0,861406
**299-312**	LXRα/10 min	LLETSRRYNPGSES	H5-βsheets	0,215267	0,695628
**300-312**	LXRα/10 min	LETSRRYNPGSES	H5-βsheets	0,288253	0,806184
**299-314**	LXRα/10 min	LLETSRRYNPGSESIT	H5-βsheets	0,214434	0,798007
**299-315**	LXRα/10 min	LLETSRRYNPGSESITF	H5-βsheets	0,189743	0,791755
**300-315**	LXRα/10 min	LETSRRYNPGSESITF	H5-βsheets	0,203299	0,796625
**316-326**	LXRα/10 min	LKDFSYNREDF	βsheets-H6	0,079581	0,784793
**319-324**	LXRα/10 min	FSYNRE	βsheets-H6	0,208335	0,731671
**319-326**	LXRα/10 min	FSYNREDF	βsheets-H6	0,199409	0,860933
**320-326**	LXRα/10 min	SYNREDF	βsheets-H6	0,178388	0,846516
**321-326**	LXRα/10 min	YNREDF	βsheets-H6	0,284359	0,908762
**249-257**	LXRα/30 sec	ARQQRFAHF	H3	0,418542	0,676627
**299-314**	LXRα/30 sec	LLETSRRYNPGSESIT	H5-βsheets	0,417924	0,52989
**299-315**	LXRα/30 sec	LLETSRRYNPGSESITF	H5-βsheets	0,396382	0,632145
**300-315**	LXRα/30 sec	LETSRRYNPGSESITF	H5-βsheets	0,353796	0,605197
**312-316**	LXRα/30 sec	SITFL	βsheets	0,468028	0,80345
**319-324**	LXRα/30 sec	FSYNRE	βsheets-H6	0,450246	0,781694
**319-326**	LXRα/30 sec	FSYNREDF	βsheets-H6	0,482254	0,724336
**320-326**	LXRα/30 sec	SYNREDF	βsheets-H6	0,468115	0,735341
**321-326**	LXRα/30 sec	YNREDF	βsheets-H6	0,432617	0,63457
**299-311**	LXRα/10 min	LLETSRRYNPGSE	H5-βsheets	0,331435	0,728003
**300-311**	LXRα/10 min	LETSRRYNPGSE	H5-βsheets	0,302295	0,750937
**321-331**	LXRα/10 min	YNREDFAKAGL	βsheets-H6	0,435873	0,687789
**329-339**	LXRβ/10 min	LKDFTYSKDDF	βsheets, H6	0,417366	0,545559
**332-338**	LXRβ/10 min	FTYSKDD	βsheets, H6	0,336483	0,504645
**332-339**	LXRβ/10 min	FTYSKDDF	βsheets, H6	0,424319	0,624182
**334-339**	LXRβ/10 min	YSKDDF	βsheets, H6	0,392926	0,509046
**270-275**	LXRα/30 sec	DFAKQL	H3	0,693751	0,744891
**319-331**	LXRα/30 sec	FSYNREDFAKAGL	βsheets-H6	0,565468	0,698778
**320-331**	LXRα/30 sec	SYNREDFAKAGL	βsheets-H6	0,638015	0,778358
**321-331**	LXRα/30 sec	YNREDFAKAGL	βsheets-H6	0,572388	0,574976
**326-331**	LXRα/30 sec	FAKAGL	H6	0,678162	0,654782
**327-331**	LXRα/30 sec	AKAGL	H6	0,639354	0,649576
**270-275**	LXRα/10 min	DFAKQL	H3	0,624923	0,718343
**319-331**	LXRα/10 min	FSYNREDFAKAGL	βsheets-H6	0,532743	0,885911
**320-331**	LXRα/10 min	SYNREDFAKAGL	βsheets-H6	0,593985	0,892896
**326-331**	LXRα/10 min	FAKAGL	H6	0,694186	0,625754
**334-340**	LXRα/10 min	EFINPIF	H7	0,620582	0,677953
**334-341**	LXRα/10 min	EFINPIFE	H7	0,562785	0,752029
**335-340**	LXRα/10 min	FINPIF	H7	0,614977	0,663262
**335-341**	LXRα/10 min	FINPIFE	H7	0,567325	0,719301
**336-341**	LXRα/10 min	INPIFE	H7	0,503321	0,666359
**341-348**	LXRα/10 min	EFSRAMNE	H7	0,530387	0,758272
**342-348**	LXRα/10 min	FSRAMNE	H7	0,685637	0,777342
**313-325**	LXRβ/10 min	LETARRYNHETEC	H5, βsheets	0,623272	0,527395
**333-344**	LXRβ/10 min	TYSKDDFHRAGL	βsheets, H6	0,635216	0,541832
**290-296**	LXRα/10 min	LKTSAIE	H5	0,743081	0,737769
**327-331**	LXRα/10 min	AKAGL	H6	0,759729	0,692876
**333-341**	LXRα/10 min	VEFINPIFE	H7	0,727585	0,795071
**332-344**	LXRβ/10 min	FTYSKDDFHRAGL	βsheets, H6	0,733755	0,522827
**340-347**	LXRβ/10 min	HRAGLQVE	H6-H7	0,738019	0,506801
**412-426**	LXRα/30 sec	VSLRTLSSVHSEQVF	H10/11	0,715587	0,19401
**412-427**	LXRα/30 sec	VSLRTLSSVHSEQVFA	H10/11	0,721315	0,234654
**414-426**	LXRα/30 sec	LRTLSSVHSEQVF	H10/11	0,734507	0,246694
**414-427**	LXRα/30 sec	LRTLSSVHSEQVFA	H10/11	0,74986	0,288152
**415-426**	LXRα/30 sec	RTLSSVHSEQVF	H10/11	0,74078	0,281914
**415-427**	LXRα/30 sec	RTLSSVHSEQVFA	H10/11	0,748133	0,308221
**427-442**	LXRα/30 sec	ALRLQDKKLPPLLSEI	H10/11-H12	0,692639	0,329802
**428-441**	LXRα/30 sec	LRLQDKKLPPLLSE	H10/11-H12	0,773292	0,303054
**427-443**	LXRα/30 sec	ALRLQDKKLPPLLSEIW	H10/11-H12	0,775786	0,350804
**428-442**	LXRα/30 sec	LRLQDKKLPPLLSEI	H10/11-H12	0,741346	0,310625
**428-443**	LXRα/30 sec	LRLQDKKLPPLLSEIW	H10/11-H12	0,711789	0,228448

**440-447**	LXRα/30 sec	SEIWDVHE	H12	0,825616	0,269342
**441-446**	LXRα/30 sec	EIWDVH	H12	0,920834	0,405315
**214-219**	LXRα/10 min	IEKLVA	H1	0,825104	0,483172
**332-344**	LXRβ/30 sec	FTYSKDDFHRAGL	βsheets, H6	0,887247	0,289212
**332-345**	LXRβ/30 sec	FTYSKDDFHRAGLQ	βsheets, H6	0,870471	0,244108
**340-345**	LXRβ/30 sec	HRAGLQ	H6-H7	0,818952	0,144587
**340-346**	LXRβ/30 sec	HRAGLQV	H6-H7	0,837063	0,063208
**340-347**	LXRβ/30 sec	HRAGLQVE	H6-H7	0,791383	0,109854
**340-348**	LXRβ/30 sec	HRAGLQVEF	H6-H7	0,833864	-0,01266
**348-353**	LXRβ/30 sec	FINPIF	H7	0,705961	0,055966
**348-354**	LXRβ/30 sec	FINPIFE	H7	0,70169	0,103884
**427-439**	LXRβ/30 sec	LRTLSSVHSEQVF	H10/11	0,695051	-0,2291
**428-439**	LXRβ/30 sec	RTLSSVHSEQVF	H10/11	0,717002	-0,20853
**428-440**	LXRβ/30 sec	RTLSSVHSEQVFA	H10/11	0,724354	-0,20452
**440-452**	LXRβ/30 sec	ALRLQDKKLPPLL	H10/11-H12	0,713708	-0,19998
**440-453**	LXRβ/30 sec	ALRLQDKKLPPLLS	H10/11-H12	0,713626	-0,1705
**440-456**	LXRβ/30 sec	ALRLQDKKLPPLLSEIW	H10/11-H12	0,723603	-0,21667
**303-309**	LXRβ/10 min	LKASTIE	H5	0,698917	-0,19051
**304-309**	LXRβ/10 min	KASTIE	H5	0,7409	-0,15521
**332-345**	LXRβ/10 min	FTYSKDDFHRAGLQ	βsheets, H6	0,736355	0,453479
**340-345**	LXRβ/10 min	HRAGLQ	H6-H7	0,796951	0,351468
**340-346**	LXRβ/10 min	HRAGLQV	H6-H7	0,756441	0,303586
**340-348**	LXRβ/10 min	HRAGLQVEF	H6-H7	0,768548	0,356207
**347-354**	LXRβ/10 min	EFINPIFE	H7	0,718621	0,196037
**348-353**	LXRβ/10 min	FINPIF	H7	0,79585	0,164408
**348-354**	LXRβ/10 min	FINPIFE	H7	0,769606	0,190399
**425-433**	LXRβ/10 min	VSLRTLSSV	H10/11	0,712973	-0,20832
**425-439**	LXRβ/10 min	VSLRTLSSVHSEQVF	H10/11	0,792896	0,010273
**425-440**	LXRβ/10 min	VSLRTLSSVHSEQVFA	H10/11	0,7865	0,038314
**427-439**	LXRβ/10 min	LRTLSSVHSEQVF	H10/11	0,785936	0,061934
**428-439**	LXRβ/10 min	RTLSSVHSEQVF	H10/11	0,803832	0,122333
**428-440**	LXRβ/10 min	RTLSSVHSEQVFA	H10/11	0,785316	0,133228
**214-219**	LXRα/30 sec	IEKLVA	H1	0,679262	0,297033
**333-341**	LXRα/30 sec	VEFINPIFE	H7	0,610977	0,408623
**335-340**	LXRα/30 sec	FINPIF	H7	0,678346	0,255072
**412-420**	LXRα/30 sec	VSLRTLSSV	H10/11	0,568705	-0,09631
**415-420**	LXRα/30 sec	RTLSSV	H10/11	0,614924	0,049547
**412-424**	LXRα/30 sec	VSLRTLSSVHSEQ	H10/11	0,496817	0,138156
**333-344**	LXRβ/30 sec	TYSKDDFHRAGL	βsheets, H6	0,682076	0,224845
**347-354**	LXRβ/30 sec	EFINPIFE	H7	0,577512	0,111056
**425-433**	LXRβ/30 sec	VSLRTLSSV	H10/11	0,510977	-0,29788
**425-439**	LXRβ/30 sec	VSLRTLSSVHSEQVF	H10/11	0,661735	-0,25375
**425-440**	LXRβ/30 sec	VSLRTLSSVHSEQVFA	H10/11	0,665791	-0,27201
**440-455**	LXRβ/30 sec	ALRLQDKKLPPLLSEI	H10/11-H12	0,685638	-0,32356
**453-460**	LXRβ/30 sec	SEIWDVHE	H12	0,609005	-0,37618
**454-459**	LXRβ/30 sec	EIWDVH	H12	0,496634	-0,47956
**454-460**	LXRβ/30 sec	EIWDVHE	H12	0,603427	-0,32202
**273-278**	LXRβ/10 min	LAIISV	H3	0,4962	0,028651
**273-280**	LXRβ/10 min	LAIISVQE	H3	0,543743	0,225202
**274-280**	LXRβ/10 min	AIISVQE	H3	0,520806	0,229433
**303-307**	LXRβ/10 min	LKAST	H5	0,652175	-0,22507
**312-324**	LXRβ/10 min	LLETARRYNHETE	H5, βsheets	0,628957	0,424607
**312-325**	LXRβ/10 min	LLETARRYNHETEC	H5, βsheets	0,591779	0,431342
**346-354**	LXRβ/10 min	VEFINPIFE	H7	0,661985	0,303505
**349-354**	LXRβ/10 min	INPIFE	H7	0,519429	-0,02464

between high and low ABCA1 inducers (ABCA1 p(corr))

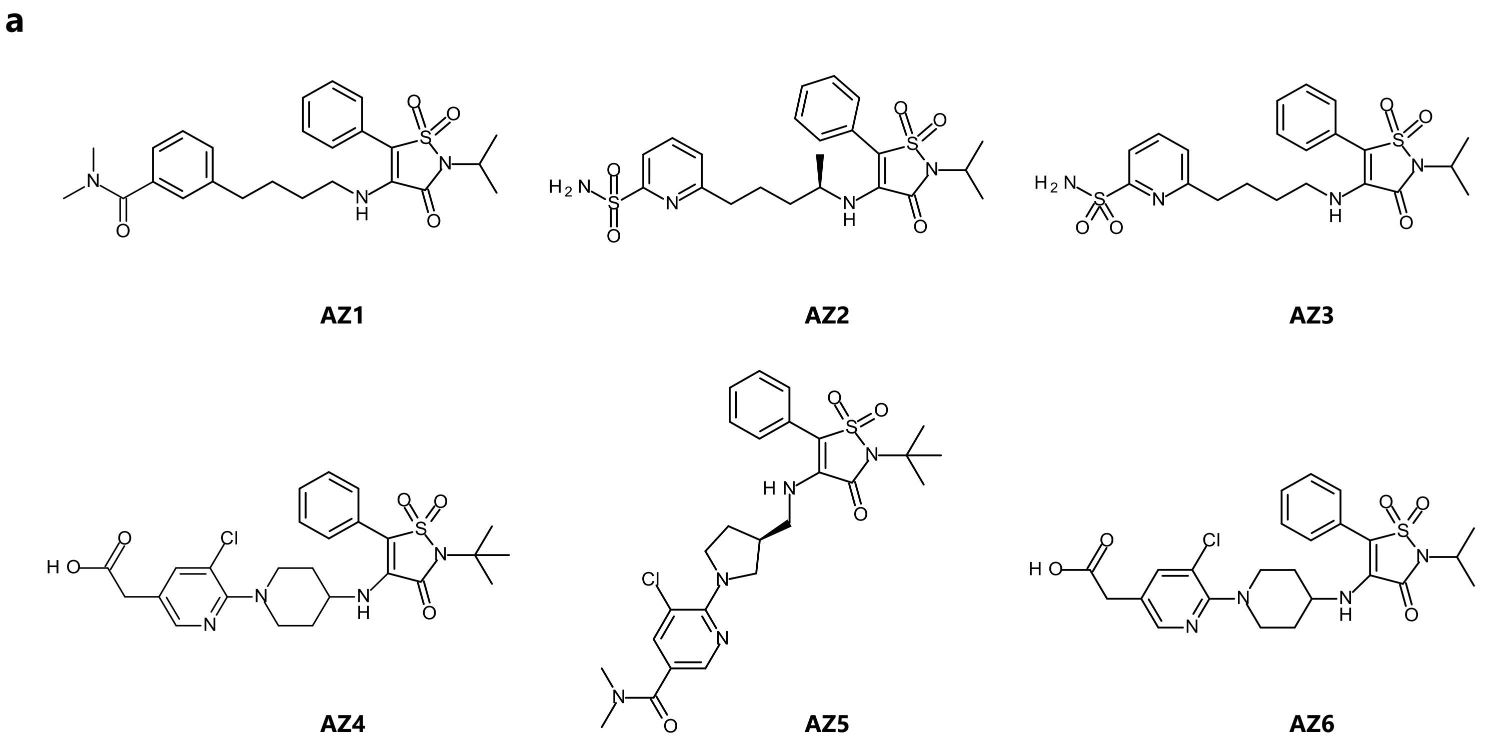

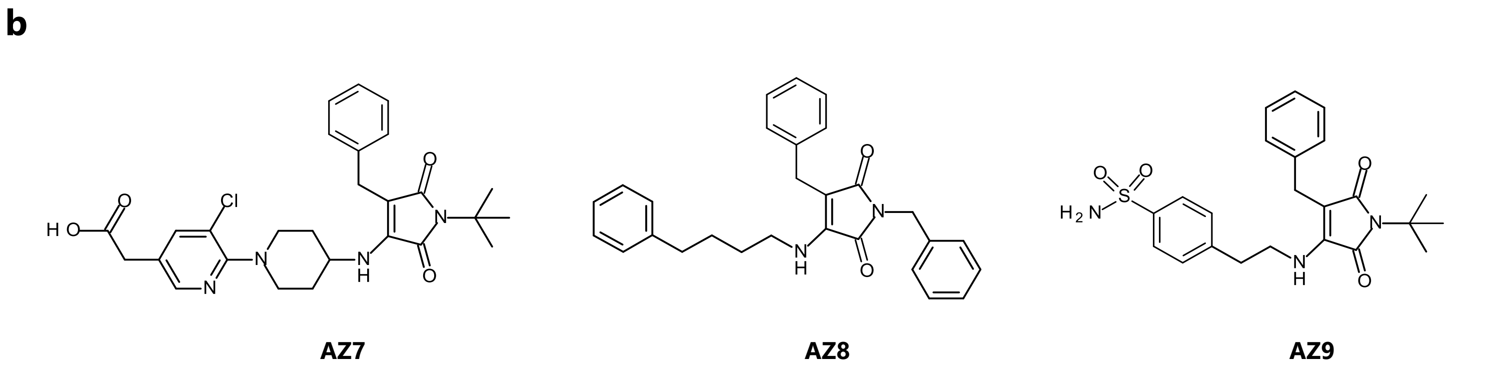

Supplementary Figure 1. Compounds used in this study and structurally related to AZ876 having (a) a central cyclic sulphone amide scaffold or (b) a maleimide core.
Related to Figure 1

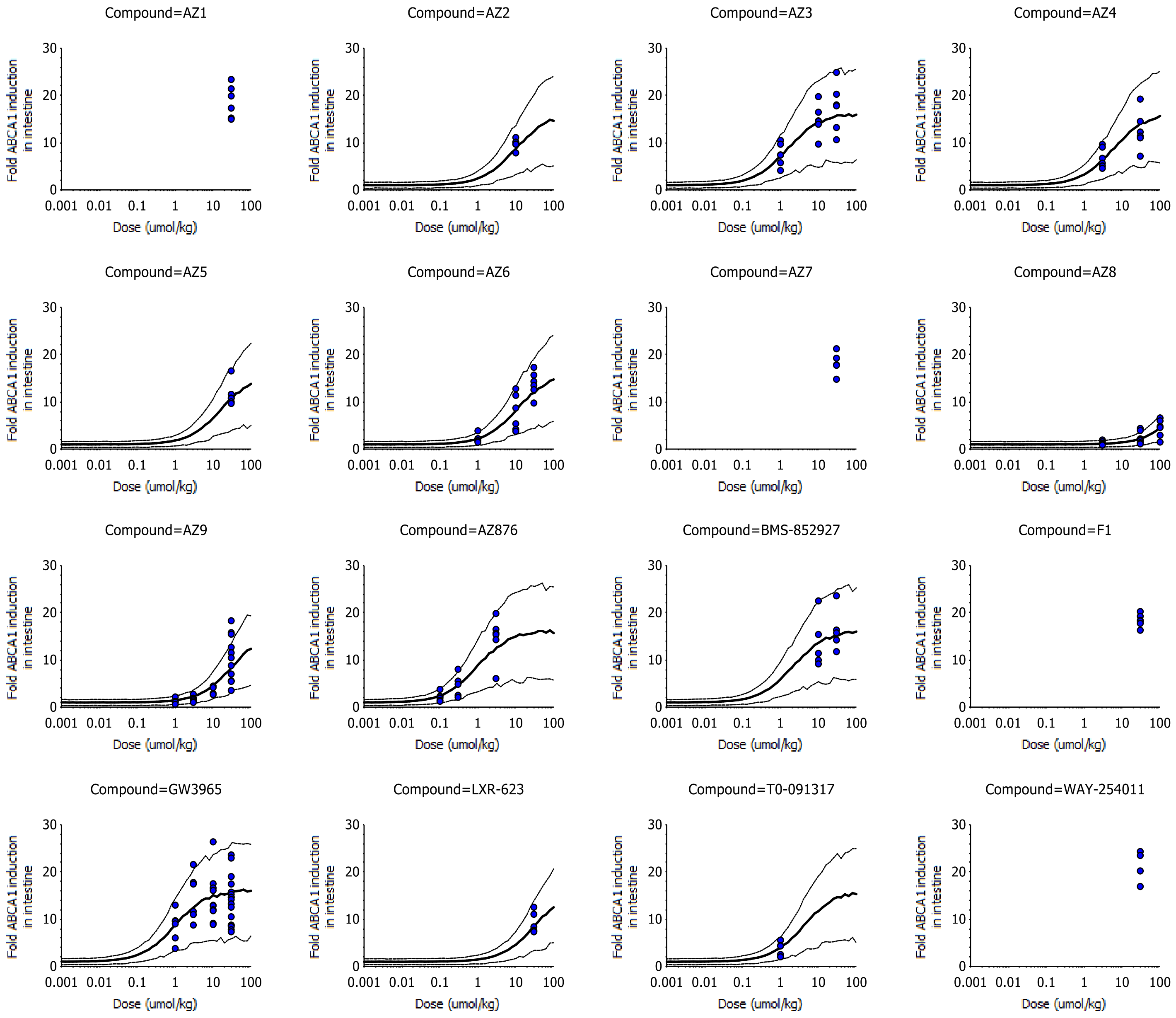

Supplementary Figure 2. Relationship between administered dose and drug induced intestinal *Abca1* mRNA levels. Observed individual fold *Abca1* induction (markers) and model fit (solid line: population mean, dotted lines: 90% confidence interval) of the different drugs included in the assessment. In vivo potency could not be identified for some compounds as no dose-response was available and the observed *Abca1* increase was in the range of the maximal induction (E_{max}).

Related to Table 2

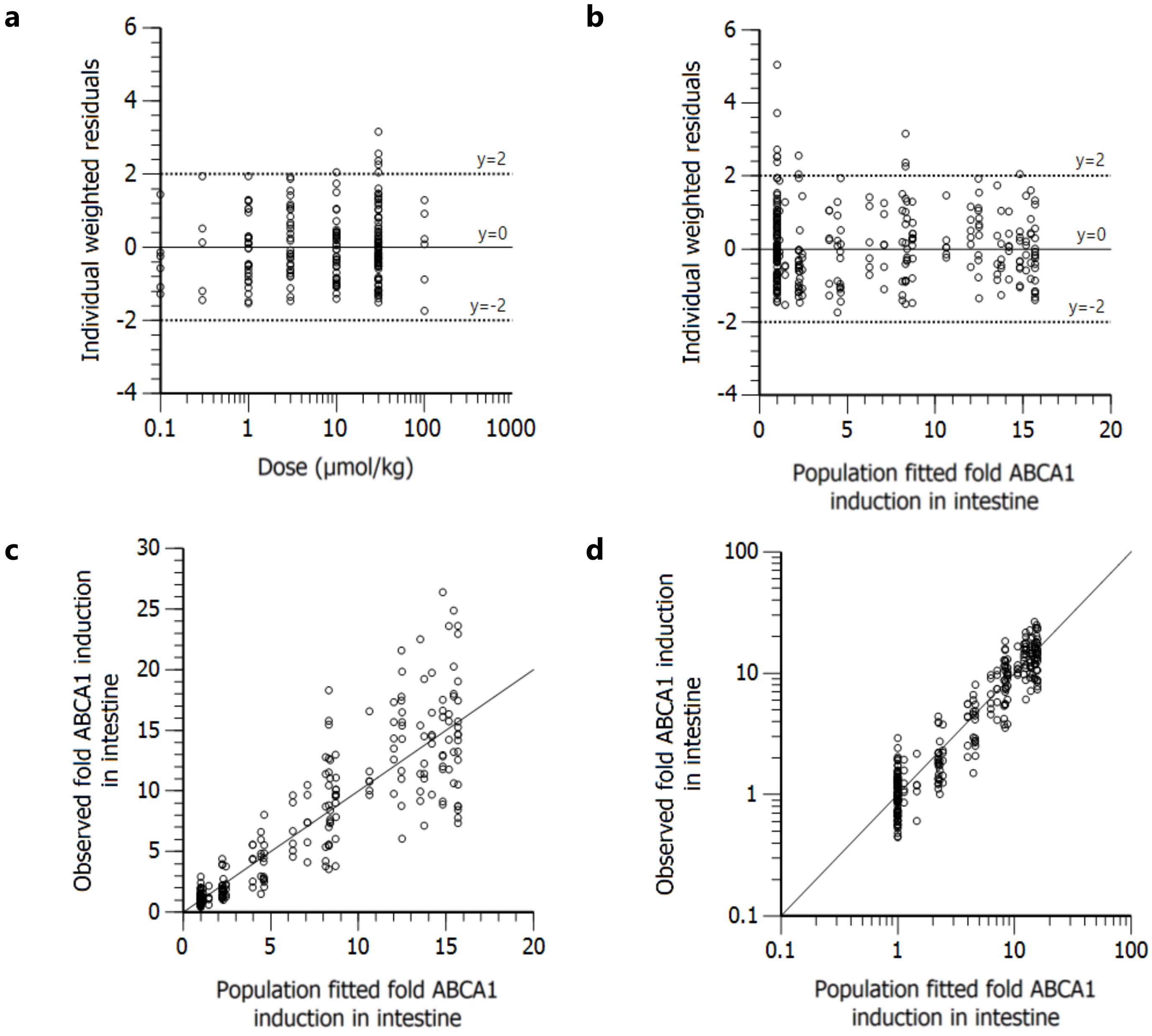

Supplementary Figure 3. Goodness-of-fit plots of the intestinal *Abca1* induction model. Individual weighted residuals of *Abca1* induction versus dose (a) and population fitted fold *Abca1* induction (b). Observed *versus* population fitted fold of *Abca1* induction on a linear (c) and logarithmic scale (d). *Related to Table 2*

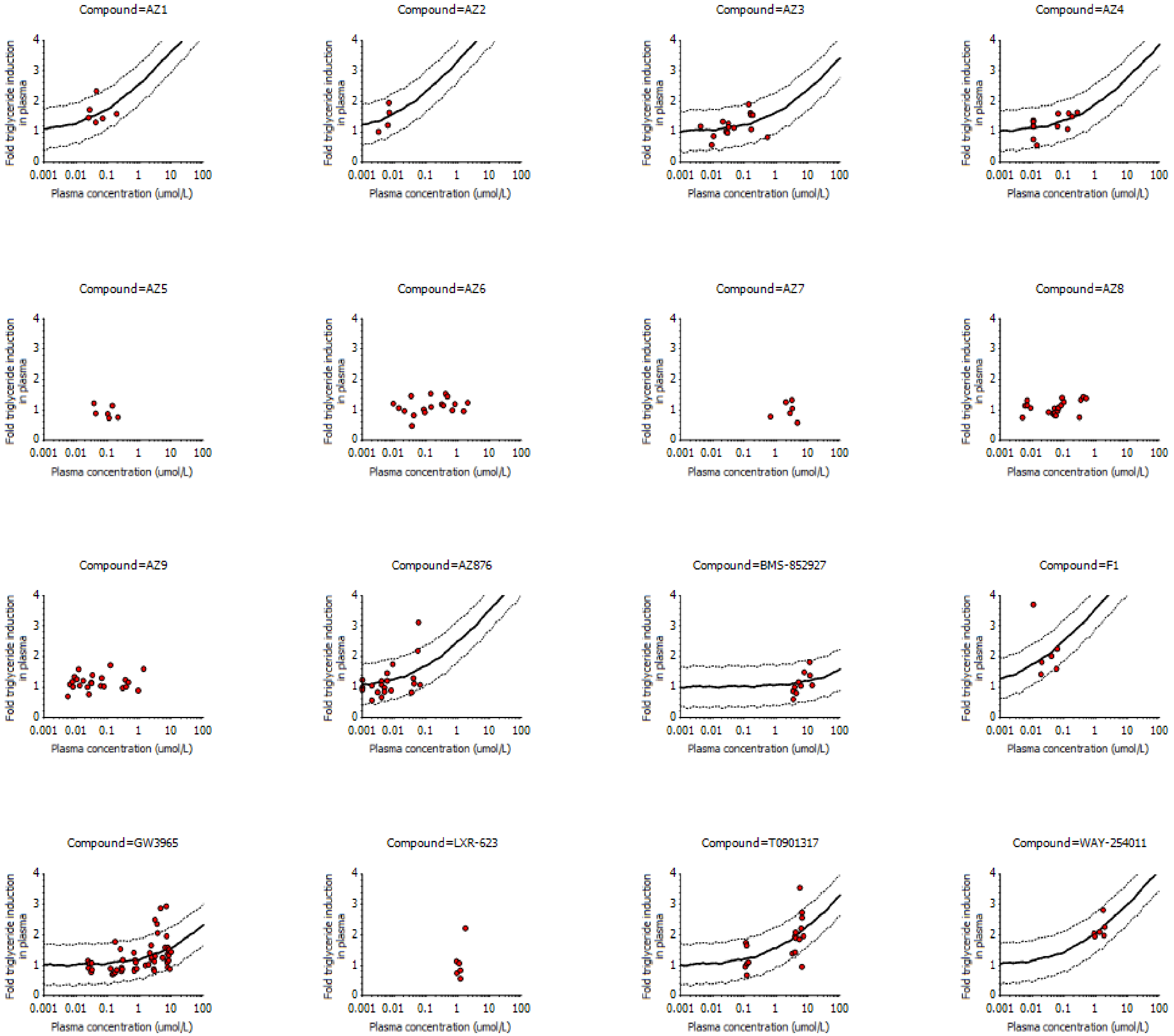

Supplementary Figure 4. Relationship between drug concentration in blood and plasma TG increase. Observed individual fold TG induction (markers) and model fit (solid line: population mean, and dotted lines: 90% confidence interval) of the different drugs included in the assessment. Concentration needed to generate a 2-fold induction of TG levels (C_2) could not be identified for some compounds as no trends in increased TG levels were observed within the explored drug concentration range.

Related to Table 2

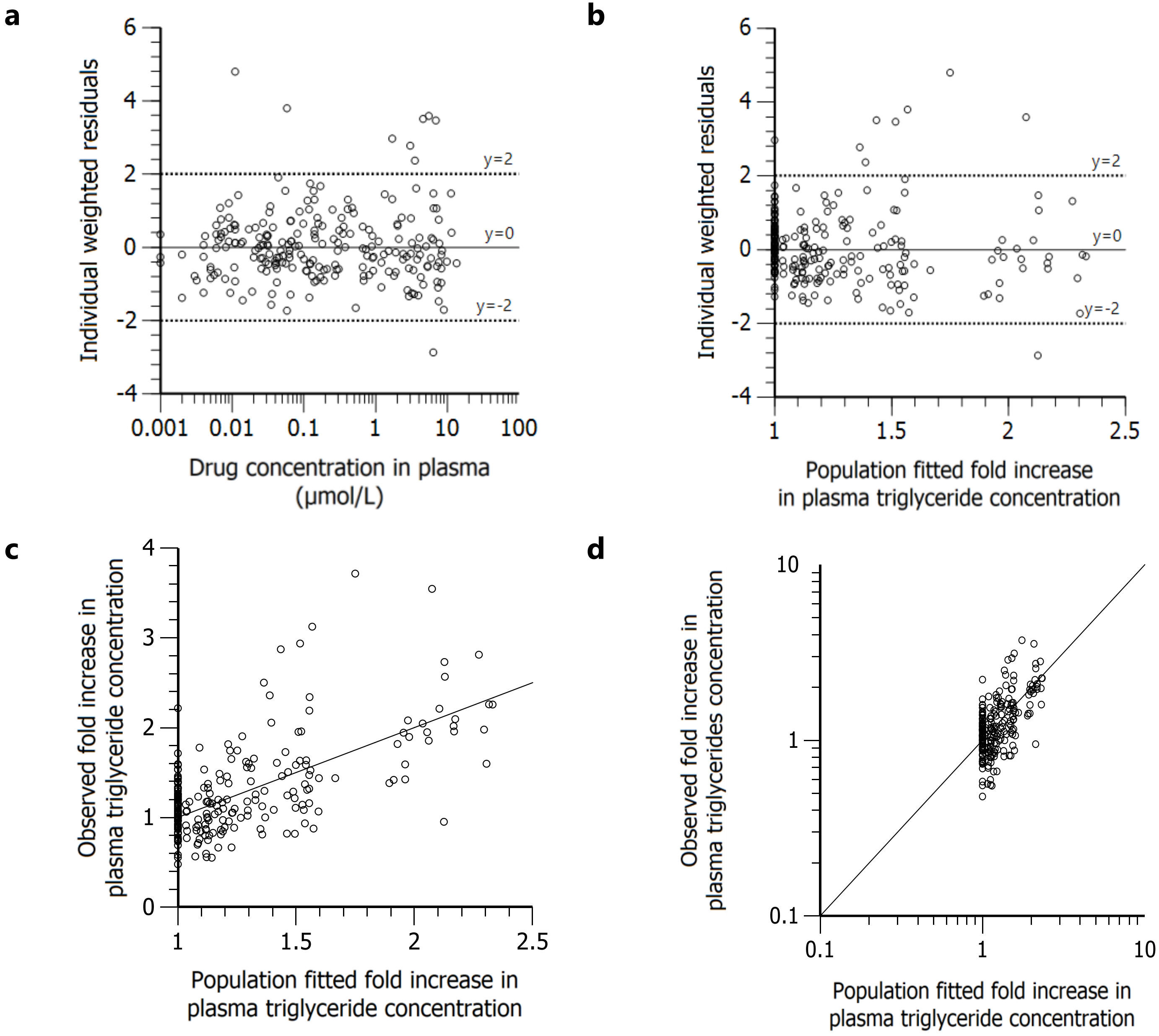

Supplementary Figure 5. Goodness-of-fit plots of the plasma TG model. Individual weighted residuals of TG induction *versus* drug concentration in plasma (a) and population fitted fold increase in plasma TG (b). Observed *versus* population fitted fold increase in plasma TG concertation on a linear (c) and logarithmic scale (d).
Related to Table 2

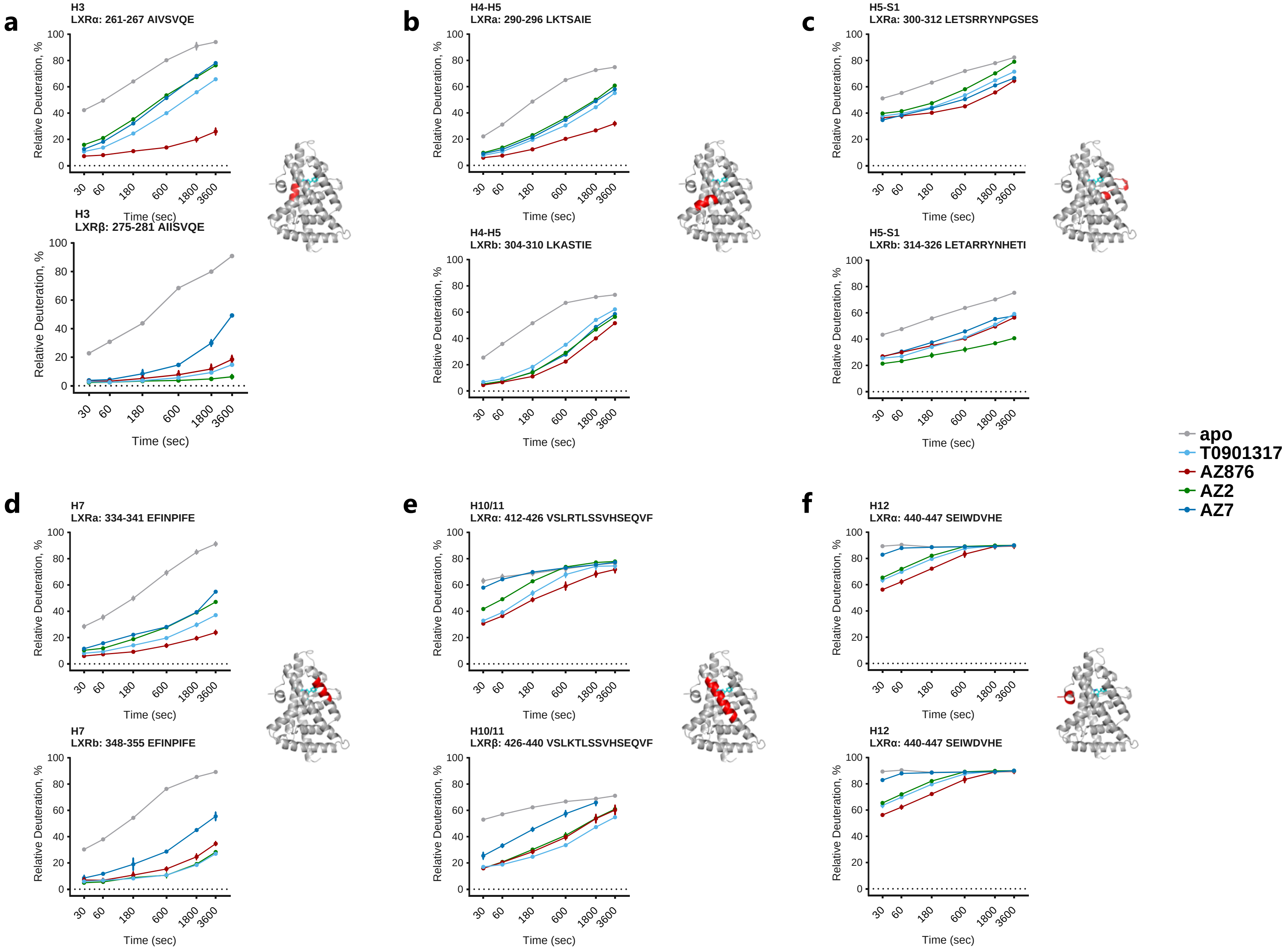

Supplementary Figure 6. HDX-MS kinetics of selected LXR peptides. Time point deuteration kinetics of LXRα and LXRβ LBDs in apo state (grey) and incubated with T0901317 (cyan), AZ876 (red), AZ2 (green) or AZ7 (blue) showing peptides from H3 (a), H4-H5 (b), H5-S1 (c), H7 (d), H10-H11 (e) and H12 (f). Peptide location within LXR LBD is mapped on the LXRβ crystal structure (1PQC.pdb). HDX-MS data were collected in triplicate, and deuteration data are normalized to the fully deuterated control (± standard deviation).

Related to Figure 2

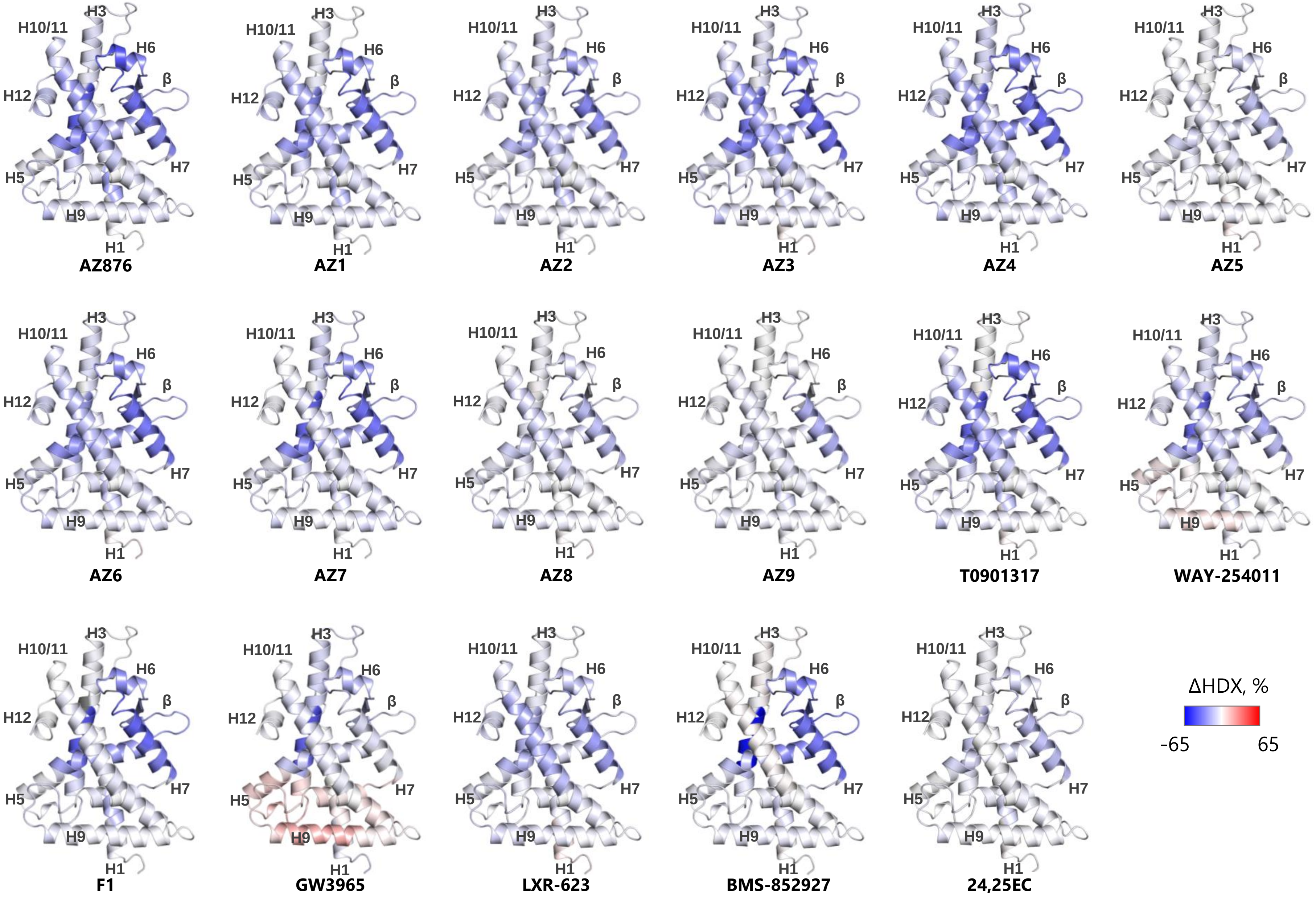

Supplementary Figure 7. Differential HDX-MS analysis of LXRα-ligand interactions. Protection from deuterium exchange upon ligand binding mapped onto LXRα crystal structure (2ACL.pdb), differential HDX levels are the average of two time points (30 and 600 sec). A negative difference value corresponds to a protection from deuterium uptake upon ligand binding, with large absolute values corresponding to a strong effect. Deuterium uptake values for all peptides were corrected to back exchange using the fully deuterated control. HDX-MS measurements were performed in triplicates. *Related to Figure 2*

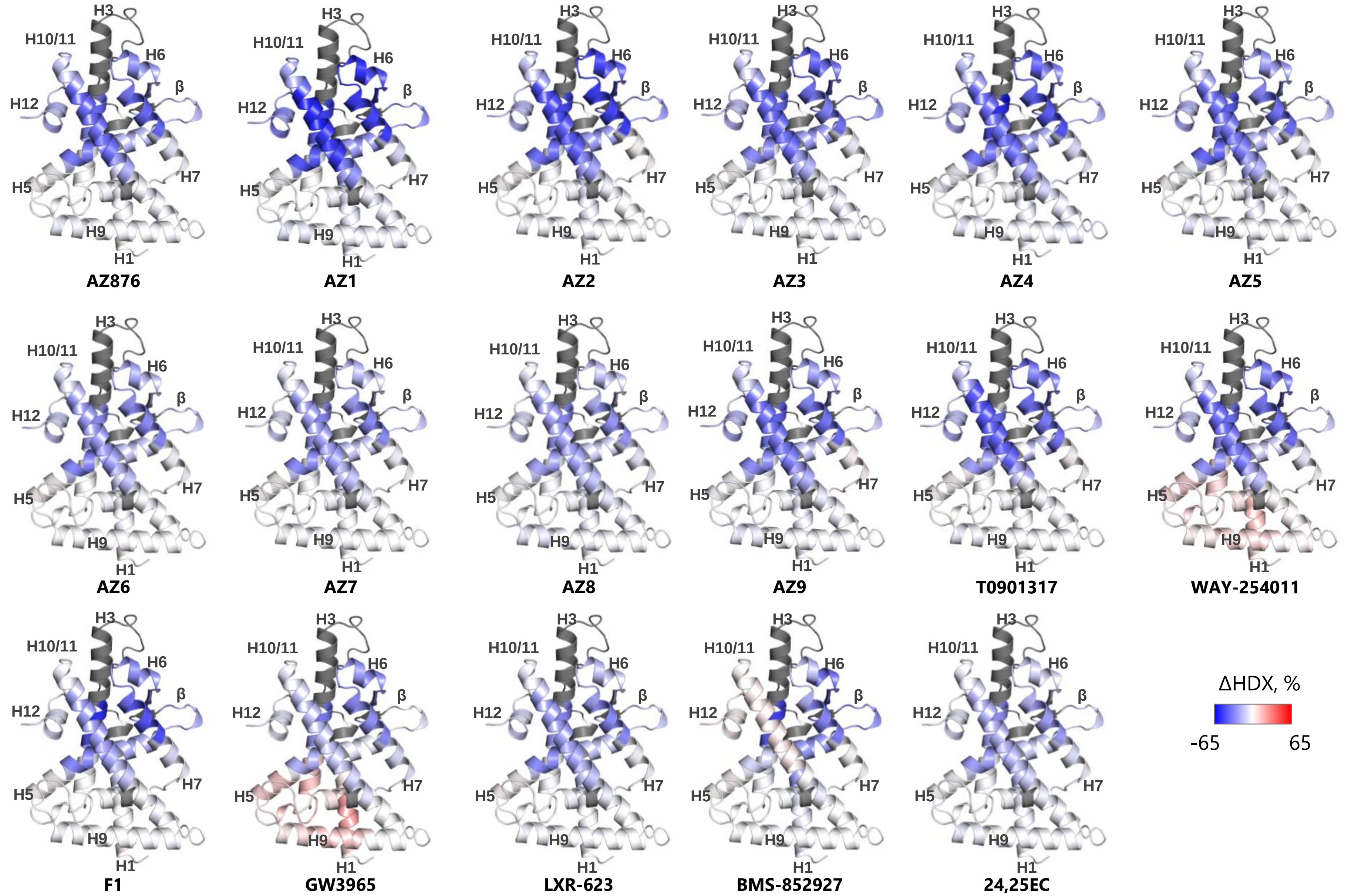

Supplementary Figure 8. Differential HDX-MS analysis of LXRβ-ligand interactions. Protection from deuterium exchange upon ligand binding mapped onto LXRβ crystal structure (1PQC.pdb), differential HDX levels are the average of two time points (30 and 600 sec). A negative difference value corresponds to a protection from deuterium uptake upon ligand binding, with large absolute values corresponding to a strong effect. Deuterium uptake values for all peptides were corrected to back exchange using the fully deuterated control. Regions of the LXRb structure not covered by HDX-MS are shown in dark grey. HDX-MS measurements were performed in triplicates.

Related to Figure 2

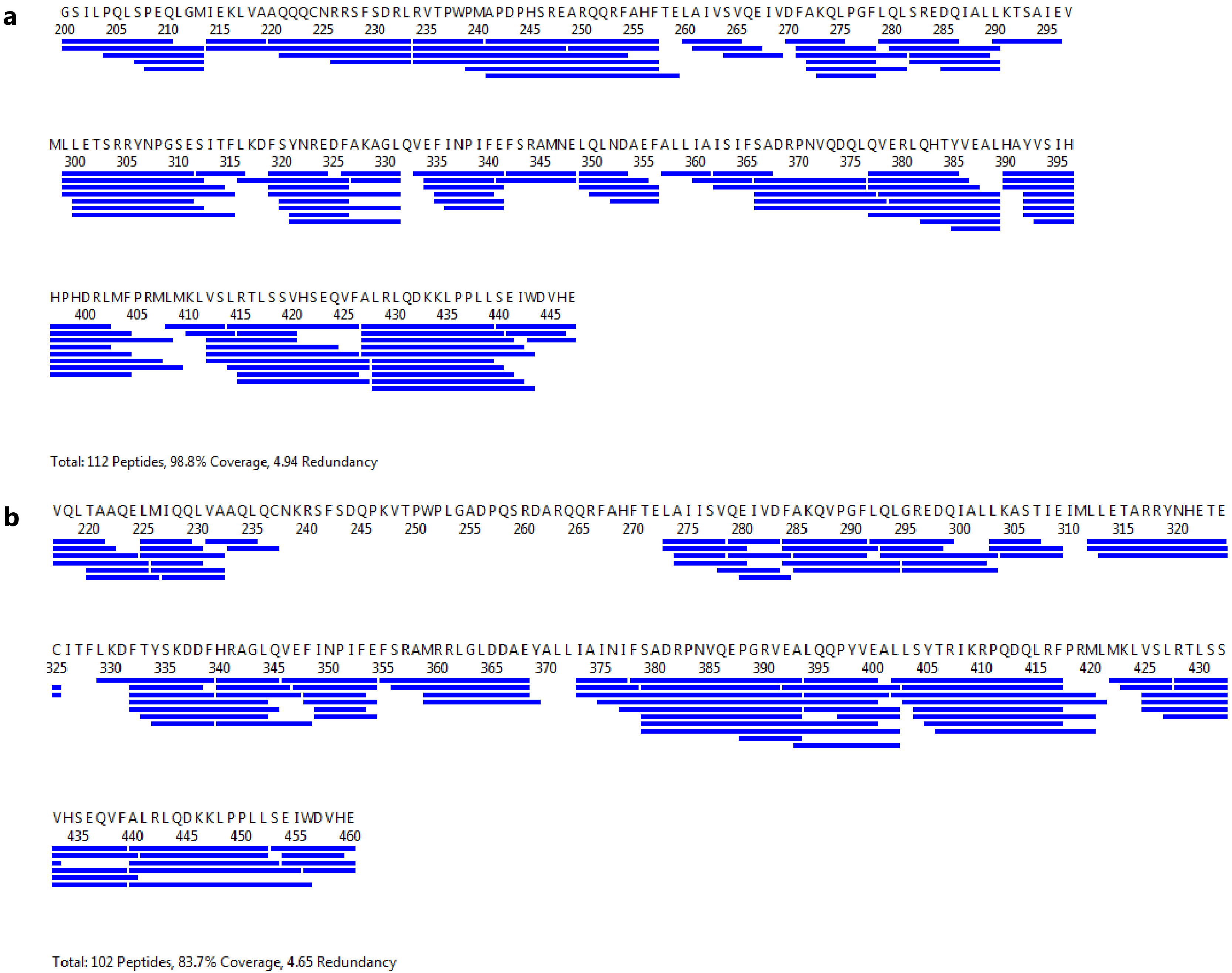

Supplementary Figure 9. Sequence coverage achieved in the HDX-MS experiment**.** The peptides obtained via peptic digestion and LC-MS analysis are shown as blue bars along the protein sequence for LXRα (a) and LXRβ (b) LBDs.

Related to Figure 2

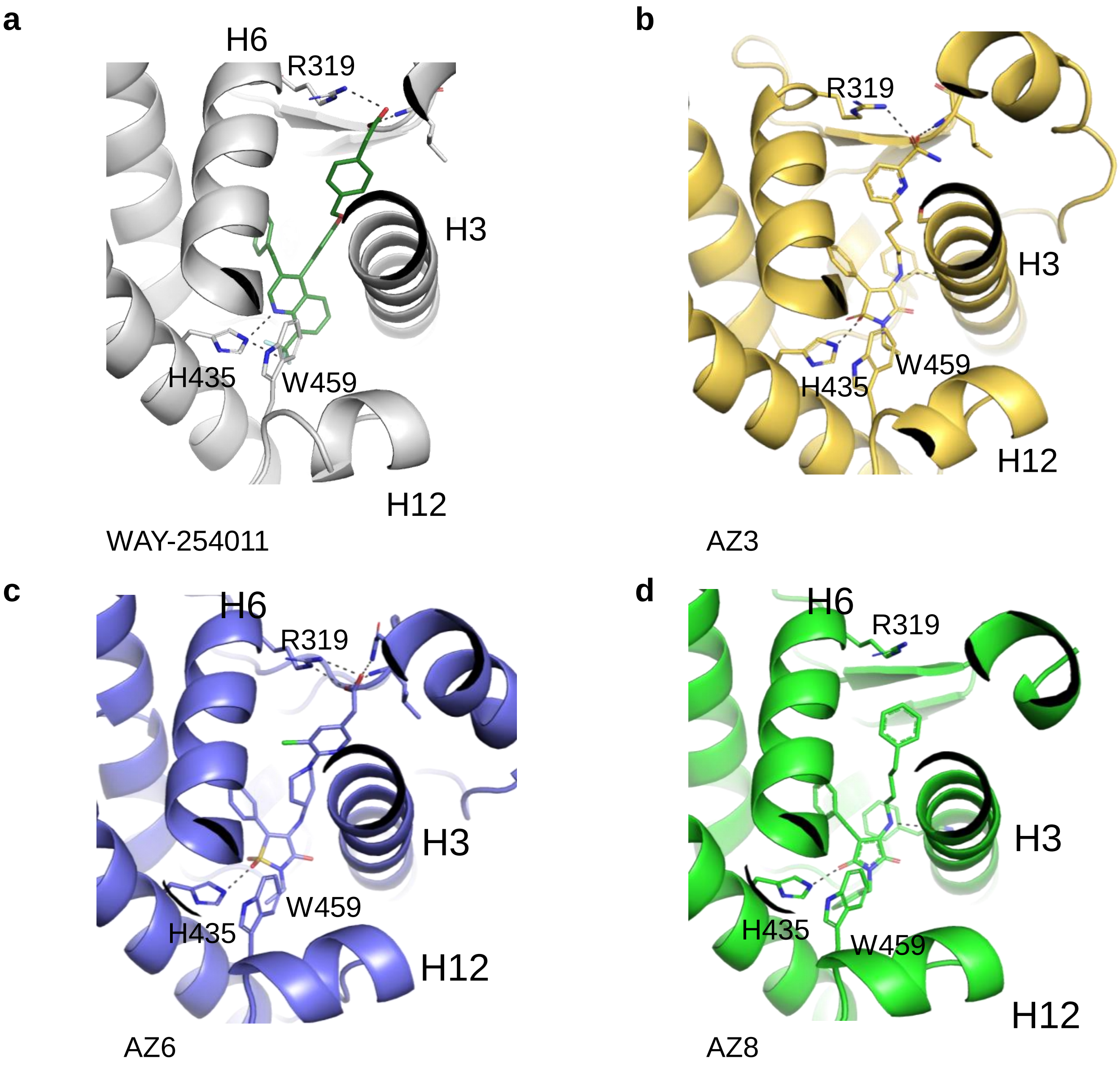

Supplementary Figure 10. Binding modes of WAY-254011 (a), AZ3 (b), AZ6 (c) and AZ8 (d) to LXRβ. In the LXRβ complexes, AZ3, AZ6 and AZ8 bind in a similar manner with the cyclic sulfonamide or maleimide core wedged between H3, H11 and H12, and the sulfone or carbonyl forming a hydrogen bond to His435. The phenyl (AZ3 and AZ6) and benzyl (AZ8) groups fill the pocket between H6 and H8, and form hydrophobic interactions with residues from H8, H6, H7 and H3. The nitrogen in the linker forms a hydrogen bond to the main chain carbonyl of Phe271 in helix H3. The linker is stacking against H3. The polar substituents at the end of the linker are within hydrogen bonding distance to Arg319 and the main chain nitrogen of Leu330.

Related to Figure 2

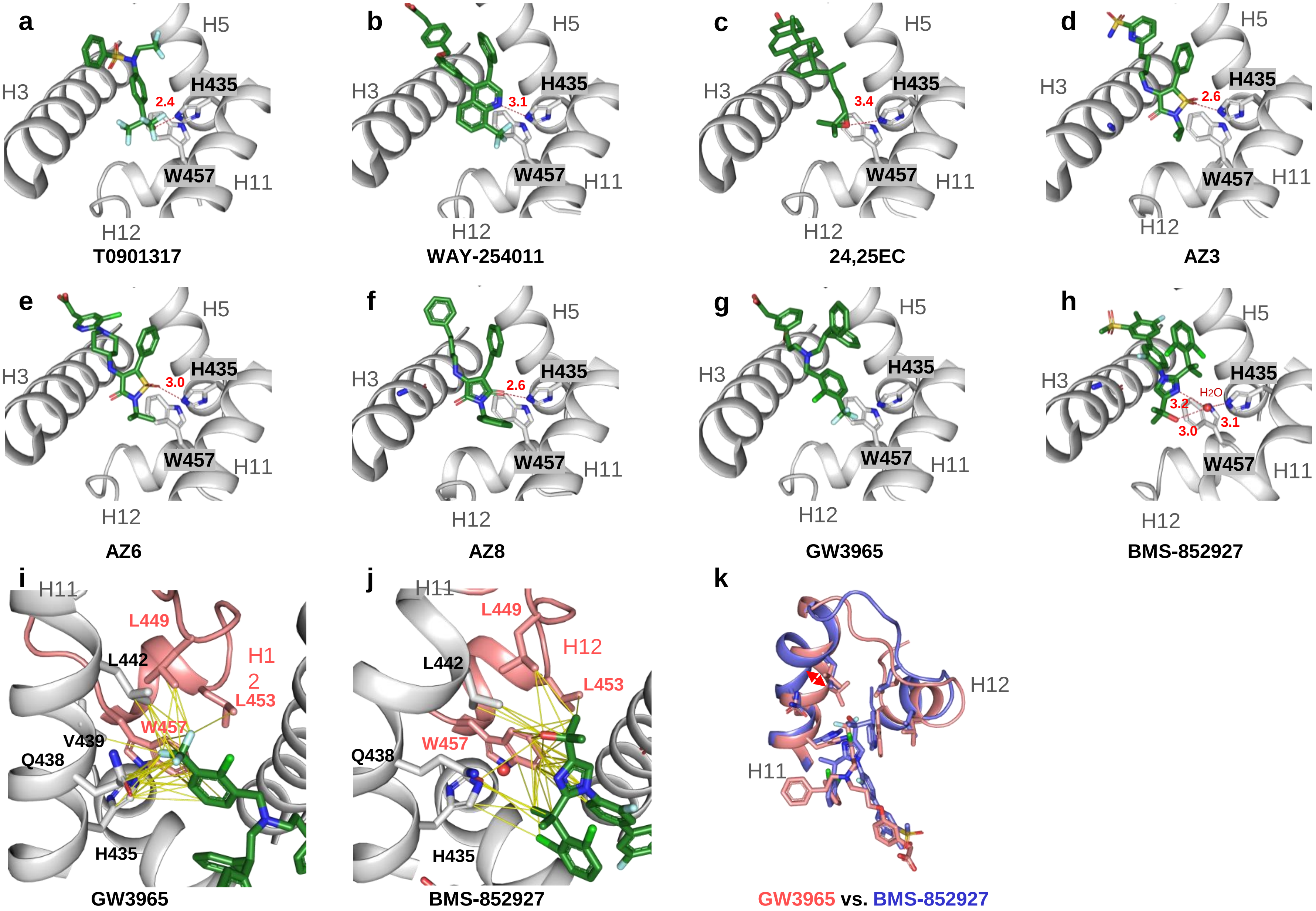

Supplementary Figure 11. Stabilization of the LXRβ His435-Trp457 activation switch by LXR ligands. (a) TO-091317 (PDB ID: 1PQC), (b) WAY-254011, (c) 24,25EC (PDB ID: 1P8D), (d) AZ3, (e) AZ6, (f) AZ8, (g) GW3965 (PDB ID: 1PQ6), and (h) BMS-852927 (PDB ID: 5JY3). Hydrogen bonds are shown in red dash lines. Interactions between GW3965 (i) or BMS-852927 (j) and residues of LXRβ H11-H12 at 4.5 Å cutoff shown in yellow. (k) In BMS-852927/LXRβ complexes H11 is shifted away from the ligand by 3.5 Å which leads to the loss or weakening of the interactions.

Related to Figure 2

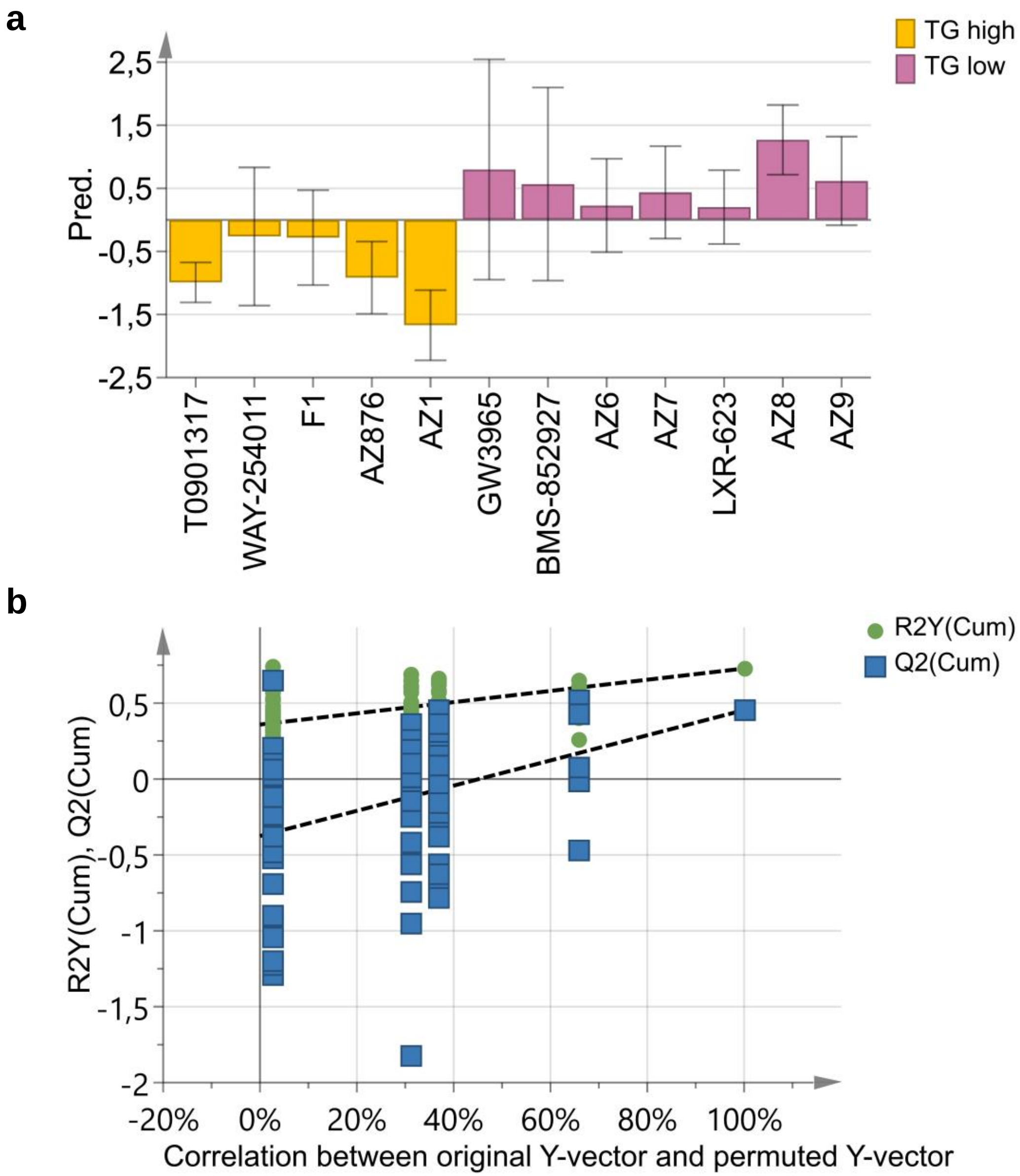

Supplementary Figure 12. OPLS-DA model of TG effects based on LXRα/LXRβ HDX-MS data. OPLS-DA score plot (a) showing the degree of separation of the model between lipogenic (yellow) and non-lipogenic (purple) compounds (N = 12, R2X(cum) = 26.9%, R2Y(cum) = 72.8%, Q2(cum) = 45.4%, P-value = 0,066) and permutation plot of the OPLS-DA model (b). A permutation test performed with 100 random permutations in an OPLS-DA model showing R2 (green circles) and Q2 (blue boxes) values from the permuted analysis (bottom left) significantly lower than the corresponding original values (top right).

Related to Figure 3

a

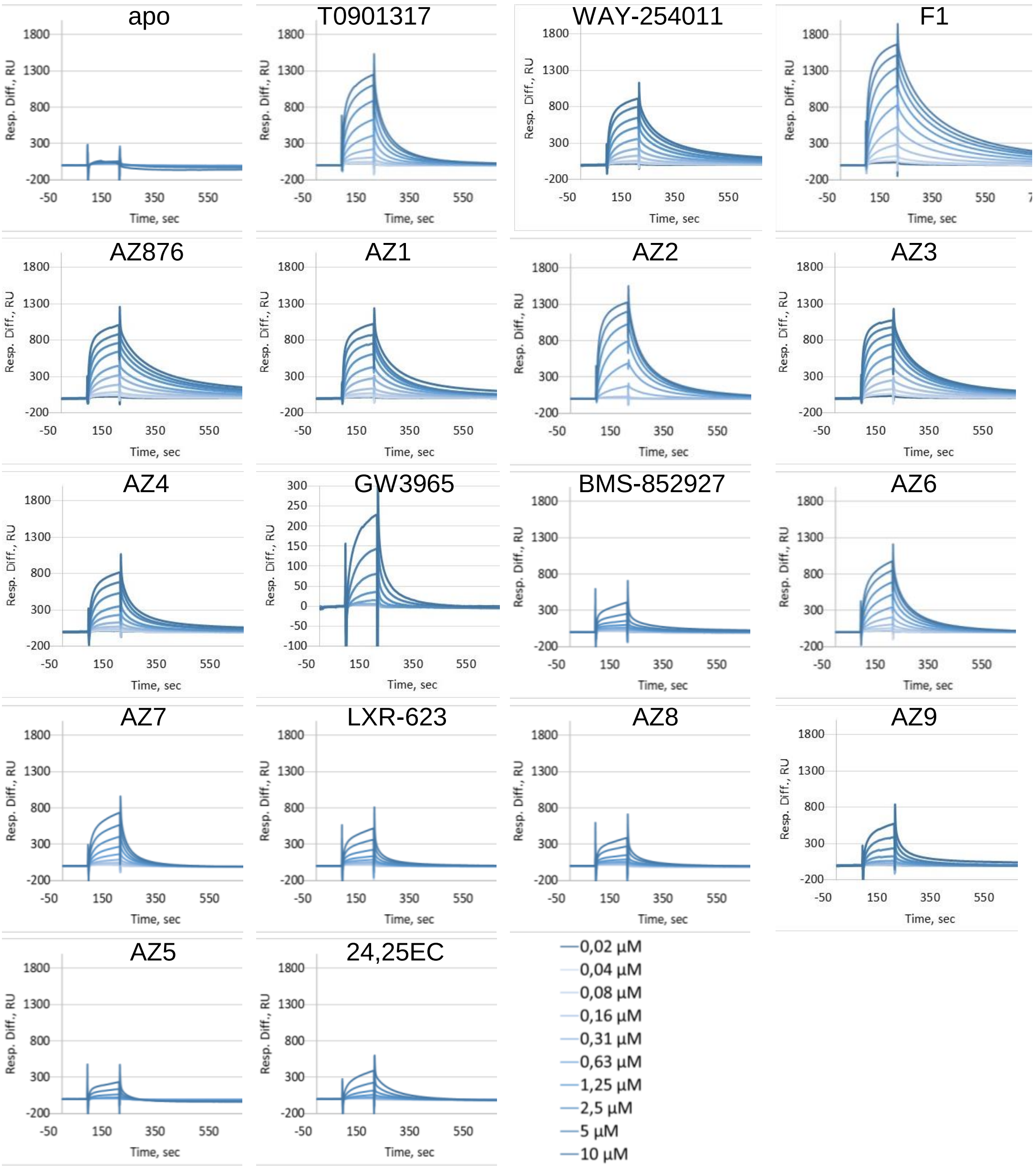

b

LXRα/SRC1	pKd	SE pKd	Kd, µM
**T0901317**	5,823	0,015	1,50
**WAY-254011**	5,875	0,038	1,33
**F1**	6,132	0,023	0,74
**AZ876**	6,036	0,046	0,92
**AZ1**	5,975	0,053	1,06
**AZ2**	6,003	0,031	0,99
**AZ3**	6,21	0,028	0,62
**AZ4**	5,628	0,024	2,35
**GW3965**	5,184	0,055	6,55
**BMS-852927**	4,974	0,069	10,61
**AZ6**	5,823	0,022	1,50
**AZ7**	5,347	0,033	4,49
**LXR-623**	4,667	0,186	21,52
**AZ8**	4,594	0,381	25,47
**AZ9**	5,143	0,029	7,19
**AZ5**	4,96	0,175	10,97
**24,25EC**	5,03	0,075	9,33

Supplementary Figure 13. SPR analysis of ligand-induced LXRα/SRC1 interactions. Obtained response curves (a) are shown in the 0.02 – 10 µM concentration range of LXRα. (b) Equilibrium binding affinity parameters derived from fitting the steady-state region of the sensorgram data to nonlinear regression.

Related to Figure 3

a

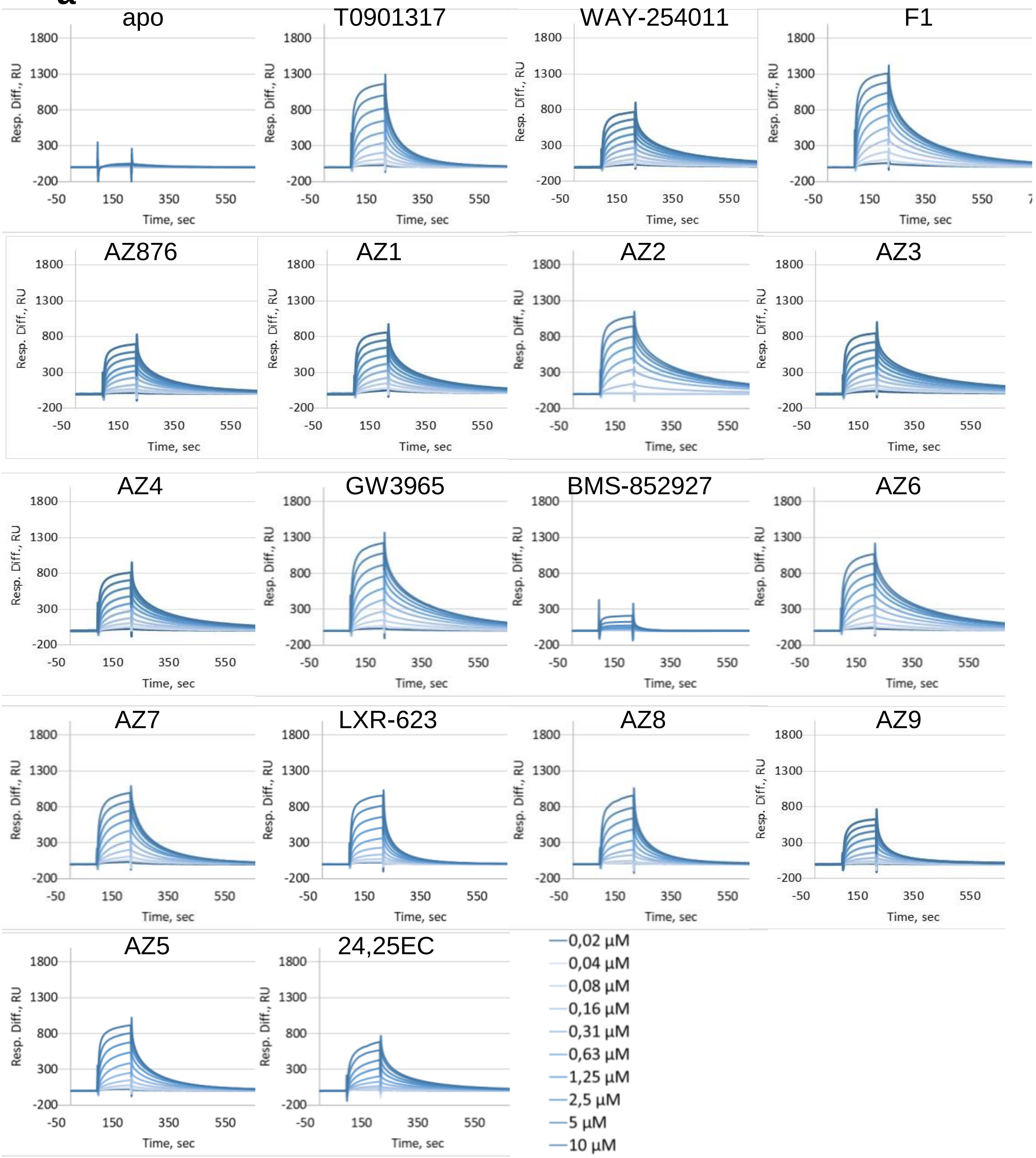

b

LXRβ/SRC1	pKd	SE pKd	Kd, µM
**T0901317**	5,807	0,059	1,56
**WAY-254011**	5,863	0,073	1,37
**F1**	6,205	0,060	0,62
**AZ876**	5,881	0,101	1,32
**AZ1**	5,926	0,079	1,19
**AZ2**	6,063	0,082	0,86
**AZ3**	5,880	0,088	1,32
**AZ4**	5,904	0,069	1,25
**GW3965**	6,012	0,065	0,97
**BMS-852927**	4,704	0,453	19,77
**AZ6**	5,965	0,057	1,08
**AZ7**	5,988	0,046	1,03
**LXR-623**	5,761	0,045	1,73
**AZ8**	5,711	0,062	1,94
**AZ9**	5,923	0,040	1,19
**AZ5**	5,925	0,032	1,19
**24,25EC**	5,596	0,034	2,53

Supplementary Figure 14. SPR analysis of ligand-induced LXRβ/SRC1 interactions**.** Obtained response curves (a) are shown in the 0.02 – 10 µM concentration range of LXRβ. (b) Equilibrium binding affinity parameters derived from fitting the steady-state region of the sensorgram data to nonlinear regression.

Related to Figure 3

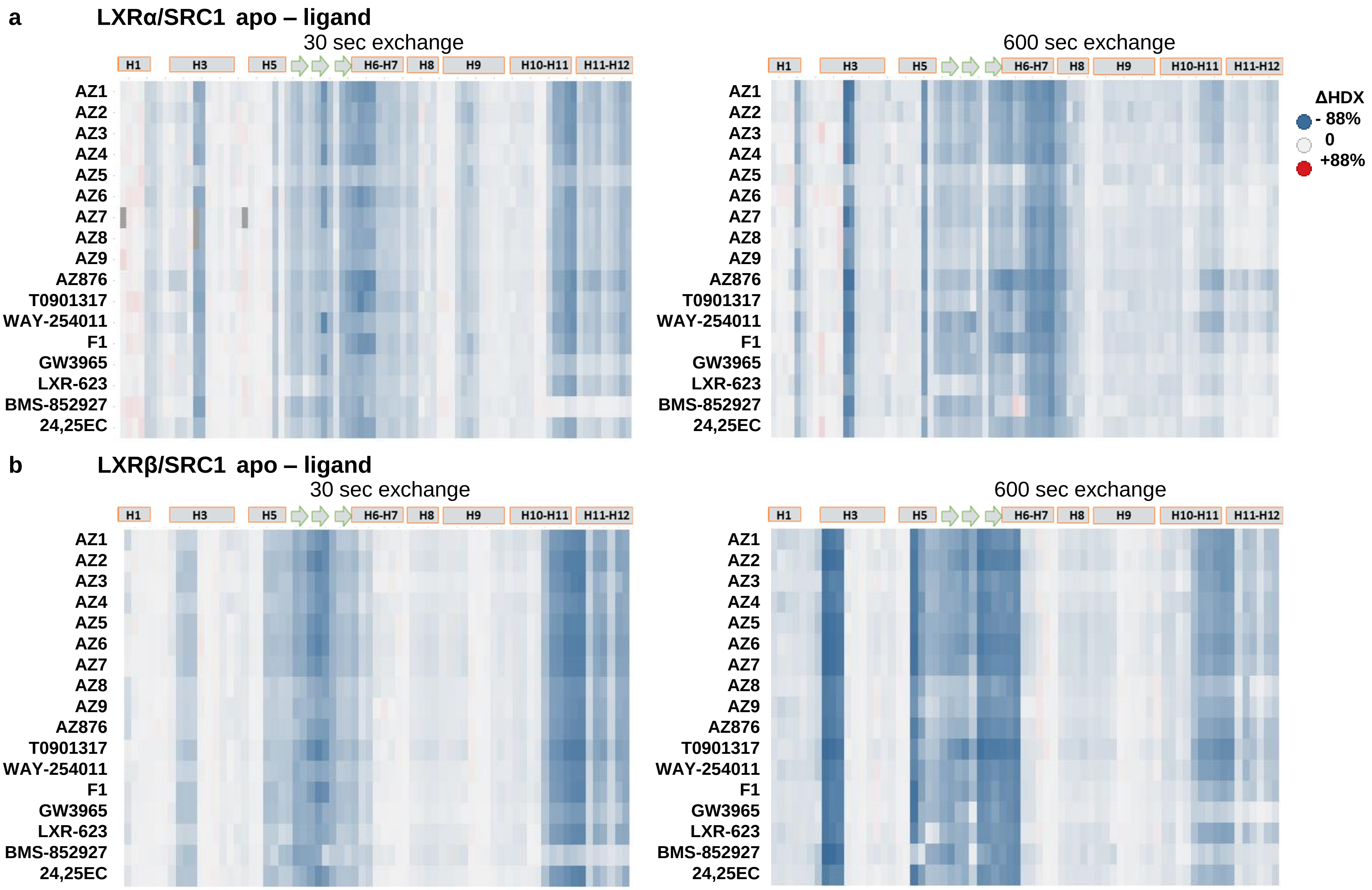

Supplementary Figure 15. Fractional uptake difference heatmap showing the variations of deuterium uptake between apo- and ligand-bound LXRa (a) and LXRb (b) LBD peptides pre-incubated with the SRC1 peptide. Color scheme is identical to Figure 2. Deuterium uptake values for all peptides were corrected to back exchange using the fully deuterated controls. Missing peptides are shown in dark grey. Secondary structure is noted on top of the heatmap. HDX-MS measurements were performed in triplicates and average values are shown. The complete peptide list and numerical values of differences in deuteration between apo and ligand-bound states are shown in the Supplementary Table 4.

Related to Figure 3

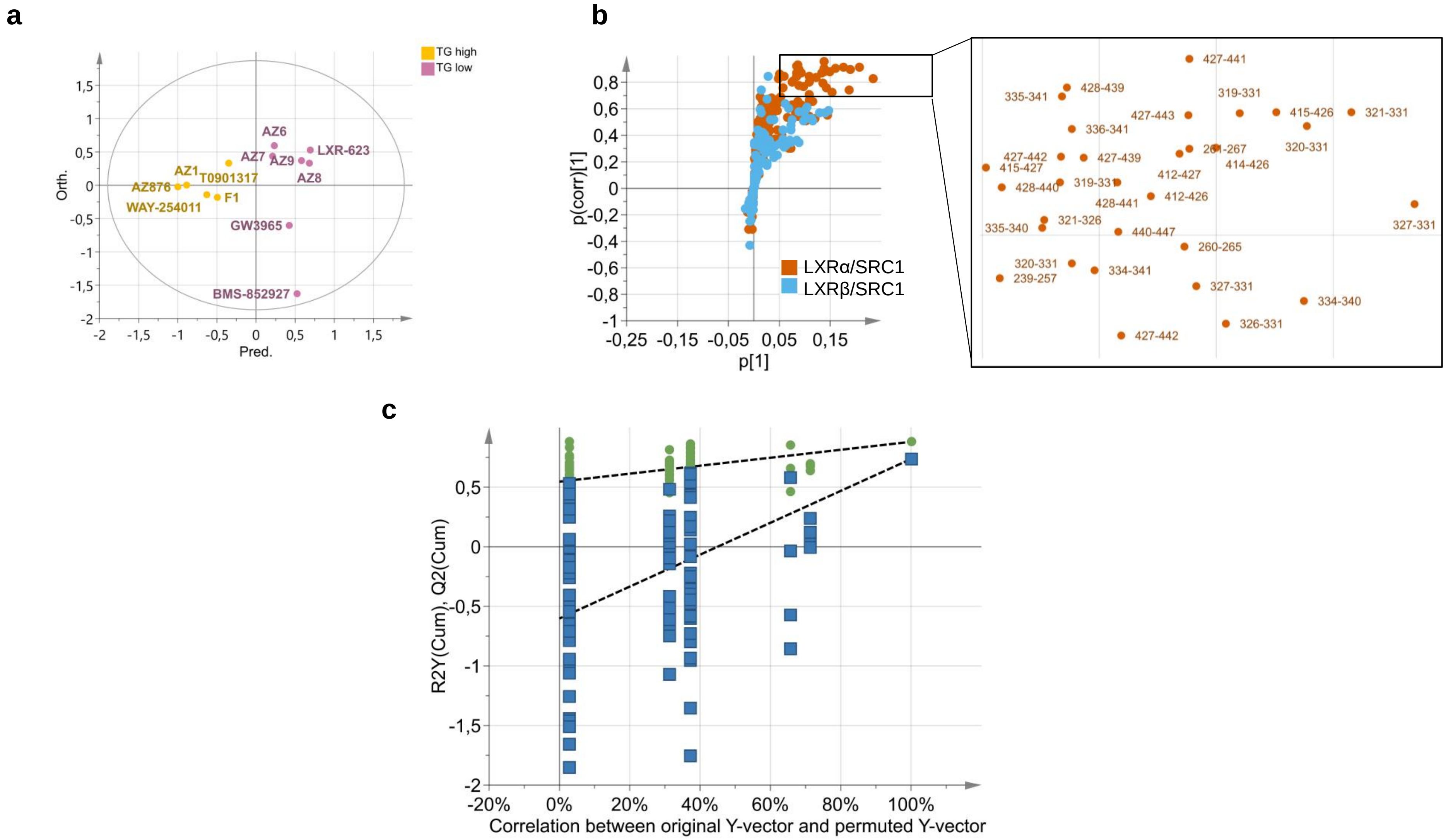

Supplementary Figure 16. OPLS-DA TG model obtained using the LXRα/SRC1 and LXRβ/SRC1 HDX-MS datasets. OPLS-DA score plot (a) showing the degree of separation of the model between lipogenic (yellow) and non-lipogenic (purple) compounds (N = 12, R2X(cum) = 63.8%, R2Y(cum) = 88.1%, Q2(cum) = 73.7%, P-value = 0,033); corresponding S-plot (b) and its zoomed-in part representing limits used for the selection of differential peptides with the highest contribution to class separation (|p(corr)| > 0.7, p > 0.05). Orange dots: LXRα/SRC1 peptides, cyan dots: LXRβ/SRC1 peptides. (c) A permutation test performed with 100 random permutations in an OPLS-DA model showing R2 (green circles) and Q2 (blue boxes) values from the permuted analysis (bottom left) significantly lower than the corresponding original values (top right).

Related to Figure 3

a

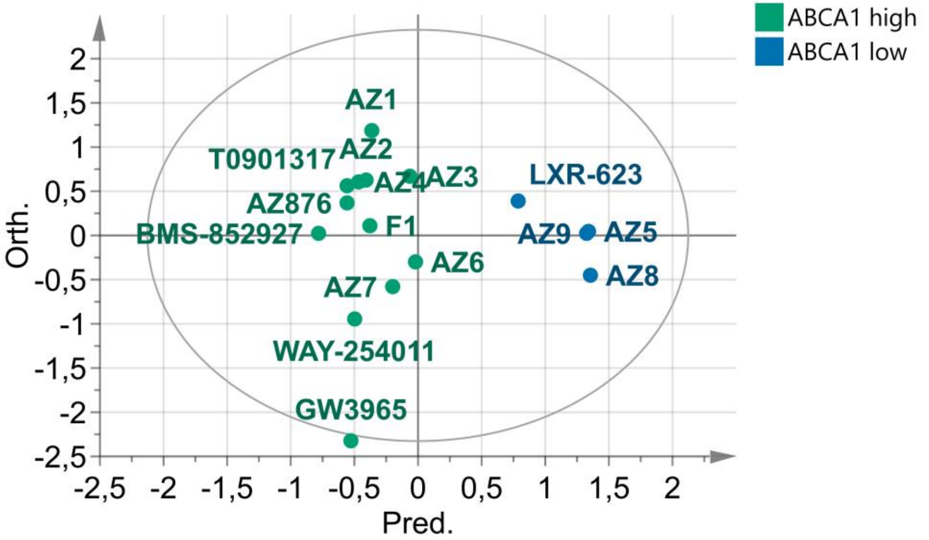

b

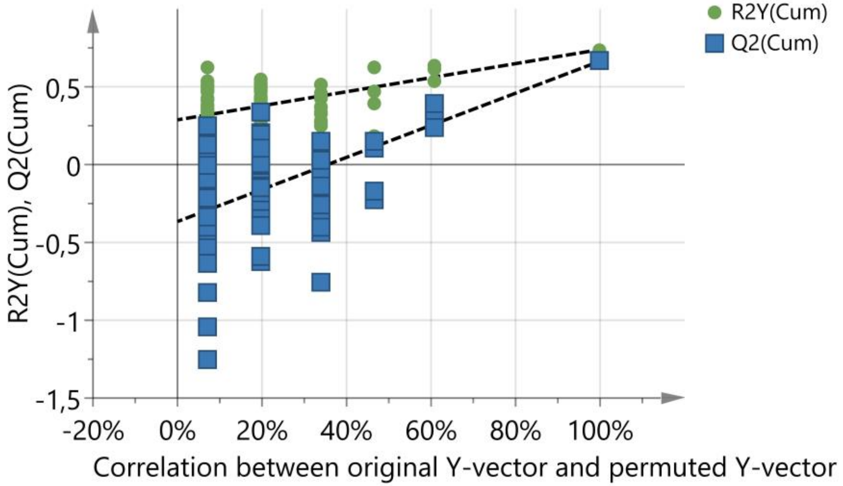

Supplementary Figure 17. OPLS-DA model of ABCA1 effects built using the HDX-MS dataset. OPLS-DA score plot (a) showing the degree of separation of the model between ligands inducing high (green dots) and low (blue dots) levels of intestine *ABCA1* (N = 16, R2X(cum) = 49.9%, R2Y(cum) = 91.1%, Q2(cum) = 76.6%, P-value = 1,75E-03) and permutation plot of the OPLS-DA model (b). A permutation test performed with 100 random permutations in an OPLS-DA model showing R2 (green circles) and Q2 (blue boxes) values from the permuted analysis (bottom left) significantly lower than the corresponding original values (top right).
Related to Figure 4

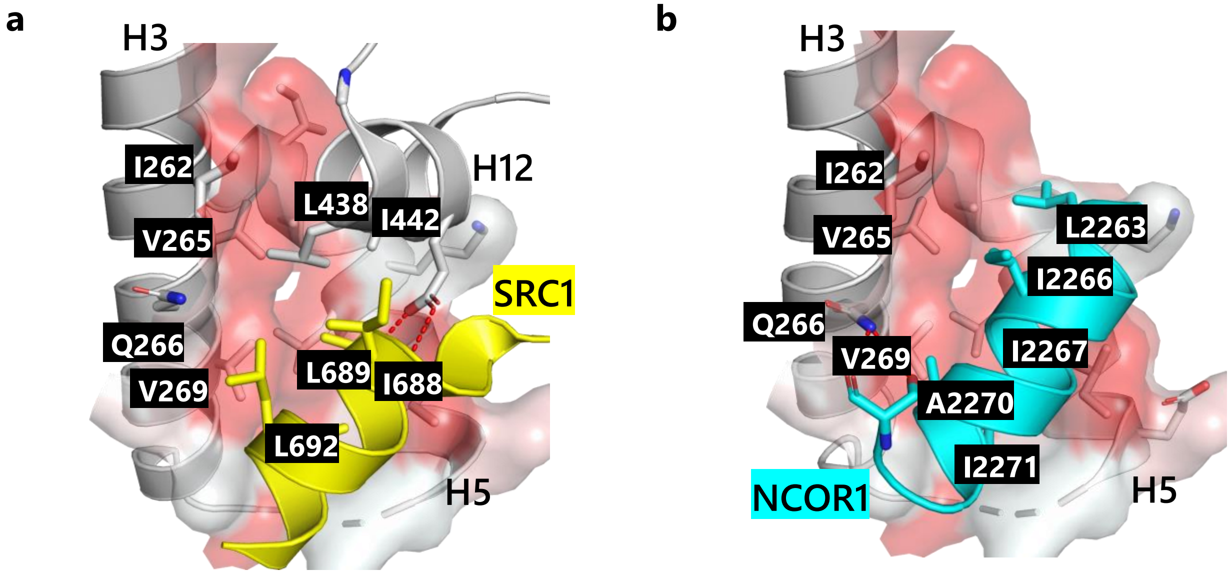

Supplementary Figure 18. Details of the interactions between LXRα and the cofactor peptides. LXRα interacting with the SRC1 peptide (3IPQ.pdb) (a) and the NCOR1-ID2 peptide (b). NCOR1 peptide is from the FXR-NCOR crystal structure (4WVD.pdb) superimposed with the LXRα (3IPQ.pdb). Surfaces created by helices H3 and H5 are colored by the amino acid hydrophobicity scale[1].

1. Eisenberg D. *et al.* Amino acid scale: Normalized consensus hydrophobicity scale. *J. Mol. Biol.* 179, 125-142 (1984).

Related to Figures 4 and 5

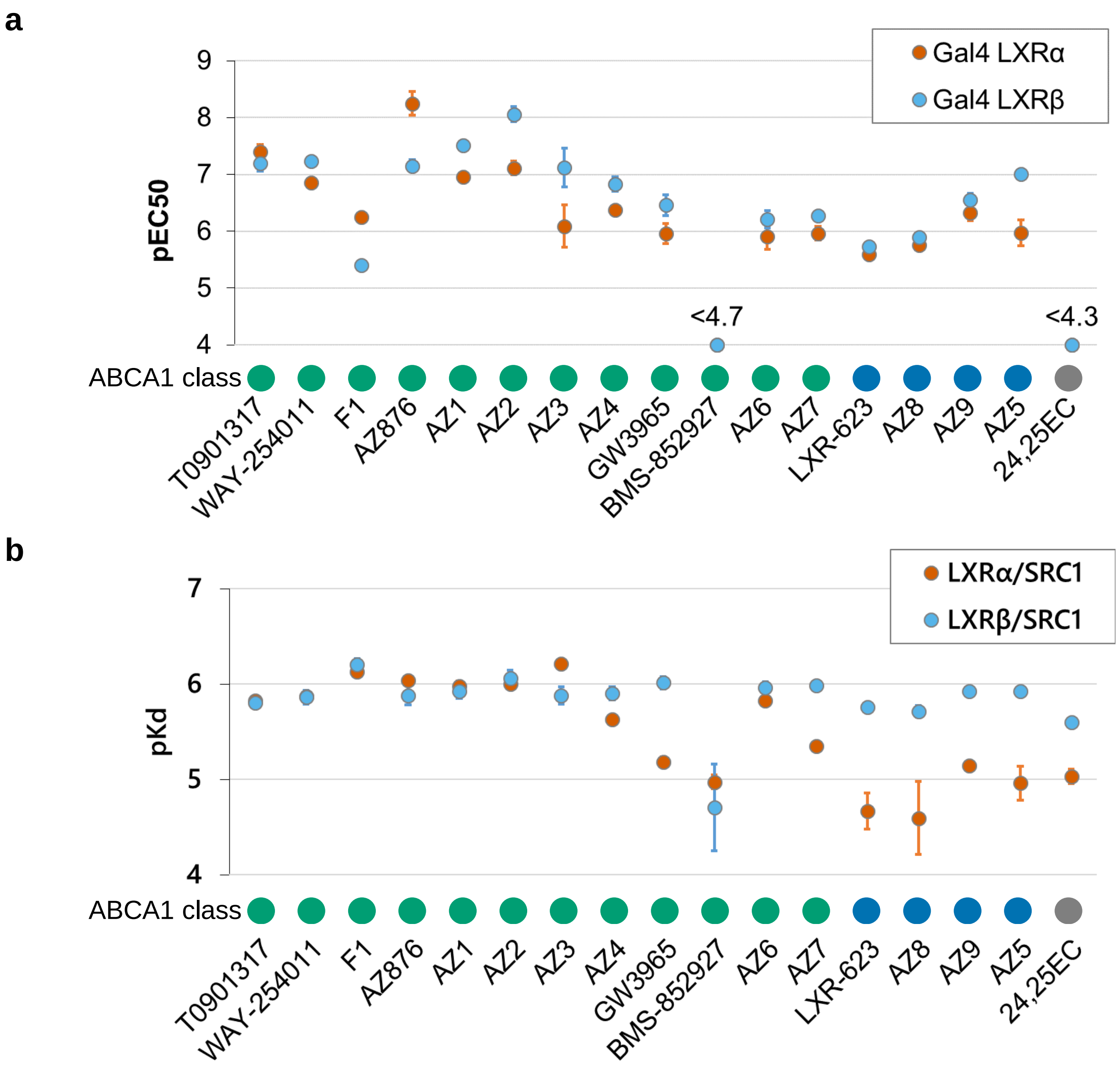

Supplementary Figure 19. *In vitro* reporter gene (a) and coactivator recruitment (b) assays do not predict intestinal *Abca1* induction by LXR compounds. Compound classes based on ABCA1 activity are shown under each graph for clarity: “high ABCA1” ligands in green, “low ABCA1” in blue, compounds with no assigned ABCA1 class in grey. Reporter gene assay: data ± standard deviation (for n values, see Table 1), SRC1 coactivator recruitment assay: data ± standard error.

Related to Figures 4 and 5

a

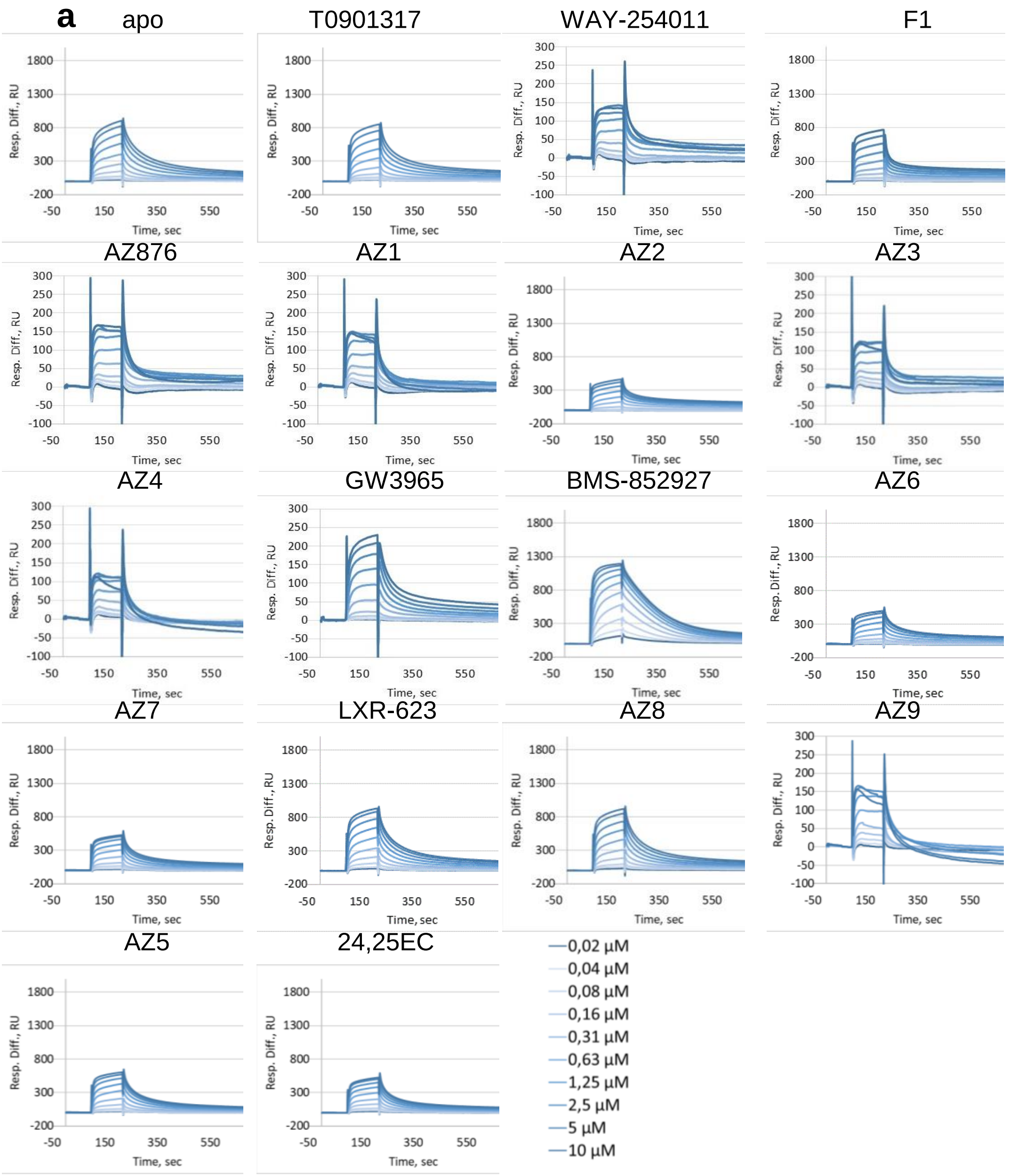

b

**LXRα/NCOR1**	**pKd**	**SE pKd**	**Kd, µM**
**apo**	6,104	0,179	0,79
**T0901317**	5,918	0,013	1,21
**WAY-254011**	6,212	0,049	0,61
**F1**	5,902	0,020	1,25
**AZ876**	6,404	0,027	0,39
**AZ1**	6,370	0,037	0,43
**AZ2**	6,062	0,038	0,87
**AZ3**	6,230	0,036	0,59
**AZ4**	6,369	0,041	0,43
**GW3965**	6,016	0,022	0,96
**BMS-852927**	6,783	0,064	0,16
**AZ6**	6,103	0,009	0,79
**AZ7**	6,276	0,009	0,53
**LXR-623**	6,195	0,012	0,64
**AZ8**	6,117	0,019	0,76
**AZ9**	6,280	0,057	0,52
**AZ5**	6,222	0,011	0,60
**24,25EC**	6,249	0,012	0,56

Supplementary Figure 20. SPR analysis of ligand-modulated LXRα/NCOR1 interactions. Obtained response curves (a) are shown in the 0.02 – 10 µM concentration range of LXRα. (b) Equilibrium binding affinity parameters derived from fitting the steady-state region of the sensorgram data to nonlinear regression.

Related to Figure 5

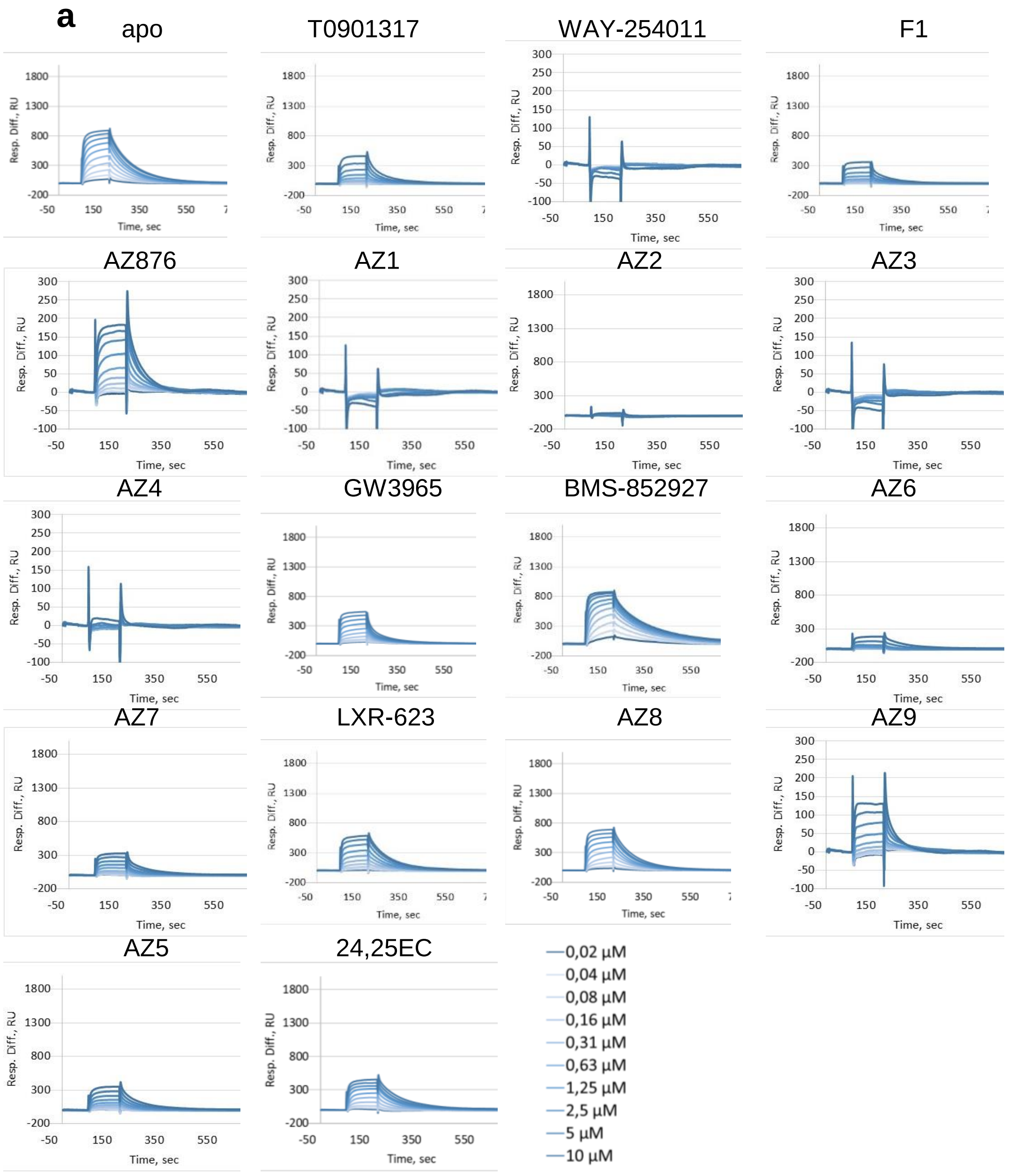

b

LXRα/NCOR1	pKd	SE pKd	Kd, µM
**apo**	6,324	0,166	0,47
**T0901317**	5,040	0,095	9,12
**WAY-254011**	-	-	-
**F1**	5,122	0,032	7,55
**AZ876**	5,961	0,036	1,09
**AZ1**	-	-	-
**AZ2**	-	-	-
**AZ3**	-	-	-
**AZ4**	-	-	-
**GW3965**	5,977	0,029	1,05
**BMS-852927**	6,828	0,103	0,15
**AZ6**	4,623	0,163	23,82
**AZ7**	5,640	0,055	2,29
**LXR-623**	5,903	0,032	1,25
**AZ8**	6,261	0,049	0,55
**AZ9**	5,645	0,085	2,26
**AZ5**	5,296	0,107	5,06
**24,25EC**	6,251	0,049	0,56

Supplementary Figure 21. SPR analysis of ligand-modulated LXRβ/NCOR1 interactions. Obtained response curves (a) are shown in the 0.02 – 10 µM concentration range of LXRβ. (b) Equilibrium binding affinity parameters derived from fitting the steady-state region of the sensorgram data to nonlinear regression.

Related to Figure 5

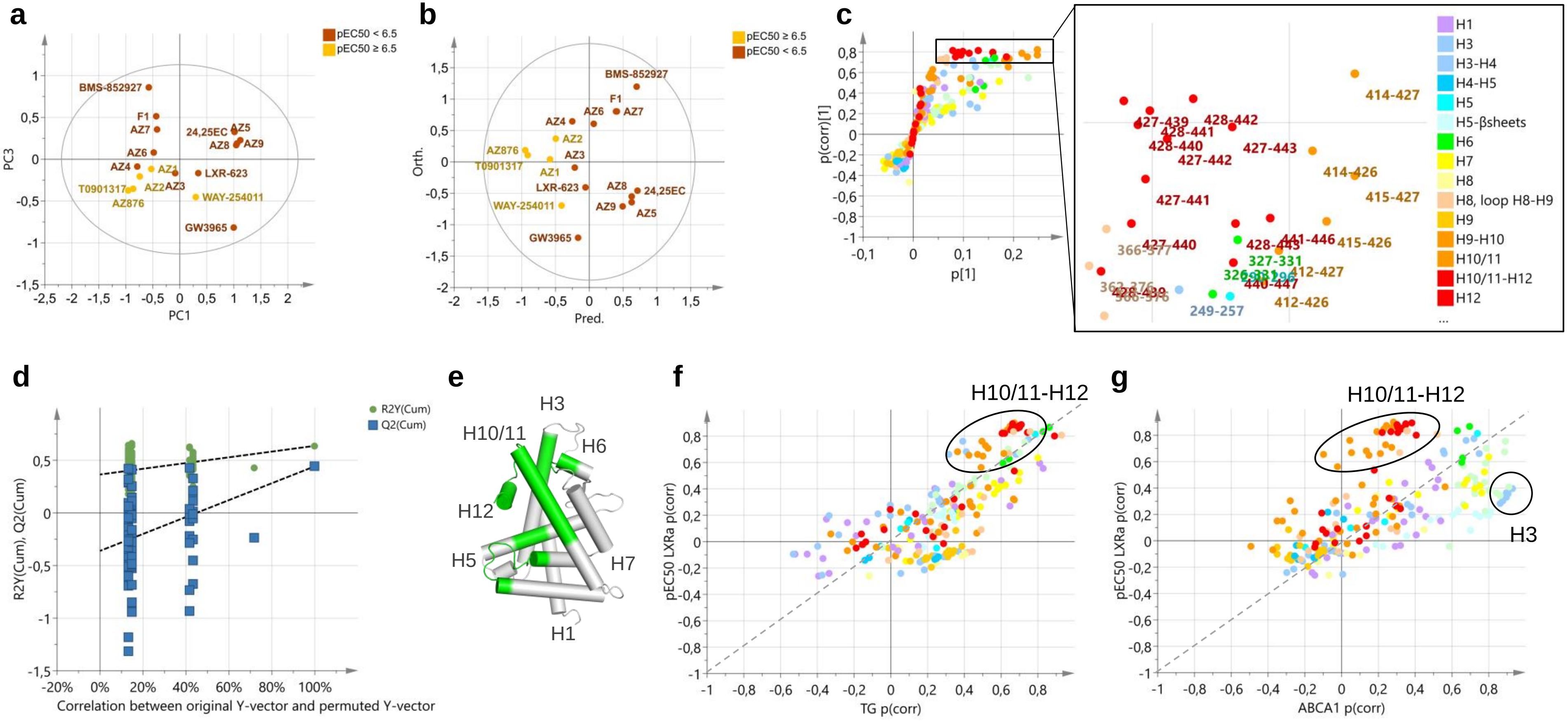

Supplementary Figure 22. OPLS-DA model for Gal4 LXRα pEC50 based on the LXRα HDX-MS dataset. PCA (a) and OPLS-DA score plot (b) showing the degree of separation between compounds with pEC50 < 6.5 (brown) and pEC50 ≥ 6.5 (yellow) derived from in vitro LXRα reporter gene assay (N = 17, R2X(cum) = 61.4%, R2Y(cum) = 63.7%, Q2(cum) = 44.3%, P-value = 0,109861); corresponding S-plot (c) and its zoomed-in part representing limits used for the selection of differential peptides with the highest contribution to class separation (|p(corr)| > 0.7, p > 0.05). Peptides are colored by the corresponding LXRα secondary structure elements. (d) A permutation test performed with 100 random permutations in an OPLS-DA model showing R2 (green circles) and Q2 (blue boxes) values from the permuted analysis (bottom left) significantly lower than the corresponding original values (top right). (e) Significant peptides (|p(corr)| > 0.7, p > 0.05) mapped on the LXRα LBD crystal structure (2ACL.pdb). SUS plots of the Gal4 LXRα pEC50 vs. TG (f) and ABCA1 (g) OPLS-DA models showing that the pEC50 and TG but not ABCA1 models have peptides of equally high importance clustering along the diagonal. For clarity, peptides with low coefficients of contribution to the class separation for both models (p < 0.05) were excluded.

Related to Figure 6

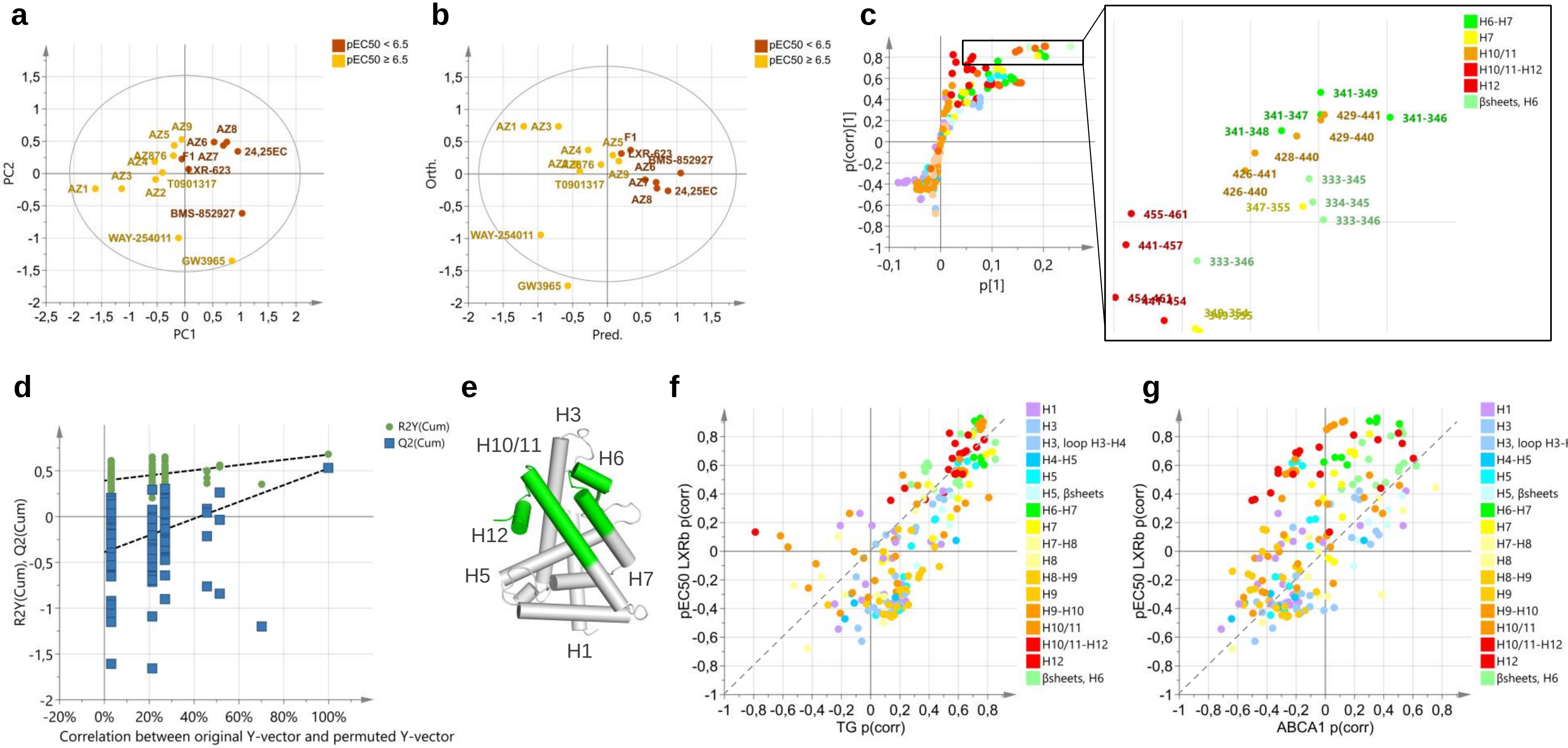

Supplementary Figure 23. OPLS-DA model for Gal4 LXRβ pEC50 based on the LXRβ HDX-MS dataset. PCA (a) and OPLS-DA score plot (b) showing the degree of separation between compounds with pEC50 < 6.5 (brown) and pEC50 ≥ 6.5 (yellow) derived from in vitro LXRβ reporter gene assay (N = 17, R2X(cum) = 63.5%, R2Y(cum) = 67.7%, Q2(cum) = 52.7%, P-value = 0,0464174); corresponding S-plot (c) and its zoomed-in part representing limits used for the selection of differential peptides with the highest contribution to class separation (|p(corr)| > 0.7, p > 0.05). Peptides are colored by the corresponding LXRβ secondary structure elements. (d) A permutation test performed with 100 random permutations in an OPLS-DA model showing R2 (green circles) and Q2 (blue boxes) values from the permuted analysis (bottom left) significantly lower than the corresponding original values (top right). (e) Significant peptides (|p(corr)| > 0.7, p > 0.05) mapped on the LXRβ LBD crystal structure (1PQC.pdb). SUS plots of the Gal4 LXRβ pEC50 vs. TG (f) and ABCA1 (g) OPLS-DA models showing that the pEC50 and TG but not ABCA1 models have peptides of equally high importance clustering along the diagonal. For clarity, peptides with low coefficients of contribution to the class separation for both models (p < 0.05) were excluded.

Related to Figure 6

Supplementary Note 1 Synthetic procedures

Content:

- **Synthesis Schemes**
- **General Information**
- **Synthesis Procedures for Compounds AZ1-AZ9**
- **References to previously published compounds**

Synthesis Schemes

Scheme S1. Synthesis of final compound **AZ1**

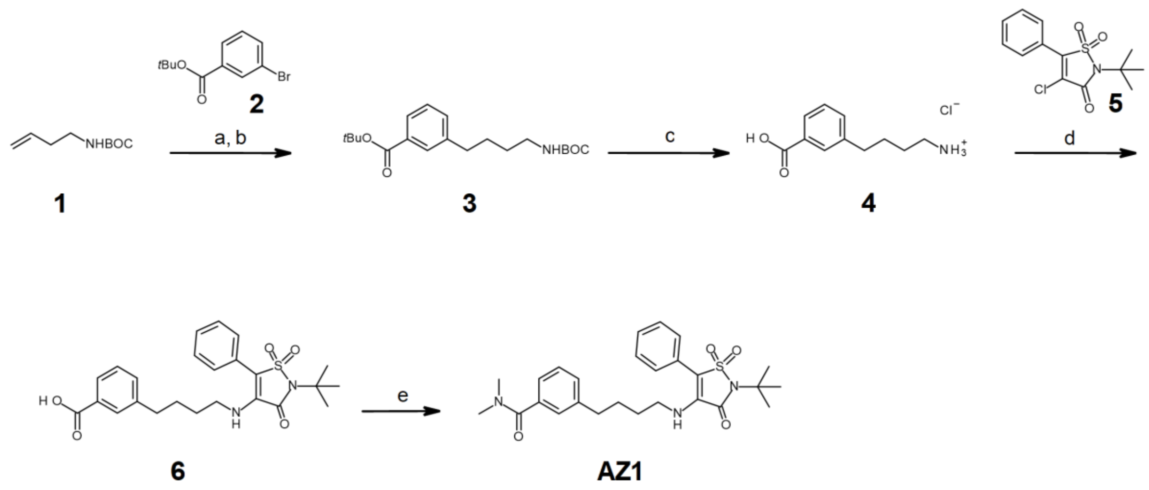

Reagents and conditions: (a) 0.4 M 9-BBN, dry THF, 0 ˚C–rt, 16 h; (b) 3 M K_3PO_4, 1,1'-bis (diphenylphosphino)ferrocene palladium (II) chloride, DMF, rt, 48 h; (c) 4 M HCl in dioxane, EtOAc, 24 h; (d) K_2CO_3, DMSO, 100 °C, 1 h, microwave heating; (e) TBTU, DIPEA, $(CH_3)_2NH$ x HCl, DMF, 1 h, rt.

Scheme S2. Synthesis of final compounds **AZ2** and **AZ3**

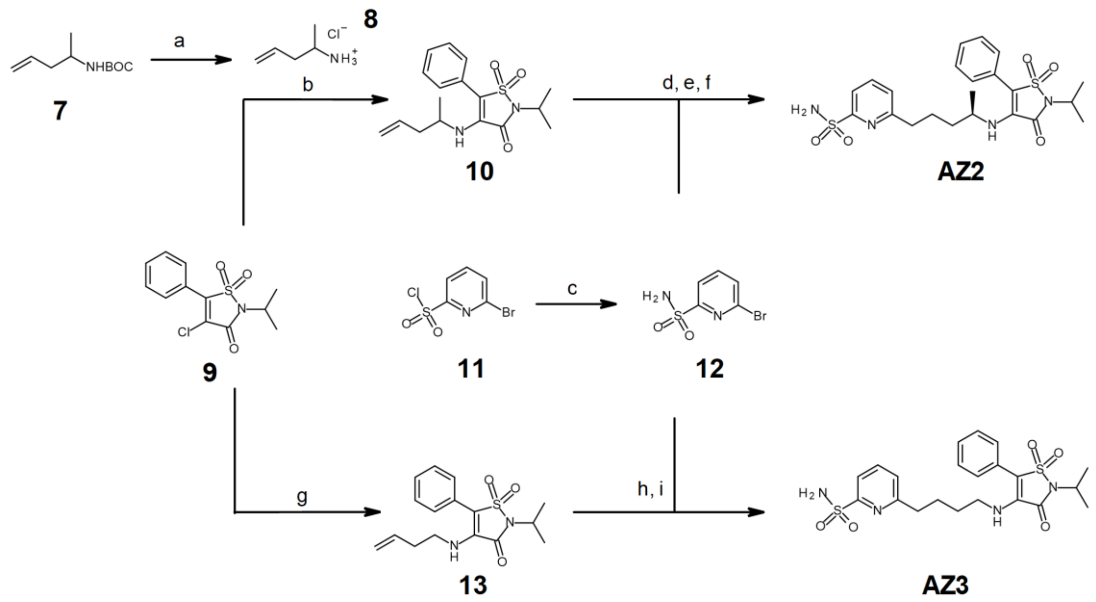

Reagents and conditions: (a) HCl in EtOAc (sat), rt, 6 h; (b) Et_3N, CH_3CN, 130 ˚C microwave heating, 40 min; (c) 2 M NH_3 in MeOH, THF, rt; (d) 0.5 M 9-BBN, dry THF, 0 ˚C–rt, 1 h; (e) 3 M K_3PO_4, 1,1'-bis (diphenylphosphino)ferrocene palladium (II) chloride, dry DMF, 110 ˚C microwave heating; 20 min; (f) chiral chromatography; (g) 3-buten-1-amine, Et_3N, CH_3CN, rt, 1.5 h; (h) 0.5 M 9-BBN, dry THF, 0 ˚C–rt, 2 h; (i) 3 M K_3PO_4, 1,1'-bis (diphenylphosphino)ferrocene palladium (II) chloride, THF, 90 ˚C microwave heating; 10 min.

Scheme S3. Synthesis of key intermediate for final compounds **AZ4**, **AZ6** and **AZ7**

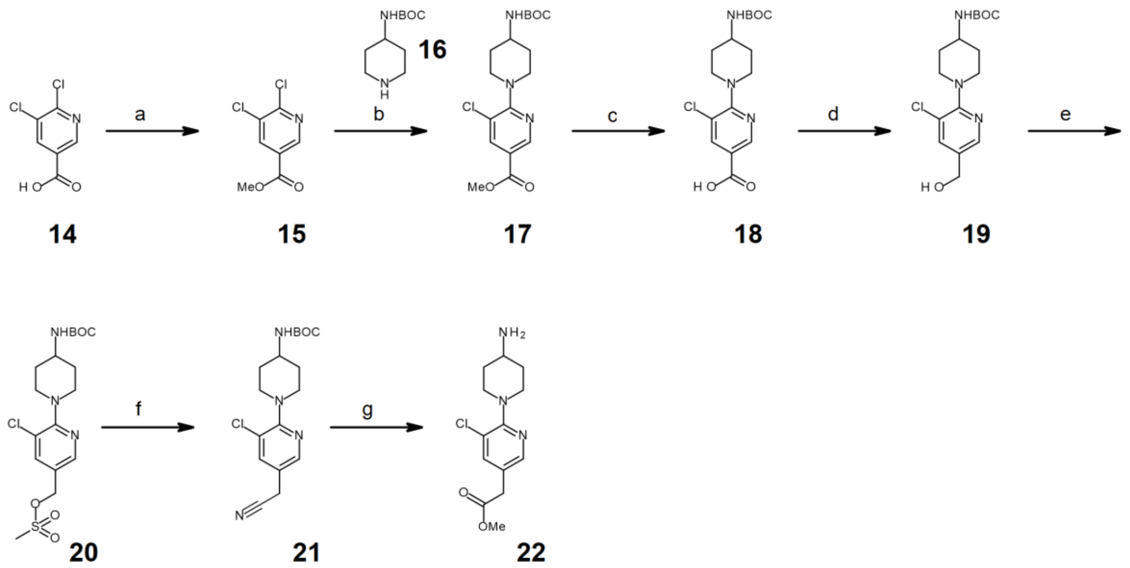

Reagents and conditions: (a) H_2SO_4, MeOH, reflux, 2.5 h; (b) DIPEA, DMSO, 150 ˚C microwave heating, 1 h; (c) LiOH, THF/water (1:1), rt, 3 h; (d) 1 M BH_3-THF complex , THF, -78 ˚C–rt, 4 h; (e) MsCl, Et_3N, DCM, rt, 4 h; (f) tetrabutylammonium cyanide; CH_3CN, rt, 2 h; (g) conc HCl, MeOH, reflux, 2 h.

Scheme S4. Synthesis of final compounds **AZ4**, **AZ6** and **AZ7**

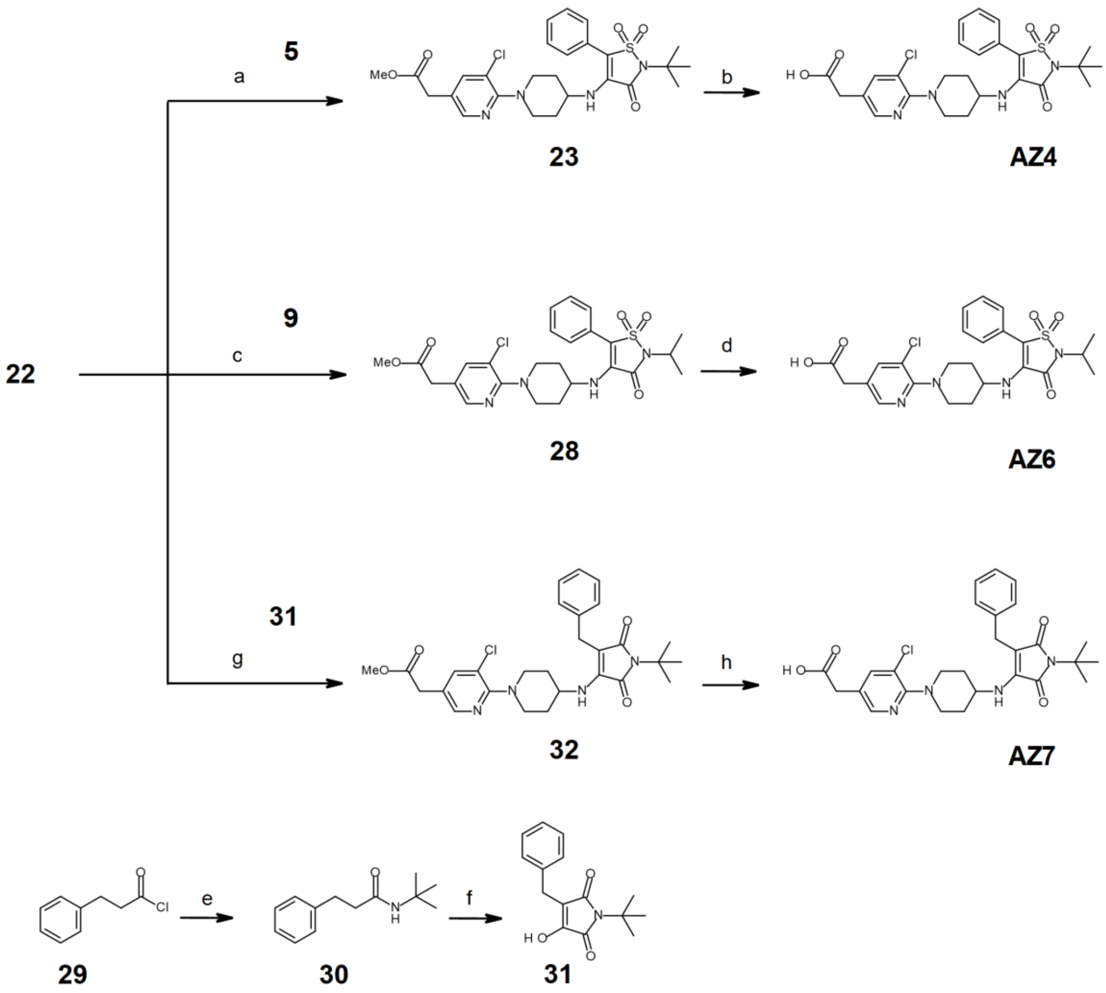

Reagents and conditions: (a) Et_3N, dry DMF, 120 ˚C microwave heating, 15 min; (b) LiI, pyridine, 130 ˚C microwave heating, 3 h; (c) Et_3N, dry DMF, 120 ˚C microwave heating, 20 min; (d) LiI, pyridine, 150 ˚C microwave heating, 1.5 h; (e) *tert*-butylamine, Et_3N, THF, 0 ˚C, 15 min; (f) diethyl oxalate, KOtBu, THF, 0–65 ˚C, 50 min; (g) 4-toluenesulfonic acid monohydrate, toluene/DMF, 120 ˚C, 50 min; (h) LiOH, THF/water, rt, 2 h.

Scheme S5. Synthesis of final compound **AZ5**

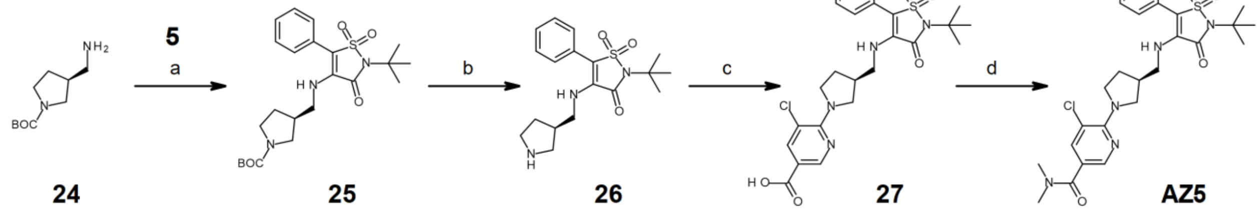

Reagents and conditions: (a) Et_3N, DMF, 130 ˚C microwave heating, 40 min; (b) TFA, DCM, rt, 1.5 h; (c) DIPEA, 5,6-dichloronicotinic acid, dry DMSO, 150 ˚C microwave heating, 1 h; (d) DIPEA, TBTU, dimethylamine hydrochloride, dry DMF, rt, 2 h.

Scheme S6. Synthesis of final compound **AZ8**

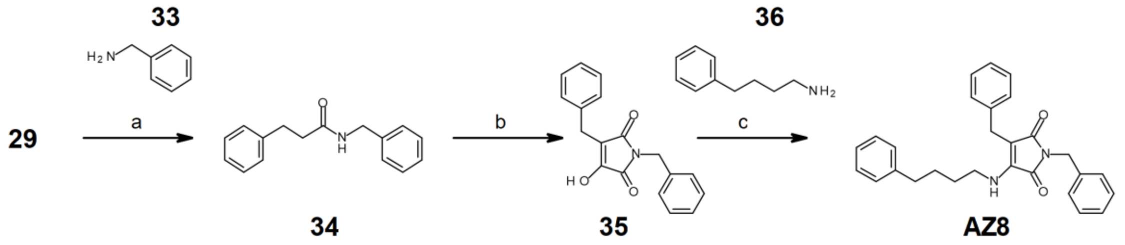

Reagents and conditions: (a) benzylamine, dry DCM, 0 ˚C, 2 h; (b) diethyl oxalate, KOtBu, dry THF, rt, 30 min; (c) 4-phenylbutylamine, 4-toluenesulfonic acid monohydrate, toluene/DMF, 120–130 ˚C microwave heating, 30–50 min.

Scheme S7. Synthesis of final compound **AZ9**

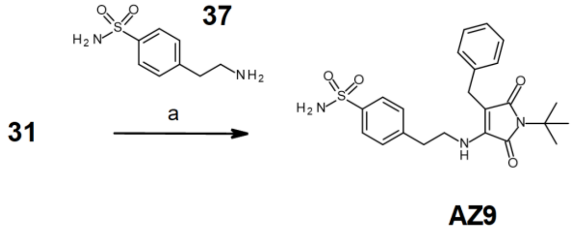

Reagents and conditions: (a) 4-toluenesulfonic acid monohydrate, dry toluene/DMF, 130 ˚C microwave heating, 40 min.

General Information

All reagents and solvents were obtained from commercially available suppliers and used without purification. Microwave heating was achieved in a Biotage initiator synthesiezer. Flash chromatography was performed on Biotage automated flash systems with UV detection using prepacked silica gel columns (SNAP Cartridge KP-Sil, 50 µm silica particles with a surface area of 500 m^2/g. Sizes from 10 g to 100 g silica was used) supplied by Biotage. The used phase separators was supplied from IST. Preparative HPLC was conducted on a Kromasil C18 column (10 µm 250 x 50 ID mm) using a variable gradient of acetonitrile in H_2O/CH_3CN/formic acid 95/5/0.2 buffer over 20–30 minutes with a flow of 100 mL/min. The instrument was combined with an UV/VIS detector 155 from Gilson. Analytical UHPLC/MS was obtained on a Waters Aquity system with a Waters SQD mass spectrometer. The UHPLC was equipped with a BEH C18 column (1.7 µm 2.1 × 50 mm) using a 46 mM ammonium carbonate/NH_3 buffer at pH 10

and a HSS C18 column (1.8 µm 2.1× 50 mm) using a 11 mM ammonium formate buffer at pH 3 with a flow rate of 1 mL/min. The mass spectrometer used electrospray ionization (ESI) in both positive and negative mode. ^{1} HNMR and ^{13}C NMR spectra were generated on 300, 400 or 500 MHz Bruker Avance spectrometers. Chemical shifts (δ) are given in parts per million (ppm), with the residual solvent signal used as reference. Coupling constants (*J*) are reported as Hz. NMR abbreviations are used as follows: br = broad, s = singlet, d = doublet, t = triplet, q = quartet, dd = double doublet, m = multiplet.

All final compounds **AZ1–AZ9** have a purity (UV) of >94 %.

Synthesis of final compound AZ1 (Scheme S1)

4-(3-Carboxyphenyl)butylammonium hydrochloride (4). 9-BBN (0.4 M in hexane, 8.75 mL, 3.50 mmol) was added to a solution of *tert*-butyl *N*-but-3-enylcarbamate (0.5 g, 2.92 mmol) in 10 mL dry THF whilst cooled in an ice bath. The reaction was allowed to warm to rt and left to stir overnight (16 h). 3 M K_3PO_4 (aq. 1.24 g, 5.84 mmol), *tert*-butyl 3-bromobenzoate (0.75 g, 2.92 mmol), 1,1'-bis (diphenyphosphino)ferrocene palladium (II) chloride (0.12 g, 0.15 mmol) and 2 mL DMF were added. The mixture was stirred at rt , under N_2 over the weekend. The reaction was concentrated under reduced pressure, EtOAc and brine was added. The organic phase was washed four times with brine, evaporated and then further purifed by flash chromatography (Biotage HPFC, Si-column) using a gradient of 0–10 % MTBE in toluene. Gave 0.97 g of *tert*-butyl 3-[4-(*tert*-butoxycarbonylamino)butyl]benzoate (**3**). To **3** (0.90 g, 2.59 mmol) in 8 mL EtOAc was added 8 mL of 4 M HCl in dioxane. The mixture was stirred at rt over night. The solid was filtered off, washed with EtOAc and dried under reduced pressure. Gave 0.46 g (99 %) of the title compound. ^{1}H NMR (500 MHz, $(CD_3)_2SO$) δ 12.90 (s, 1H), 7.93 (s, 3H), 7.73 – 7.83 (m, 2H), 7.45 – 7.52 (m, 1H), 7.36 – 7.45 (m, 1H), 2.79 (t, *J* = 7.4 Hz, 2H), 2.67 (t, *J* = 7.5 Hz, 2H), 1.48 – 1.75 (m, 4H).

2-tert-Butyl-4-chloro-1,1-dioxo-5-phenyl-isothiazol-3-one (5). Synthesis described in Li, L. et al. Derivatives of Isothiazole-3(2*H*)-one 1,1-dioxides as Liver X Receptor Modulators. PCT Int. Appl. WO 2006/73363, July 13th, 2006.

3-[4-[(2-tert-Butyl-1,1,3-trioxo-5-phenyl-isothiazol-4-yl)amino]butyl]benzoic acid (6). A mixture of **5** (0.3 g, 1.00 mmol), **4** (0.23 g, 1.00 mmol) and K_2CO_3 (0.42 g, 3.00 mmol) in 6 mL DMSO was heated at 100 °C for 1 h. More **5** (30 mg, 0.10 mmol) was added and the heating was continued at 100 °C for 15 min. The solvent was evaporated and EtOAc (5 mL), 1 N HCl (4 mL) and brine (2 mL) was added. The phases were seperated and the organic phase was washed again with a mixture of brine (2 mL) and 1 N HCl (1 mL). The organic phase was evaporated to dryness and the residue was purified by flash chromatography (Biotage HPFC, Si-column) using a gradient of 12–100 % EtOAc (containing 1 % AcOH) in toluene. Gave 0.17 g (37 %) of the title compound. ^{1}H NMR (500 MHz, $CDCl_3$) δ 7.92 – 7.99 (m, 1H), 7.8 – 7.87 (m, 1H), 7.47 – 7.53 (m, 2H), 7.37 – 7.47 (m, 4H), 7.31 – 7.36 (m, 1H), 5.25 (t, *J* = 6.0 Hz, 1H), 2.87 (q, *J* = 6.4 Hz, 2H), 2.53 (t, *J* = 7.0 Hz, 2H), 1.74 (s, 9H), 1.39 – 1.46 (m, 4H), OH peak overlaps with water peak.

3-[4-[(2-tert-Butyl-1,1,3-trioxo-5-phenyl-isothiazol-4-yl)amino]butyl]-N,N-dimethyl-benzamide (AZ1). TBTU (48 mg, 0.15 mmol) was added to a mixture of **6** (57 mg, 0.12 mmol) and DIPEA (65 µL, 0.37 mmol) in DMF. After preactivation over 15 min dimethylamine hydrochloride (15 mg, 0.19 mmol) was added and the reaction was stirred for 1 h. EtOAc was added and the organic phase was washed twice with brine, once with sat. $NaHCO_3$ and K_2CO_3 then again with brine. The organic layer was evaporated to dryness and the remaining crude was purified by flash chromatography (Biotage HPFC, Si-column) using an isocratic system of toluene/EtOAc, 1:2, to give 48 mg (79 %) of the title compound. ^{1}H NMR (500 MHz, $CDCl_3$) δ 7.46 – 7.52 (m, 2H), 7.4 – 7.46 (m, 3H), 7.26 – 7.32 (m, 1H), 7.19 – 7.24 (m, 1H), 7.08 – 7.17 (m, 2H), 5.25 (t, *J* = 6.0 Hz, 1H), 3.04 (d, *J* = 73.2 Hz, 6H), 2.77 – 2.91 (m, 2H), 2.41 – 2.56

(m, 2H), 1.73 (s, 9H), 1.34 – 1.47 (m, 4H). ^{13}C NMR (126 MHz, $CDCl_3$) δ 171.9, 159.9, 142.2, 136.7, 135.2, 131.8, 129.8, 129.7, 128.8, 128.5, 127.2, 125.3, 124.8, 107.1, 61.7, 44.1, 39.8, 35.6, 35.3, 29.2, 28.2, 27.8.

Synthesis of final compound AZ2 (Scheme S2)

tert-Butyl N-(1-methylbut-3-enyl)carbamate (7). Synthesis described in Veenstra S. J., Schmid P. One-pot Synthesis of Protected Homoallyl Amines. *Tetrahedron Letters*. **1997**, *38*, 997.

Pent-4-en-2-amine hydrochloride (8). Compound **7** (5.96 g, 32.17 mmol) was dissolved in 5 mL EtOAc and cooled on ice. 55 mL of HCl in EtOAc (sat.) was added. The reaction solution was stirred at rt for 6 h. The reaction was followed by TLC using EtOAc/heptane (40:60) as mobile phase (Rf: 0.7). The solvent was evaporated under reduced pressure to give 5.6 g (99 %) of the title compound as a light brown oil. ^{1}H NMR (500 MHz, $CDCl_3$) δ 8.26 (s, 2H), 5.62–5.78 (m, 1H), 5.09–5.23 (m, 2H), 3.22–3.36 (m, 1H), 2.41–2.54 (m, 1H), 2.26–2.38 (m, 1H), 1.33 (d, J = 6.6 Hz, 3H).

4-Chloro-2-isopropyl-1,1-dioxo-5-phenyl-isothiazol-3-one (9). Prepared as described in Li, L. et al. Derivatives of Isothiazole-3(2*H*)-one 1,1-dioxides as Liver X Receptor Modulators. PCT Int. Appl. WO 2006/73363, July 13th, 2006.

2-Isopropyl-4-(1-methylbut-3-enylamino)-1,1-dioxo-5-phenyl-isothiazol-3-one (10). A mixture of **8** (2 g, 12.65 mmol), **9** (3.62 g, 12.65 mmol) and Et_3N (7.02 mL, 50.61 mmol) in CH_3CN (40 mL) was heated in the microwave at 130 °C for 40 min. The black crude solution was evaporated. EtOAc was added. The organic phase was washed once with 1 M $NaHCO_3$, twice with water and once with brine, dried using a phase separator and evaporated. The crude was further purified by HPFC (Si-column) using a gradient of EtOAc/heptane, 0–20 % EtOAc, detection at 300 nm, to give 1.84 g (43 %, 5.50 mmol) of the title compound as an orange solid after evaporation of solvent. ^{1}H NMR (500 MHz, $CDCl_3$) δ 7.48–7.57 (m, 2H), 7.41–7.48 (m, 3H), 5.35–5.48 (m, 1H), 5.16 (d, J = 8.8 Hz, 1H), 4.97–5.06 (m, 1H), 4.85–4.97 (m, 1H), 4.33–

4.47 (m, 1H), 3.20–3.32 (m, 1H), 1.92–2.12 (m, 2H), 1.58 (d, J = 6.9 Hz, 6H), 0.97 (d, J = 6.5 Hz, 3H).

6-Bromopyridine-2-sulfonamide (12). 6-Bromopyridine-2-sulfonyl chloride (0.91 g, 3.02 mmol) was dissolved in THF (12 mL). 2 M NH_3 in MeOH (15 mL) was added. Gave a cloudy reaction that was stirred at rt for 2.5 h. More 2 M NH_3 in MeOH (6 mL) was added and the reaction mixture was stirred over night. The solvent was evaporated to give a white solid. EtOAc was added, the organic phase was washed with 1 M HCl and water, dried using a phase separator and evaporated. The compound was dissolved in DMSO and purified by preparative HPLC on a Kromasil C8 column (10 µm 250 x 50 ID mm) using a gradient of 10–60 % CH_3CN in H_2O/CH_3CN/formic acid 95:5:0.2 buffer over 25 min with a flow of 100 mL/min. The compounds were detected by UV at 230 nm. The CH_3CN was evaporated and the water phase was extracted twice with EtOAc, dried using a phase separator and evaporated to give 0.52 g (73 %, 2.20 mmol) the title compound as a white solid. ^{1}H NMR (500 MHz, CD_3OD δ 7.94–8.00 (m, 1H), 7.86–7.94 (m, 1H), 7.76–7.82 (m, 1H).

6-[Rel-(4R)-4-[(2-isopropyl-1,1,3-trioxo-5-phenyl-isothiazol-4-yl)amino]pentyl]pyridine-2-sulfonamide (AZ2). Compound **10** (1 g, 2.99 mmol) was dissolved in dry THF (21 mL) in a microwave vial and cooled in an ice-bath. (1s,5s)-9-Borabicyclo[3.3.1]nonane (0.5 M, 11.96 mL, 5.98 mmol) was added and the yellow reaction solution was stirred at rt for 1h. A solution of 3 M K_3PO_4 (1.90 g, 8.97 mmol, aq), **12** (0.71 g, 2.99 mmol) dissolved in dry DMF (3 mL) and 1,1'-bis (diphenylphosphino)ferrocene palladium (II) chloride (0.36 g, 0.45 mmol) were quickly added to the yellow reaction solution. The solution was evacuated and filled with nitrogen. The mixture was heated in the microwave for 20 min at 110 °C. The solvent was evaporated and DCM was added. The organic phase was washed with water, the organic phase was separated using a phase separator and evaporated. The crude was purified by HPFC (Biotage system, Si-column) using a gradient of 30–80 % EtOAc (containing 4 % 2 M NH_3 in

MeOH) in heptane. The compounds were detected at 310 nm. Gave 0.60 g (40 %, 1.22 mmol,) of the title compound, a white solid, as a racemic mixture after evaporation of solvents. The enantiomers were separated by chiral chromatography on a Chiralpak IA 250 mm x 20 mm, 5 μm HPLC column. 12 mg (10 mg/mL in DCM) was injected and eluted with heptane/EtOAc at a flow rate of 18 mL/min and detected at 300 nm. The second eluted compound was collected and evaporated to yield the title compound (269 mg, 99.7 % ee) $[a]^{20}_{D}$ +88.4 (c 1.0, CH_3CN). ^1H NMR (500 MHz, $CDCl_3$) δ 7.71–7.82 (m, 2H), 7.39–7.47 (m, 2H), 7.32–7.39 (m, 3H), 7.17–7.19 (m, 1H), 5.31 (d, J = 9.3 Hz, 1H), 5.18 (s, 2H), 4.26–4.40 (m, 1H), 3.09–3.24 (m, 1H), 2.54–2.76 (m, 2H), 1.45–1.56 (m, 8H), 1.16–1.39 (m, 2H), 0.89 (d, J = 6.4 Hz, 3H). ^{13}C NMR (126 MHz, $CDCl_3$) δ 161.4, 157.8, 157.1, 137.3, 133.7, 130.6, 128.8, 127.8, 125.2, 124.1, 117.2, 104.8, 48.0, 46.6, 35.7, 34.5, 24.4, 19.9, 19.2, 19.1. HRMS (ESI) m/z: 493.1567 (calcd for $C_{22}H_{29}N_4O_5S_2$ $[M+H]^+$, 493.1574).

Synthesis of final compound AZ3 (Scheme S2)

4-(But-3-enylamino)-2-isopropyl-1,1-dioxo-5-phenyl-isothiazol-3-one (13). Et_3N (5 mL, 36.07 mmol) was added to a solution of **9** (10 g, 35.00 mmol) and but-3-en-1-amine (2.74 g, 38.50 mmol) in CH_3CN (120 mL) at room temperature. The reaction was stirred at that temperatur for 1.5 h. The reaction was concentrated, dissolved in DCM and washed with 1 M $NaHCO_3$, 1 M HCl and water. The organic phase was filtered through a phase separator and concentrated. The brown crude was further purified by flash chromatography using a Biotage HPFC system (Si-column). Isocratic run with 15 % EtOAc (containing 4 % of 2 M NH_3 in MeOH) in heptane. Gave the title compound (5.96 g, 53 %, 18.60 mmol) as a light yellow solid after evaporation of solvent. ^1H NMR (500 MHz, $CDCl_3$) δ 7.50–7.61 (m, 2H), 7.43–7.50 (m, 3H), 5.48–5.66 (m, 1H), 5.24–5.40 (m, 1H), 5.06–5.12 (m, 1H), 4.99–5.06 (m, 1H), 4.32–4.51 (m, 1H), 2.87–2.97 (m, 2H), 2.10–2.21 (m, 2H), 1.60 (d, J = 6.9 Hz, 6H).

6-[4-[(2-Isopropyl-1,1,3-trioxo-5-phenyl-isothiazol-4-yl)amino]butyl]pyridine-2-sulfonamide (AZ3). Compound **13** (0.6 g, 1.87 mmol) was dissolved in dry THF (4 mL) and cooled on an icebath. 0.5 M (1*s*,5*s*)-9-borabicyclo[3.3.1]nonane (9.36 mL, 4.68 mmol) was added and the reaction stirred for 2 h warming to room temperature. To this, whilst bubbling N_2, was added sequentially, and quickly, a 3 M solution (aq) of K_3PO_4 (3.12 mL, 9.36 mmol), **12** (0.444 g, 1.87 mmol) as a solution in THF (3 mL) and finally 1,1'-bis (diphenylphosphino)ferrocene palladium (II) chloride (0.151 g, 0.19 mmol). The reaction solution was sealed in a microwave vial, evacuated and flushed with N_2 three times and then heated in the microwave at 90 °C for 10 minutes. To the reaction mixture was added DCM (60 mL) and water (30 mL), the organic phase was separated and the aqueous layer extracted again with DCM (30 mL). The organics were pooled, passed over a phase separator and reduced in vacuo. The resulting oil was taken up in DMSO (4 mL) and purified by preparative HPLC (Kromasil C8 column, 10 μm 250 x 50 ID mm) using a gradient of 20–60 % CH_3CN in H_2O/CH_3CN/acetic acid 95/5/0.2 buffer over 30 minutes with a flow of 100 mL/min. The compounds were detected by UV at 230 nm. The solvents were evaporated and the residue dissolved in DCM and purified on a 50 g Biotage SNAP flash silica column. The compound was eluted with a gradient of 0–100 % EtOAc in heptane over 20 CV. Gave the title compound (0.586 g, 65.4 %) as a white solid. ^1H NMR (500 MHz, $CDCl_3$) δ 7.77–7.89 (m, 2H), 7.45–7.53 (m, 2H), 7.38–7.45 (m, 3H), 7.27–7.29 (m, 1H), 5.65–5.74 (m, 1H), 5.31 (s, 2H), 4.34–4.47 (m, 1H), 2.89 (q, J = 6.4 Hz, 2H), 2.78 (t, J = 7.3 Hz, 2H), 1.60–1.67 (m, 3H), 1.57 (d, J = 6.9 Hz, 6H), 1.37–1.48 (m, 2H). The multiplett at 1.60–1.67 overlaps with the water peak. ^{13}C NMR (126 MHz, $CDCl_3$) δ 162.4, 158.8, 158.1, 138.4, 135.5, 131.6, 129.6, 128.6, 126.3, 125.1, 106.6, 47.6, 44.3, 36.5, 27.7, 26.2, 20.2. HRMS (ESI) m/z: 479.1425 (calcd for $C_{21}H_{27}N_4O_5S_2$ $[M+H]^+$, 479.1418).

Synthesis of final compound AZ4 (Scheme S3 and S4)

Methyl 6-[4-(tert-butoxycarbonylamino)-1-piperidyl]-5-chloro-pyridine-3-carboxylate (17). 5,6-Dichloronicotinic acid (3 g, 15.63 mmol) was suspended in MeOH (100 mL). Sulfuric acid (5 mL) was added and the mixture was heated to reflux for 2.5 h. The reaction mixture was cooled to rt and concentrated. DCM (100 mL) and 1 M NaOH (40 mL) was added. The phases were separated and the organic phase was washed with 1 M NaOH (40 mL), dried ($MgSO_4$) and concentrated to give 2.89 g (89 %, 14.03 mmol) of methyl 5,6-dichloropyridine-3-carboxylate (**15**) as a white solid. Compound **15** (1.39 g, 6.74 mmol) and boc-4-aminopiperidine (1.48 g, 7.42 mmol) was added to two microwave vials and dissolved in dry DMSO (13 mL). DIPEA (2.35 mL, 13.49 mmol) was added to each vial. The reaction mixtures were heated in the microwave for 1 h at 150 °C. The reaction mixtures were combined, diluted with DCM and washed with water using a phase separator. The solvent was evaporated and the crude was further purified by a Biotage SP1 TM HPFC system (Si-column) using isocratic conditions for 3 CV with 7 % EtOAc/trietylamine (99.5:0.5) in heptane/EtOAc/ Et_3N (90:9.5:0.5 followed by a gradient of 7–60 % EtOAc/ Et_3N (99.5:0.5) for 10 CV. Finally the title compound was eluted using isocratic conditions for 2 CV with 60 % EtOAc/ Et_3N (99.5:0.5). Gave 3.03 g (60 %, 8.19 mmol) of the title compound as a white solid. ^{1}H NMR (500 MHz, $CDCl_3$) δ 8.73 (d, *J* = 2.0 Hz, 1H), 8.12 (d, *J* = 2.0 Hz, 1H), 4.52 (bs, 1H), 4–4.13 (m, 2H), 3.91 (s, 3H), 3.74 (bs, 1H), 3–3.12 (m, 2H), 2.01–2.14 (m, 2H), 1.52–1.60 (m, 2H), 1.47 (s, 9H).

6-[4-(tert-Butoxycarbonylamino)-1-piperidyl]-5-chloro-pyridine-3-carboxylic acid (18). Compound **17** (3.02 g, 8.17 mmol) was dissolved in THF (22 mL) and water (22 mL). LiOH (0.25 g, 10.62 mmol) was added and the reaction was stirred for 3 h. The reaction solution was diluted with diethyl ether and neutralized with acetic acid (1 mL). The organic layer was separated, dried (Na_2SO_4), filtered and evaporated to give 3.09 g (99 %, 8.69 mmol) of the title compound as a white solid. LC−MS (ESI) *m/z*: 356 (calcd for $C_{16}H_{23}ClN_3O_4$ $[M+H]^+$, 356). ^{1}H NMR (500 MHz, CD_3OD) δ 8.68 (d, *J* = 1.9 Hz, 1H), 8.12 (d, *J* = 1.9 Hz, 1H), 3.99–4.12 (m,

2H), 3.53–3.66 (m, 1H), 2.97–3.14 (m, 2H), 1.91–2.05 (m, 2H), 1.53–1.69 (m, 2H), 1.45 (s, 9H).

tert-Butyl N-[1-[3-chloro-5-(hydroxymethyl)-2-pyridyl]-4-piperidyl]carbamate (19). Compound **18** (4.01 g, 11.27 mmol) was dissolved in dry THF (60 mL) and cooled in an isopropanol/CO_2 bath. 1 M borane THF-complex (45 mL, 45.08 mmol) was added slowly and dropwise. The reaction was allowed to slowly reach rt. After 4 h the reaction was quenched with MeOH (2 mL). The reaction was concentrated, diluted with DCM and washed with water using a phase separator. The organic phase was evaporated to give 3.78 g (98 %, 11.06 mmol) of the title compound as a white solid. LC−MS (ESI) *m/z*: 342 (calcd for $C_{16}H_{25}ClN_3O_3$ $[M+H]^+$, 342). ^1H NMR (500 MHz, CD_3OD) δ 8.12 (d, *J* = 1.6 Hz, 1H), 7.72 (d, *J* = 1.7 Hz, 1H), 4.55 (s, 2H), 3.66–3.79 (m, 2H), 3.47–3.61 (m, 1H), 2.82–2.97 (m, 2H), 1.90–2.03 (m, 2H), 1.57–1.72 (m, 2H), 1.47 (s, 9H).

[6-[4-(tert-Butoxycarbonylamino)-1-piperidyl]-5-chloro-3-pyridyl]methyl methanesulfonate (20). Compound **19** (3.78 g, 11.06 mmol) was dissolved in dry DCM (60 mL) and cooled in an ice-bath. Methanesulfonyl chloride (0.51 mL, 6.63 mmol) was added during stirring, followed by dropwise addition of Et_3N (1.15 mL, 8.29 mmol). The reaction was stirred at rt for 3 h. Additional methanesulfonyl chloride (0.51 mL, 6.63 mmol) and Et_3N (1.15 mL, 8.29 mmol) was added and the mixture was stirred at rt for 1 h. The reaction mixture was diluted with DCM, washed with sat. $NaHCO_3$ solution and water, using a phase separator, and evaporated. The crude was purified by flash chromatography using Biotage SP4 TM HPFC system (Si-column). The title compound was eluted using isocratic conditions with 5 % of EtOAc/Et_3N (99.5:0.5) in heptane/EtOAc/Et_3N (90:9.5:0.5) for 3 CV followed by a gradient for 10 CV with 5–80 % of EtOAc/Et_3N (99.5:0.5) and finally isocratic conditions for 2 CV with 80 % EtOAc/Et_3N (99.5:0.5). Gave 1.69 g (36 %, 4.04 mmol) of the title compound. ^1H NMR (500 MHz, $CDCl_3$)

δ 8.15 (d, *J* = 2.0 Hz, 1H), 7.65 (d, *J* = 2.1 Hz, 1H), 4.52 (s, 3H), 3.76–3.91 (m, 2H), 3.69 (s, 1H), 2.89–3.05 (m, 2H), 2.01–2.14 (m, 2H), 1.54–1.63 (m, 6H), 1.48 (s, 9H).

tert-Butyl N-[1-[3-chloro-5-(cyanomethyl)-2-pyridyl]-4-piperidyl]carbamate (21). Compound **20** (1.70 g, 4.04 mmol) was dissolved in dry CH_3CN (50 mL) and tetrabutylammonium cyanide (2.17 g, 8.08 mmol) was added. The mixture was stirred at rt for 2 h. The reaction mixture was evaporated, diluted with EtOAc and washed with water, dried ($MgSO_4$), filtered and evaporated to give 1.41 g (99 %, 4.03 mmol) of the title compound. ^{1}H NMR (400 MHz, $CDCl_3$) δ 8.06 (d, *J* = 2.2 Hz, 1H), 7.56 (d, *J* = 2.2 Hz, 1H), 4.47 (s, 1H), 3.72–3.82 (m, 2H), 3.63 (s, 3H), 2.86–2.98 (m, 2H), 1.98–2.10 (m, 2H), 1.48–1.61 (m, 3H), 1.43 (s, 9H).

Methyl 2-[6-[4-[(2-tert-butyl-1,1,3-trioxo-5-phenyl-isothiazol-4-yl)amino]-1-piperidyl]-5-chloro-3-pyridyl]acetate (23). Compound **21** (1.88 g, 5.36 mmol) was refluxed in MeOH (100 mL) and conc. HCl (100 mL) for 2 h. The MeOH was evaporated. The reaction mixture was neutralized to pH ~7 with 2 M Na_2CO_3 solution and solid K_2CO_3 then extracted with EtOAc, dried (Na_2SO_4), filtered and evaporated to give 1.07 g (70 %, 3.77 mmol) of methyl 2-[6-(4-amino-1-piperidyl)-5-chloro-3-pyridyl]acetate (**22**) as yellow oil. LC−MS (ESI) *m/z*: 284 (calcd for $C_{13}H_{19}ClN_3O_2$ $[M+H]^+$, 284). Compound **5** (1.43 g, 4.77 mmol) was dissolved in dry DMF (6 mL) in a microwave vial and cooled in an ice-bath. Compound **22** (1.49 g, 5.25 mmol) dissolved in dry DMF (9 mL) was added followed by Et_3N (0.80 mL, 5.72 mmol). The reaction mixture was heated in the microwave at 120 °C for 15 min. The mixture was diluted with DCM, washed with water, separated using a phase separator and evaporated. The crude product was purified with a SP4 TM HPFC system (Si-column (CV=120 mL), A = heptane/EtOAc/Et_3N (95:4.5:0.5), B = EtOAc/Et_3N (99.5:0.5), isocratic conditions with 6 % B for 2 CV followed by a gradient with 6–55 % B for 10 CV, finally isocratic conditions with 55 % B for 2 CV. Gave 2.19 g (83 %, 4.00 mmol) of the title compound as a light yellow solid after evaporation of solvents. LC−MS (ESI) *m/z*: 547 (calcd for $C_{26}H_{32}ClN_4O_5S$ $[M+H]^+$, 547). ^{1}H NMR (500 MHz,

$CDCl_3$) δ 7.98 (d, *J* = 2.1 Hz, 1H), 7.47–7.56 (m, 3H), 7.39–7.47 (m, 3H), 5.24 (d, *J* = 8.9 Hz, 1H), 3.69 (s, 3H), 3.52–3.61 (m, 2H), 3.50 (s, 2H), 2.96–3.17 (m, 1H), 2.31–2.47 (m, 2H), 1.69–1.80 (m, 11H), 1.45–1.55 (m, 2H).

2-[6-[4-[(2-tert-Butyl-1,1,3-trioxo-5-phenyl-isothiazol-4-yl)amino]-1-piperidyl]-5-chloro-3-pyridyl]acetic acid (AZ4). Three microwave vials were charged with **23** (0.69 g, 1.26 mmol) and anhydrous LiI (1.35 g, 10.9 mmol). Pyridine (11 mL) was added to each vial. The reactions were heated in the microwave at 130 °C for 1 h. Additional LiI (1.01 g, 7.57 mmol) was added to each vial and the reactions were heated in the microwave for 2 h at 130 °C. The reaction mixtures were combined, diluted with EtOAc, washed with 1 M HCl (aq) and dried ($MgSO_4$). The crude was purified by anion exchange chromatography (ISOLUTE NH_2, 10 g column). The column was rinsed with CH_3CN and MeOH, the product eluated with MeOH/AcOH (98:2) to give 0.93 g (46 %, 1.74 mmol) of the title compound. ^{1}H NMR (500 MHz, CD_3OD) δ 8.00 (d, *J* = 2.0 Hz, 1H), 7.65 (d, *J* = 2.0 Hz, 1H), 7.48–7.59 (m, 5H), 3.50–3.64 (m, 4H), 3.03–3.16 (m, 1H), 2.24–2.34 (m, 2H), 1.68–1.82 (m, 12H), 1.55–1.68 (m, 2H). ^{13}C NMR (126 MHz, CD_3OD) δ 159.5, 157.2, 145.8, 139.8, 134.7, 131.9, 129.5, 128.3, 125.7, 122.5, 105.7, 60.8, 50.3, 31.5, 26.4. HRMS (ESI) m/z: 533.1601 (calcd for $C_{25}H_{30}ClN_4O_5S$ $[M+H]^+$, 533.162).

Synthesis of final compound AZ5 (Scheme S5)

tert-Butyl (3S)-3-[[(2-tert-butyl-1,1,3-trioxo-5-phenyl-isothiazol-4-yl)amino]methyl]pyrrol-idine-1-carboxylate (25). Compound **5** (2 g, 6.67 mmol) was dissolved in dry DMF (15 mL). (*S*)-3-(Aminomethyl)-1-*N*-boc-pyrrolidine (1.60 g, 8.01 mmol) was added followed by Et_3N (1.09 mL, 8.01 mmol). The reaction mixture was heated in the microwave for 40 min at 130 ˚C. The mixture was diluted with DCM, washed with 1 M $NaHCO_3$ solution, 1 M HCl and water. The phases were separated using a phase separator. The crude product was purified with a Biotage HPFC system using isocratic conditions with EtOAc (containing a small amount of NH_3) in heptane (30:70). Gave the 2.31 g (74 %, 4.98 mmol) of the title compound as a white

solid. ^{1}H NMR (400 MHz, $CDCl_3$) δ 7.39–7.52 (m, 5H), 5.28 (s, 1H), 3.10–3.38 (m, 3H), 2.67–2.98 (m, 3H), 2.02–2.23 (m, 1H), 1.63–1.86 (m, 10H), 1.43 (s, 9H), 1.27–1.38 (m, 1H).

2-tert-Butyl-1,1-dioxo-5-phenyl-4-[[(3R)-pyrrolidin-3-yl]methylamino]isothiazol-3-one (26). Compound **25** (2.31 g, 4.98 mmol) was stirred in TFA (20 mL) and DCM (40 mL) at rt. After 1.5 h the solvent was evaporated and the crude was coevaporated with toluene twice. The residue was dissolved in DCM and washed with water, dried using a phase separator and evaporated to give a transparent oil. DCM was added which made the desired product precipitated as a white solid that was filtered off and dried. Gave 1.4 g (77 %, 3.85 mmol) of the title compound. ^{1}H NMR (400 MHz, CD_3OD) δ 7.42–7.59 (m, 5H), 3.04–3.23 (m, 4H), 2.89–3.03 (m, 2H), 2.60–2.69 (m, 1H), 2.18–2.29 (m, 1H), 1.71–1.82 (m, 1H), 1.68 (s, 9H), 1.32–1.48 (m, 2H).

6-[(3S)-3-[[(2-tert-Butyl-1,1,3-trioxo-5-phenyl-isothiazol-4-yl)amino]methyl]pyrrolidin-1-yl]-5-chloro-N,N-dimethyl-pyridine-3-carboxamide (AZ5). DIPEA (1.99 mL, 11.54 mmol) was added to a solution of **26** (1.4 g, 3.85 mmol) and 5,6-dichloronicotinic acid (0.81 g, 4.24 mmol) in dry DMSO (10 mL). The reaction mixture was heated in the microwave for 1 h at 150 ˚C. The reaction mixture was diluted with EtOAc and washed with 1 M HCl solution and water, dried using a phase separator and evaporated. Gave 1.68 g (58 %, 2.27 mmol, 70 % pure) of crude 6-[(3*S*)-3-[[(2-*tert*-butyl-1,1,3-trioxo-5-phenyl-isothiazol-4-yl)amino]methyl]pyrrolidin-1-yl]-5-chloro-pyridine-3-carboxylic acid (**27**) that was used directly. Compound **27** (1.68 g, 2.27 mmol), DIPEA (1.46 mL, 9.06 mmol) and TBTU (1.09 g, 3.40 mmol) were dissolved in dry DCM (35 mL). Dimethylamine hydrochloride (0.28 g, 3.40 mmol) was added. The mixture was stirred at rt for 2 h. The mixture was diluted with DCM and washed with 1 M HCl (aq), 1 M $NaHCO_3$ (aq) and water, dried using a phase separator and evaporated. The crude product was purified by flash chromatography (Biotage HPFC system) using an isocratic system of heptane/EtOAc containing 4 % NH_3 in EtOH, 40:60, followed by a second run using EtOAc

(with 4 % NH_3 in EtOH) /heptane (50:50). Gave 0.73 g of the title compound (59 %, 1.34 mmol) as a white solid after evaporation of solvents. ^{1}H NMR (500 MHz, $CDCl_3$) δ 8.16 (d, *J* = 2.0 Hz, 1H), 7.66 (d, *J* = 2.0 Hz, 1H), 7.49 – 7.53 (m, 2H), 7.42 – 7.47 (m, 3H), 5.33 – 5.49 (m, 1H), 3.66 – 3.77 (m, 2H), 3.63 (dd, *J* = 11.0, 7.0 Hz, 1H), 3.31 (dd, *J* = 11.0, 7.2 Hz, 1H), 3.10 (s, 6H), 2.90 – 3.04 (m, 2H), 2.18 – 2.30 (m, 1H), 1.86 – 1.96 (m, 1H), 1.75 (s, 9H), 1.40 – 1.52 (m, 1H). ^{13}C NMR (126 MHz, $CDCl_3$) δ 168.4, 159.5, 134.8, 131.6, 129.8, 128.7, 124.7, 121.8, 115.4, 61.6, 52.9, 49.1, 46.3, 38.3, 29.2, 27.6. HRMS (ESI) m/z: 546.1951 (calcd for $C_{26}H_{33}ClN_5O_4S$ $[M+H]^+$, 546.1937).

Synthesis of final compound AZ6 (Scheme S3 and S4)

Methyl 2-[5-chloro-6-[4-[(2-isopropyl-1,1,3-trioxo-5-phenyl-isothiazol-4-yl)amino]-1-piperidyl]-3-pyridyl]acetate (28). Compound **22** (0.66 g, 2.32 mmol), **9** (0.65 g, 2.27 mmol) and Et_3N (0.38 mL, 2.73 mmol) were dissolved in dry DMF (7 mL) and divided between two microwave vials. The reaction mixtures were heated in the microwave for 20 min at 120 °C. The mixtures were diluted with DCM and washed with water. The organic phase was separated using a phase separator, and evaporated. The crude product was further purified by a Biotage SP4 TM HPFC system. The title compound was eluted using isocratic conditions with heptane/EtOAc/Et_3N (90:9.5:0.5), for 3 CV followed by a gradient with 0–70 % EtOAc/Et_3N (99.5:0.5) in heptane/EtOAc/Et_3N (90:9.5:0.5). Gave 0.68 g (55 %, 1.27 mmol) of the title compound. ^{1}H NMR (500 MHz, $CDCl_3$) δ 7.99 (d, *J* = 2.0 Hz, 1H), 7.50–7.59 (m, 3H), 7.42–7.49 (m, 3H), 5.26 (d, *J* = 8.9 Hz, 1H), 4.35–4.49 (m, 1H), 3.70 (s, 3H), 3.54–3.65 (m, 2H), 3.51 (s, 2H), 3.06–3.21 (m, 1H), 2.36–2.52 (m, 2H), 1.73–1.82 (m, 2H), 1.59 (d, *J* = 6.9 Hz, 6H), 1.48–1.55 (m, 2H).

2-[5-Chloro-6-[4-[(2-isopropyl-1,1,3-trioxo-5-phenyl-isothiazol-4-yl)amino]-1-piperidyl]-3-pyridyl]acetic acid (AZ6). Two microwave vials were charged with **28** (0.17 g, 0.32 mmol) and LiI (0.69 g, 5.16 mmol). Pyridine (3.5 mL) was added to each vial. The vials were heated in the

microwave oven for 1 h at 150 °C. Additional LiI (0.17 g, 1.29 mmol) was added to each vial as LCMS showed starting material left, and the reactions were heated in the microwave for 30 min at 150 °C. The reaction mixtures were combined, diluted with EtOAc, washed with 1 M HCl solution and dried using $MgSO_4$. The crude was further purified by anion exchange chromatography using an Isolute PEAX column (5 g). The column was rinsed with DCM and CH_3CN followed by DCM/MeOH (99:1). The product was eluated with DCM/MeOH/AcOH (92:5:2) to give 0.28 g (83 %, 0.54 mmol) of the title compound. ^1H NMR (500 MHz, $CDCl_3$) δ 7.99 (s, 1H), 7.48–7.58 (m, 3H), 7.40–7.48 (m, 3H), 5.28 (d, J = 8.6 Hz, 1H), 4.33–4.47 (m, 1H), 3.56 (d, J = 12.4 Hz, 2H), 3.50 (s, 2H), 3.05–3.19 (m, 1H), 2.35–2.46 (m, 2H), 1.70–1.81 (m, 2H), 1.46–1.62 (m, 8H). ^{13}C NMR (126 MHz, $CDCl_3$) δ 172.9, 157.6, 156.3, 144.8, 138.8, 133.3, 130.5, 127.7, 124.0, 123.2, 121.7, 105.5, 49.2, 46.6, 39.5, 35.8, 31.2, 19.1. HRMS (ESI) m/z: 519.146 (calcd for $C_{24}H_{28}ClN_4O_5S$ $[M+H]^+$, 519.1464).

Synthesis of final compound AZ7 (Scheme S3 and S4)

N-tert-Butyl-3-phenyl-propanamide (30). Hydrocinnamoyl chloride (13.21 mL, 88.96 mmol) was added dropwise to a solution of *tert*-butylamine (11.22 mL, 0.11 mol) and Et_3N (14.88 mL, 0.11 mol) in THF (100 mL) at 0 ˚C under N_2. After stirring for 15 min the mixture was concentrated. The residue was dissolved in EtOAc and extracted with sat. aq. $NaHCO_3$-solution. Evaporation of the solvent gave 16.78 g (91 %, 81.73 mmol) of the desired product as a white solid. ^1H NMR (500 MHz, $CDCl_3$) δ 7.28–7.32 (m, 2H), 7.17–7.25 (m, 3H), 5.10 (bs, 1H), 2.96 (t, J = 7.6 Hz, 2H), 2.35–2.45 (m, 2H), 1.30 (s, 9H).

3-Benzyl-1-tert-butyl-4-hydroxy-pyrrole-2,5-dione (31). Compound **30** (9.37 g, 45.64 mmol) was dissolved in 200 mL dry THF and diethyl oxalate (27.90 mL, 0.21 mol) was added. The mixture was cooled in an ice-bath. Under N_2 atmosphere, KOtBu (23.05 g, 0.21 mol) was added carefully. After the addition, the ice-bath was removed and the mixture was stirred for 20 min, then heated to 65 °C for 30 min. The THF was evaporated. EtOAc (150 mL) was added to the

residue. The resulting mixture was cooled in an ice bath and water (20 mL) was added, followed by 10 % HCl until pH 1. The aqueos phase was exctracted three times with EtOAc (20 mL). The organic phases were combined, dried ($MgSO_4$) and evaporated to give 17 g of crude product that was purified by column chromatography (ISOLUTE Si-column, 70 g/150 mL x 2) using a gradient of EtOAc in heptane , 5–75 % EtOAc, to give 10.6 g (99 %, 41.1 mmol) of the title compound as a solid. ^{1}H NMR (400 MHz, $(CD_3)_2SO$) δ 11.87 (s, 1H), 7.21–7.31 (m, 2H), 7.12–7.21 (m, 3H), 3.48 (s, 2H), 1.47 (s, 9H).

Methyl 2-[6-[4-[(4-benzyl-1-tert-butyl-2,5-dioxo-pyrrol-3-yl)amino]-1-piperidyl]-5-chloro-3-pyridyl]acetate (32). In two microwave vials were added **22** (500 mg, 1.76 mmol), **31** (450 mg, 1.76 mmol), catalytic amounts of 4-toluenesulfonic acid, monohydrate, toluene (10 mL) and DMF (1 mL). The reaction mixtures were heated at 120 ˚C for 50 min. The reaction mixtures were concentrated. To the residues were added EtOAc and sat. aq. $NaHCO_3$ solution, the organic phases were separated, dried and concentrated. The crude products were purified using two Si-columns with an isocratic system of heptane/EtOAc 80:20, Rf = 0.1, to give totally 1.24 g (68 %, 2.36 mmol) of the desired product. ^{1}H NMR (500 MHz, $CDCl_3$) δ 8.02 (d, J = 2.0 Hz, 1H), 7.54 (d, J = 2.0 Hz, 1H), 7.23–7.33 (m, 2H), 7.12–7.19 (m, 3H), 5.07 (d, J = 9.7 Hz, 1H), 3.61–3.73 (m, 7H), 3.52 (s, 2H), 3.39–3.50 (m, 1H), 2.63–2.76 (m, 2H), 1.70–1.80 (m, 2H), 1.51–1.63 (m, 11H).

2-[6-[4-[(4-Benzyl-1-tert-butyl-2,5-dioxo-pyrrol-3-yl)amino]-1-piperidyl]-5-chloro-3-pyridyl]acetic acid (AZ7). Compound **32** (1.25 g, 2.38 mmol) was dissolved in THF (14 mL). A solution of lithium hyroxide monohydrate (0.60 g, 14.28 mmol) in water (3.5 mL) was added during stirring. The reaction mixture was stirred at rt for 2 h. The THF was evaporated. HCl (0.1 M) was added dropwise to the residue until the precipitation stopped. The aqueous layer was extracted with EtOAc. The organic layer was dried using Na_2SO_4, filtered and evaporated. The crude was purified by preparative HPLC (Kromasil C8 10 µm, 250 mm x 50 mm column

with a flow of 100 mL/min) using a gradient of 40–60 % CH_3CN in H_2O/CH_3CN/formic acid (95:5:0.2) buffer over 30 min. Gave 1.08 g (88 %, 2.11 mmol) of the title compound as yellow solid. **AZ7** (100 mg, 0.20 mmol) was dissolved in a small amount of EtOAc by heating. Heptane was added (EtOAc/heptane, 3:1). The solution was left at room temperature, in open air. Gave 75 mg (75 %, 0.15 mmol) of the title compound as a crystalline yellow solid. ^1H NMR (500 MHz, $CDCl_3$) δ 8.07 (s, 1H), 7.58 (s, 1H), 7.26–7.35 (m, 3H) overlaps with solvent peak, 7.15–7.25 (m, 3H), 5.11 (d, *J* = 9.5 Hz, 1H), 3.63–3.78 (m, 4H), 3.59 (s, 2H), 3.41–3.54 (m, 1H), 2.73 (t, *J* = 12.0 Hz, 2H), 1.70–1.85 (m, 2H), 1.52–1.67 (m, 11H). ^{13}C NMR (126 MHz, $CDCl_3$) δ 175.4, 175.2, 169.2, 157.5, 141.0, 140.3, 139.9, 128.7, 127.9, 126.3, 123.5, 122.7, 95.9, 57.0, 47.9, 36.6, 33.4, 29.2, 27.6. HRMS (ESI) m/z: 511.2099 (calcd for $C_{27}H_{32}ClN_4O_4$ $[M+H]^+$, 511.2107).

Synthesis of final compound AZ8 (Scheme S6)

N-Benzyl-3-phenylpropanamide (34). Hydrocinnamoyl chloride (8.81 mL, 59.30 mmol) was dissolved in dry DCM (100 mL) and cooled to 0 °C with an icebath. Benzylamine (12.94 mL, 0.12 mol) was added and the reaction was stirred at 0 °C for 2 h. The solvent was evaporated and the crude product was dissolved in EtOAc and washed with water (300 mL) and 1 % HCl (300 mL, aq.). The organic phase was dried (Na_2SO_4), filtered and concentrated. The crude product was purified with column chromatography on silica gel using heptane/EtOAc (80:20) as eluent. Gave 8.0 g (56 %, 33.43 mmol) of the title compound. ^1H NMR (500 MHz, $CDCl_3$) δ 7.14–7.35 (m, 10H), 5.66 (s, 1H), 4.42 (d, *J* = 5.6 Hz, 2H), 3.02 (t, *J* = 7.6 Hz, 2H), 2.54 (t, *J* = 7.6 Hz, 2H).

1,3-Dibenzyl-4-hydroxy-1H-pyrrole-2,5-dione (35). Compound **34** (3.72 g, 15.54 mmol) was dissolved in dry THF (50 mL). Diethyl oxalate (9.5 mL, 69.95 mmol) and KOtBu (7.85 g, 69.95 mmol) was added and the reaction was stirred at rt for 30 min. The reaction mixture was acidified with 2 M HCl to pH 3 and diluted with water. The water phase was extracted with

EtOAc (2 x 200 mL), the combined organic phases was dried (Na_2SO_4), filtered and evaporated. The crude product was purified with column chromatography on silica gel using heptane/EtOAc (90:10) as eluent. Gave 1.63 g (35 %, 5.56 mmol) of the title compound. ^{1}H NMR (400 MHz, $(CD_3)_2SO$) δ 12.38 (bs, 1H), 7.10–7.38 (m, 10H), 4.54 (s, 2H), 3.57 (s, 2H).

1,3-Dibenzyl-4-(4-phenylbutylamino)pyrrole-2,5-dione (AZ8). Compound **35** (0.5 g, 1.70 mmol), 4-phenylbutylamine (351 µl, 2.22 mmol) and 4-toluenesulfonic acid, monohydrate (32 mg, 0.17 mmol) was dissolved in toluene/DMF (4:1) and the reaction was run in the microwave oven at 120 ˚C for 20 min and then at 130 ˚C for 50 min. The solvents were evaporated and the crude was dissolved in EtOAc (200 mL). The organic phase was washed with 1 % HCl (aq) solution, dried (Na_2SO_4), filtered and evaporated. The crude was further purified by flash chromatography using a Biotage SP1 system. The compound was eluted with heptane/EtOAc (90:10). Gave 0.5 g (69 %, 1.18 mmol) of the titel compound. ^{1}H NMR (500 MHz, $CDCl_3$) δ 7.24–7.37 (m, 9H), 7.13–7.22 (m, 4H), 7.08–7.13 (m, 2H), 5.02–5.13 (m, 1H), 4.65 (s, 2H), 3.72 (s, 2H), 3.19–3.31 (m, 2H), 2.52 (t, J = 7.4 Hz, 2H), 1.50–1.57 (m, 2H), 1.42–1.50 (m, 2H). The multiplett at 1.42–1.50 overlaps with the water peak. ^{13}C NMR (126 MHz, $CDCl_3$) δ 168.0, 143.0, 141.6, 140.5, 136.9, 128.7, 128.6, 128.4, 128.3, 127.9, 127.6, 126.3, 126.0, 43.7, 41.6, 35.3, 29.9, 28.2, 27.5. HRMS (ESI) m/z: 425.2229 (calcd for $C_{28}H_{29}N_2O_2$ $[M+H]^+$, 425.2224).

Synthesis of final compound AZ9 (Scheme S7)

4-[2-[(4-Benzyl-1-tert-butyl-2,5-dioxo-pyrrol-3-yl)amino]ethyl]benzenesulfonamide (AZ9). Compound **31** (0.66 g, 2.53 mmol), 4-(2-aminoethyl)benzensulfonamide (0.66 g, 3.29 mmol) and 4-toluenesulfonic acid monohydrate (48 mg, 0.25 mmol) was dissolved in a mixture of dry toluene/DMF (3:1, 15 mL). The reaction was run in the microwave oven at 130 °C for 40 min. Toluene was evaporated and the crude was dissolved in water (200 mL). The pH of the water phase was set to 2 using 1 % HCl (aq). The water phase was extracted with EtOAc (200 mL),

the organic phase was dried using Na_2SO_4, filtered and evaporated. The crude was further purified by flash chromatography on a Biotage system (Biotage SP4) using heptane/EtOAc 65:35 as eluent to give 0.69 g (61 %, 1.56 mmol) of the title compound as a crystalline solid. Mp: 142 °C. ^{1}H NMR (500 MHz, $CDCl_3$) δ 7.83 (d, *J* = 8.3 Hz, 2H), 7.28–7.34 (m, 2H), 7.18–7.25 (m, 1H), 7.11–7.18 (m, 4H), 5.01–5.11 (m, 1H), 4.89 (s, 2H), 3.71 (s, 2H), 3.50 (q, *J* = 6.9 Hz, 2H), 2.79 (t, *J* = 7.0 Hz, 2H), 1.58 (s, 9H). ^{13}C NMR (126 MHz, $CDCl_3$) δ 175.1, 169.0, 143.1, 141.8, 140.5, 140.3, 129.4, 128.8, 127.8, 126.9, 126.3, 96.9, 57.1, 44.2, 36.5, 29.1, 27.3. HRMS (ESI) m/z: 442.1806 (calcd for $C_{23}H_{28}N_3O_4S$ $[M+H]^+$, 442.1795).

References to synthesis of previously published compounds

Synthesis for previously published compounds are described in the following references.

BMS-852927

Kick, E. K. *et al.* Discovery of Highly Potent Liver X Receptor beta Agonists. *ACS Med Chem Lett* **7**, 1207-1212, doi:10.1021/acsmedchemlett.6b00234 (2016).

WAY-252623

Wrobel, J. *et al.* Indazole-based liver X receptor (LXR) modulators with maintained atherosclerotic lesion reduction activity but diminished stimulation of hepatic triglyceride synthesis. *J Med Chem* **51**, 7161-7168, doi:10.1021/jm800799q (2008).

WAY-254011

Hu, B. *et al.* Discovery of phenyl acetic acid substituted quinolines as novel liver X receptor agonists for the treatment of atherosclerosis. *J Med Chem* **49**, 6151-6154, doi:10.1021/jm0609566 (2006).

GW-3965

Collins, J. L. *et al.* Identification of a nonsteroidal liver X receptor agonist through parallel array synthesis of tertiary amines. *J Med Chem* **45**, 1963-1966 (2002).

T 0901317

Li, L. *et al.* Preparation of bis(trifluoromethyl)hydroxymethylbenzenesulfonamides, -ureas, and -carbamates as liver X receptor modulators. WO2000054759A2 (2000).

F1

Lebreton, L., Dumas, C., Massardier, C. & Bondoux, M. New sulfonylindoline derivative LXR receptor modulators, their preparation, and their therapeutic use. FR2886293A1 (2006).

AZ876

Bostroem, J. *et al.* Preparation of non-anilinic isothiazol-3(2H)-one 1,1-dioxide derivatives for use as Liver X Receptor modulators. WO2006073363A1 (2006).
