# Supplementary material for: Structural analysis identifies an escape route from the adverse lipogenic effects of liver X receptor ligands
Source: Commun Biol. 2019 Nov 22;2:431. doi: 10.1038/s42003-019-0675-0 (PMC6874530; doi:10.1038/s42003-019-0675-0)
Supplement: Supplementary file 2 — Reporting Summary [file 42003_2019_675_MOESM2_ESM.pdf]

nature research

Corresponding author(s): Anna Y. Belorusova
Emma Evertsson

Last updated by author(s): Oct 3, 2019

Reporting Summary

Nature Research wishes to improve the reproducibility of the work that we publish. This form provides structure for consistency and transparency in reporting. For further information on Nature Research policies, see Authors & Referees and the Editorial Policy Checklist.

Statistics

For all statistical analyses, confirm that the following items are present in the figure legend, table legend, main text, or Methods section.

n/a	Confirmed	
☐	☒	The exact sample size (*n*) for each experimental group/condition, given as a discrete number and unit of measurement
☐	☒	A statement on whether measurements were taken from distinct samples or whether the same sample was measured repeatedly
☐	☒	The statistical test(s) used AND whether they are one- or two-sided<br>*Only common tests should be described solely by name; describe more complex techniques in the Methods section.*
☒	☐	A description of all covariates tested
☒	☐	A description of any assumptions or corrections, such as tests of normality and adjustment for multiple comparisons
☐	☒	A full description of the statistical parameters including central tendency (e.g. means) or other basic estimates (e.g. regression coefficient) AND variation (e.g. standard deviation) or associated estimates of uncertainty (e.g. confidence intervals)
☐	☒	For null hypothesis testing, the test statistic (e.g. *F*, *t*, *r*) with confidence intervals, effect sizes, degrees of freedom and *P* value noted<br>*Give P values as exact values whenever suitable.*
☒	☐	For Bayesian analysis, information on the choice of priors and Markov chain Monte Carlo settings
☒	☐	For hierarchical and complex designs, identification of the appropriate level for tests and full reporting of outcomes
☒	☐	Estimates of effect sizes (e.g. Cohen's *d*, Pearson's *r*), indicating how they were calculated

Our web collection on statistics for biologists contains articles on many of the points above.

Software and code

Policy information about availability of computer code

Data collection	MassLynx (v. 4.1, Waters) was used for controlling the Synapt G2-Si mass spectrometer for the acquisition of MS and MS/MS data.
Data analysis	ProteinLynx Global Server (v. 3.0, Waters) was used for processing MS/MS data. DynamX (v. 3.0, Waters) was used for the processing of HDX-MS data. The mean deuteration level per amino acid was calculated using Matlab (Mathworks). Pharmacological and SPR data was analysed using GraphPad Prism 8, Microsoft Excel and XLfit (ID Business Solutions). SIMCA 15 (Sartorius Stedim Data Analytics) was used for multivariate analysis. Modeling of in vivo data was done in Phoenix NMLE 1.3 (Certara). For the crystal structure determination, data sets were processed using Mosflm and Scala. Structures were solved by molecular replacement using programs form the CCP4 suite. Refinement was performed by manual rebuilding in Coot and automatic refinement using Refmac5 or CNS. PyMOL (Schrodinger LLC) was used for preparation of structural figures. For structural representation of HDX-MS data, missing loops were modelled with MOE (Chemical Computing Group Inc.).

For manuscripts utilizing custom algorithms or software that are central to the research but not yet described in published literature, software must be made available to editors/reviewers. We strongly encourage code deposition in a community repository (e.g. GitHub). See the Nature Research guidelines for submitting code & software for further information.

Data

Policy information about availability of data

All manuscripts must include a data availability statement. This statement should provide the following information, where applicable:
- Accession codes, unique identifiers, or web links for publicly available datasets
- A list of figures that have associated raw data
- A description of any restrictions on data availability

Atomic coordinates and related structure factors have been deposited in the Protein Data Bank with accession codes: 6S4T (LXRβ in complex with WAY-254011), 6S4U (LXRβ in complex with AZ3), 6S4N (LXRβ in complex with AZ6) and 6S5K (LXRβ in complex with AZ8). All other data generated or analyzed during the study in this published article (and its supplementary information files) are available upon reasonable request.

Field-specific reporting

Please select the one below that is the best fit for your research. If you are not sure, read the appropriate sections before making your selection.

☒ Life sciences ☐ Behavioural & social sciences ☐ Ecological, evolutionary & environmental sciences

For a reference copy of the document with all sections, see nature.com/documents/nr-reporting-summary-flat.pdf

Life sciences study design

All studies must disclose on these points even when the disclosure is negative.

Sample size	Sample sizes were chosen on the basis of standard practice performed for similar experiments and statistical methods were not used to predetermine sample sizes.
Data exclusions	Peptides with an intensity lower than 5000, a mass error >5 ppm and present in less than two of the three data acquisitions were excluded from the HDX-MS analysis. RNA samples with evidence of substantial degradation were excluded from the study.
Replication	HDX-MS experiments were performed for one biological replicate (protein batch) and three technical replicates. Two to six biological replicates were performed for pharmacological experiments. The number of replicates for each specific experiment is indicated throughout the manuscript text and figure legends.
Randomization	Animals were randomized to receive the experimental drug or vehicle. HDX-MS samples were measured according to the time point in the descending order (longer deuteration times followed by shorter time points). Compounds were measured in a random order within the individual time points.
Blinding	No blinding of experiments was required, as non of the experimental outcomes was based on (potentially biased) human judgment

Reporting for specific materials, systems and methods

We require information from authors about some types of materials, experimental systems and methods used in many studies. Here, indicate whether each material, system or method listed is relevant to your study. If you are not sure if a list item applies to your research, read the appropriate section before selecting a response.

Materials & experimental systems

n/a	Involved in the study	
☒	☐	Antibodies
☐	☒	Eukaryotic cell lines
☒	☐	Palaeontology
☐	☒	Animals and other organisms
☒	☐	Human research participants
☒	☐	Clinical data

Methods

n/a	Involved in the study	
☒	☐	ChIP-seq
☒	☐	Flow cytometry
☒	☐	MRI-based neuroimaging

Eukaryotic cell lines

Policy information about cell lines

Cell line source(s)	U2OS: ATCC, USA.
Authentication	The cell line was unauthenticated.
Mycoplasma contamination	The cell line was regularly tested for absence of mycoplasma contamination.
Commonly misidentified lines (See ICLAC register)	N/A

Animals and other organisms

Policy information about studies involving animals; ARRIVE guidelines recommended for reporting animal research

Laboratory animals	Male 7-8 week C57BL/6J mice.
Wild animals	The study involved no wild animals.

Field-collected samples	The study involved no field collected samples.
Ethics oversight	Animal experiments were approved by the respective local animal ethics committees (Gothenburg region Local Ethics Review Committee on Animal experiments and University of Virginia Animal Care and Use Committee).

Note that full information on the approval of the study protocol must also be provided in the manuscript.
